# Effect of Precursors on Trimetallic Ruthenium-Based Catalysts Supported on γ-Al_2_O_3_ Pellets for Low-Temperature Ammonia Decomposition

**DOI:** 10.1021/acsomega.4c09968

**Published:** 2025-04-10

**Authors:** Christopher J. Koch, Jennifer Naglic, Logan Kearney, Daniel Clairmonte, Binod Rai, Jochen Lauterbach, Lucas M. Angelette, Tyler Guin

**Affiliations:** † Hydrogen Isotope Processing Science, Savannah River National Laboratory, Aiken, South Carolina 29803, United States; + Department of Chemical Engineering, University of South Carolina, 541 Main St., Columbia, South Carolina 29208, United States; § Chemical Sciences Division, Oak Ridge National Laboratory, 1 Bethel Valley Road, Oak Ridge, Tennessee 37831, United States

## Abstract

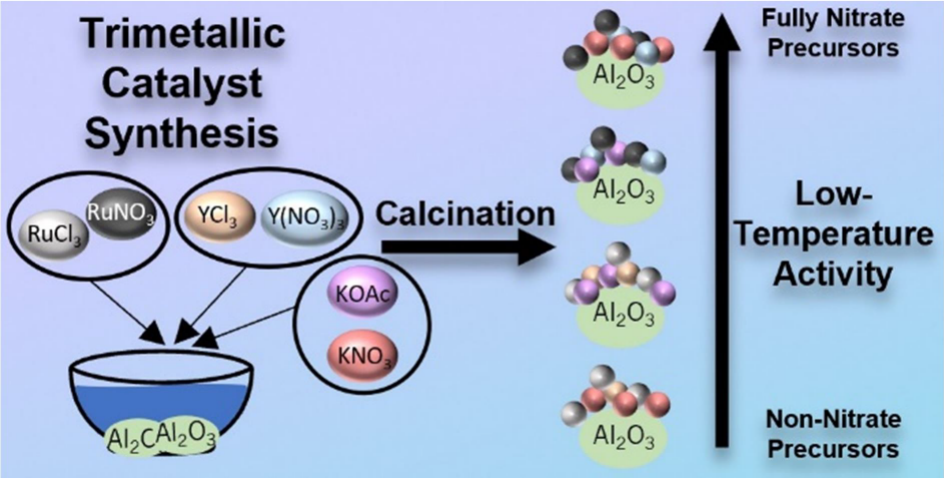

Ammonia is a promising candidate as a liquid hydrogen energy storage medium, but it requires catalytic decomposition (ammonia cracking) to regenerate hydrogen. Recently developed trimetallic ruthenium–potassium-promoter (RuKM) ammonia decomposition catalysts have exceptionally low ammonia decomposition temperatures, able to perform the decomposition as low as 250 °C, which is significantly lower than other known catalysts that require temperatures above 500 °C. However, the effects of the RuKM precursor on the catalytic activity have not been investigated. We report the observed differences of 3% ruthenium/12% potassium/1% yttrium (RuKY) catalysts on γ-alumina synthesized from chloride-, nitrate-, and acetate-based precursors. Catalysts synthesized from chloride-based precursors demonstrated the lowest ammonia decomposition catalytic activity at lower reaction temperatures. In contrast, those synthesized from nitrate-based precursors demonstrated the highest yield, despite similar metal loading. This difference in reactivity is most apparent between 250 and 400 °C, as the conversion rates of the catalysts synthesized with chloride-free precursors are up to 50% greater than those synthesized with chloride precursors. The observed differences in catalytic activity were much less apparent above 450 °C. The observed activation energies of the catalysts were independent of the precursor utilized, despite the difference in catalytic activity, suggesting that the active site composition was the same for all catalysts. These results suggest a pathway to improved ammonia cracking catalysts by tailoring the precursor used in the synthesis.

## Introduction

**Figure 1:**
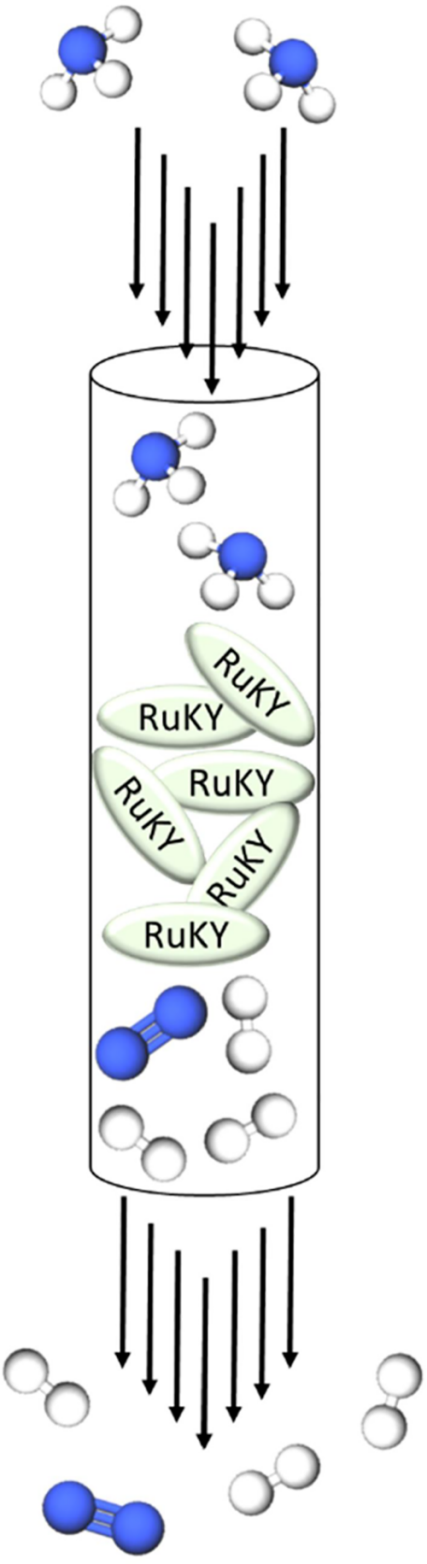
Diagram of ammonia cracking in a packed bed flow reactor.

Hydrogen is a promising zero-emission fuel that could replace fossil fuels as the world seeks alternative fuel sources to mitigate future greenhouse gas emissions.^1^ However, hydrogen fuel is expensive and difficult to transport safely.^2^ These issues can potentially be mitigated by storing hydrogen in the form of hydrogen-dense chemicals that are safer and easier to transport.^3^ Unfortunately, many hydrogen carriers are either too low in hydrogen content or produce CO_2_ gas when converted back to hydrogen.^4,5^ In contrast, ammonia has exceptionally high hydrogen density, is transported easily, and is carbon-free.^4^ However, while the synthesis of ammonia has undergone more than a century of optimization, the reverse process of decomposing ammonia into nitrogen and hydrogen, commonly referred to as “ammonia cracking” (Figure 1), has not been widely studied.^6^ One reason is that ammonia cracking typically requires extremely high temperatures (above 500 °C), whereas other promising hydrogen carriers like methanol or formic acid are typically decomposed below 200 °C.^7,8^ Thus, reducing the operational temperatures necessary for ammonia cracking is crucial to adopting ammonia as a viable hydrogen carrier for widespread use. In addition, lower temperatures required for ammonia cracking enable intensified separation processes that are precluded at high temperatures.^9,10^

For example, a potential intensified gas separation process could involve a separation membrane in the reactor. However, most gas separation membranes have relatively low operating temperatures. A common hydrogen separation membrane is palladium–silver (PdAg) foil, which is inoperable and irreversibly damaged above 500 °C.^11^ Most hydrogen carriers have an active temperature of decomposition well outside the operating temperatures of the PdAg membrane.^12^ However, carbon-based hydrogen carriers undergo a parasitic side reaction, a water–gas shift, at high temperatures, lowering the hydrogen recovered and limiting their applicability in a permeation membrane. Thus, ammonia cracking catalysts that can perform the reaction under permeation membrane operating conditions would provide a unique advantage of ammonia as a hydrogen carrier.

Ammonia decomposition catalysts have become an important field of research, and current work is on lowering the active temperature where decomposition occurs.^13^ Ruthenium, and to a lesser extent nickel and cobalt, have shown promise as active metals for decomposing ammonia to nitrogen and hydrogen.^14^ However, these active metals often have operating temperatures that are too high for industrial applications, and thus, additional promoters have been utilized to enhance their catalytic effects.^15^ Recent advancements in ammonia cracking catalyst design, guided by machine learning and high-throughput analysis, have yielded catalysts with operating temperatures below 450 °C.^8,16^ These catalysts are based on trimetallic ruthenium/alkali metal/promoter nanoparticles supported on metal oxides, which show a significant improvement over ruthenium/alkali metal nanoparticles.^17^ Further development of ammonia decomposition catalysts performed by Lauterbach and co-workers utilized the addition of a promoter, such as yttrium, and allowed catalysis in the temperature range compatible with the PdAg membrane.^8,9^ However, the intricacies of this catalyst synthesis have not been well explored, and as a consequence, the formation of the low-temperature active sites is not fully known. Thus, the operating temperatures of these novel catalysts may be further lowered through improvements in the catalyst synthesis methodology.

We report the observed differences between 3% ruthenium/12% potassium/1% yttrium (RuKY) catalysts on γ-alumina synthesized from chloride-, nitrate-, and acetate-based precursors. Catalysts synthesized from chloride precursors were significantly less conducive to ammonia cracking than those synthesized with nitrate precursors below 400 °C. For example, RuKY catalysts synthesized from chloride precursors displayed catalytic activities 50% lower than those synthesized from nitrate precursors at 350 °C. The turnover frequency (TOF) of the catalyst was also significantly lower for chloride precursor-based catalysts, resulting in TOFs of up to three times less than catalysts synthesized without chloride precursors. The differences in performance between these catalysts highlight the importance of the synthesis methodology, despite a similar final metal content.

## Results and Discussion

All low-temperature ammonia cracking catalysts were nominally 3 wt % Ru/1 wt % Y/12 wt % K on γ-Al_2_O_3_ pellets. All catalysts were synthesized via wet impregnation from metal salt solutions, and the initial dissolution of the metal precursor salts allowed for various ion-exchange reactions to potentially occur before deposition. For example, if both YCl and KOAc are present in the deposition mixture, then the exchange of the chloride and acetate anions can occur. This would allow for KCl to be formed in situ, which has been shown to interact with the alumina by reacting with terminal Al–O–H bonds.^18^ Various combinations of chloride, nitrate, and acetate precursors were used to synthesize the explored catalysts (Table 1). We denote catalysts synthesized from various precursors by referencing the anion in the order of the metal. For example, RuKY-Cl/OAc/Cl would be the catalyst synthesized from a solution of RuCl_3_, YCl_3_, and KOAc, whereas RuKY-NO_3_/NO_3_/NO_3_ would be synthesized from a solution of Ru(NO_3_)_3_NO, Y(NO_3_)_3_, and KNO_3_. All catalysts were calcined at 550 °C and activated under hydrogen at 450 °C. The color of the various catalysts depended on the precursor used (Figure S1), signifying that there was a conformational difference between the catalysts.

**Table 1:** Catalysts Tested and Their Precursors

catalyst name	ruthenium	potassium	yttrium
RuKY-Cl/OAc/Cl	RuCl_3_	KOAc	YCl_3_
RuKY-Cl/NO_3_/Cl	RuCl_3_	KNO_3_	YCl_3_
RuKY-NO_3_/OAc/NO_3_	Ru(NO_3_)_3_(NO)	KOAc	Y(NO_3_)_3_
RuKY-NO_3_/NO_3_/NO_3_	Ru(NO_3_)_3_(NO)	KNO_3_	Y(NO_3_)_3_

**Figure 2:**
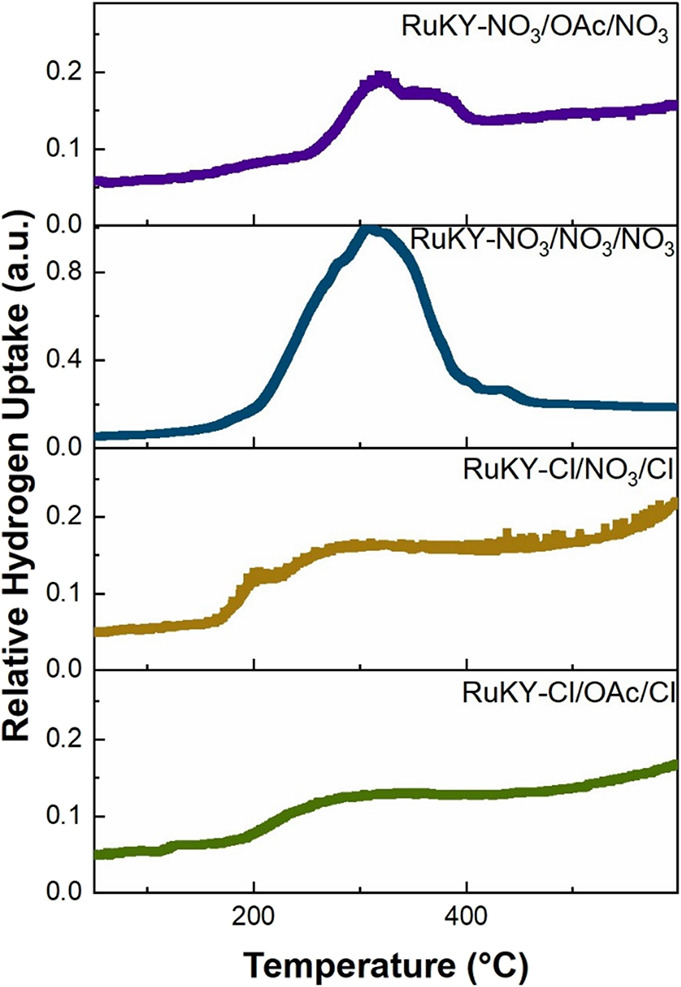
Temperature programmed reduction (TPR) of the four catalysts investigated.

The quantity and type of active sites of the catalyst can be related to the active temperature of reduction and the uptake of H_2_ by the catalyst. Thus, each catalyst was analyzed by temperature programmed reduction (TPR; Figure 2), in which the catalysts were reduced under H_2_. Among the catalysts tested, RuKY-NO_3_/NO_3_/NO_3_ absorbed the largest quantity of H_2_. This significant absorption of H_2_ indicates that the active sites in the RuKY-NO_3_/NO_3_/NO_3_ are activated at the reduction temperature of 450 °C. While all the catalysts absorb H_2_ at 300 °C, exhibiting similar reduction profiles, the H_2_ absorbed by the RuKY-NO_3_/NO_3_/NO_3_ catalyst is considerably greater than that of the other three catalysts, indicating that the formation of the low-temperature active site on the catalyst surface is dependent on the precursors utilized. The RuKY-NO_3_/OAc/NO_3_ catalyst absorbed significantly more H_2_ than both of the chloride-containing catalysts but significantly less than the all-nitrate catalyst. The RuKY-Cl/NO_3_/Cl and RuKY-Cl/OAc/Cl catalysts absorbed little H_2_ below 600 °C, indicating that the low-temperature active sites in these catalysts are less abundant due to the influence of the chloride anions. The relative area of H_2_ uptake of the RuKY-NO_3_/NO_3_/NO_3_ (722.12 mmol H_2_/g_cat_ uptake) catalyst to the RuKY-Cl/OAc/Cl (281.63 mmol H_2_/g_cat_ uptake) catalyst to the RuKY-Cl/NO_3_/Cl (361.06 mmol H_2_/g_cat_ uptake) catalyst to the RuKY-NO_3_/OAc/NO_3_ (324.95 mmol H_2_/g_cat_ uptake) catalyst is 1:0.39:0.5:0.45, respectively. These results suggest that the anions in the metal salts were impactful in the catalyst synthesis.^18^

We hypothesize that the chloride anions in the precursors interact with the metals, preventing a synergistic effect with γ-Al_2_O_3_ or the other metals. The solubility of the various metal-anion mixtures may be influencing the deposited metal species after wet impregnation. For example, the most soluble salt in the RuKY-Cl/OAc/Cl system is KCl, which may be the dominant form of potassium deposited, despite not being initially present (Table S1). KCl has previously been reported to inhibit catalytic activity due to the incorporation of KCl into alumina.^18^ This can disrupt the synergistic effects that the alumina has with the RuKY catalyst and lower the activity of chloride-based catalysts. However, these chloride species were not present in the RuKY-NO_3_/NO_3_/NO_3_ and RuKY-NO_3_/OAc/NO_3_ catalyst synthesis, yet a difference in the H_2_-TPR is still present. RuKY-NO_3_/OAc/NO_3_ can exchange anions between RuNO(NO_3_)_3_ and KOAc, which may have led to the difference in activity between RuKY-NO_3_/OAc/NO_3_ and RuKY-NO_3_/NO_3_/NO_3_ shown in the H_2_-TPR.

**Figure 3:**
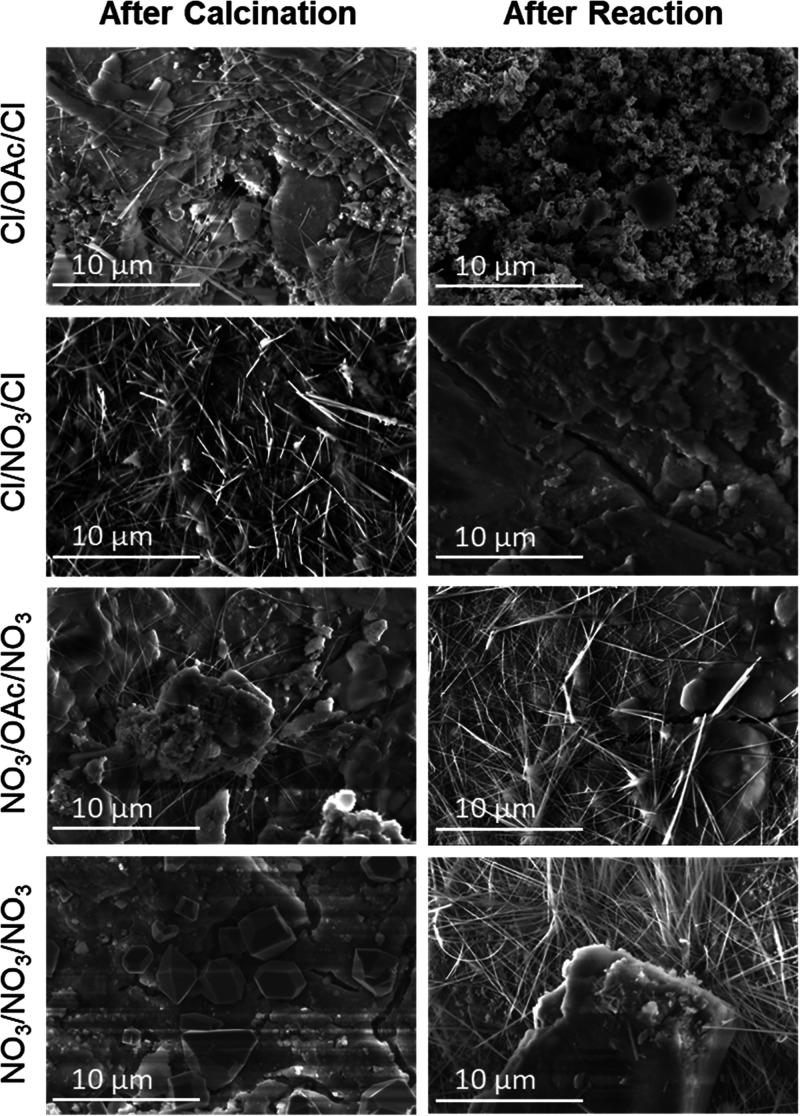
SEM micrographs of the RuKY-Cl/OAc/Cl, RuKY-Cl/NO_3_/Cl, RuKY-NO_3_/Oac/NO_3_, and RuKY-NO_3_/NO_3_/NO_3_ catalysts before and after the reaction with NH_3_.

To determine whether structural changes of the catalyst were occurring during the reaction, scanning electron microscopy (SEM) and electron dispersive X-ray (EDX) spectroscopy were performed. The catalysts with chloride precursors (Figure 3) had wire-like structures that are indicative of the formation of a hollandite, KRu_4_O_8_.^8,15^ However, after the reaction, these wire-like structures dissipated from the catalyst, indicating a morphology change of the catalyst over the course of the reaction. The catalysts synthesized from acetate and nitrate precursors did not exhibit this hollandite structure and did not undergo the hollandite morphology change. This correlates to the WAXS-XRD diffraction patterns shown in Figures S3–S6 and Tables S2–S9, where the catalyst synthesized with chloride precursors had different patterns before and after the reaction, relating to the hollandite formation in the chloride-based catalysts and ruthenium nanoparticles in the nitrate-based catalysts. However, an interesting note is that the catalysts synthesized with nitrate precursors had string-like structures in the SEM for the catalyst after the reaction. These new structures indicate an increase in surface area and may be a reason as to why the nitrate-based precursors perform better than the chloride-based precursors at lower temperatures. EDX spectra are shown in Figures S7–S40 for the catalysts and indicate that the metal was well dispersed throughout the catalyst. Images were collected of both the outer shell of the alumina pellets and the cross-section of the pellets, and the ruthenium metal was primarily on the outer shell of all four catalysts with small amounts of ruthenium in the center cross-section.

**Figure 4:**
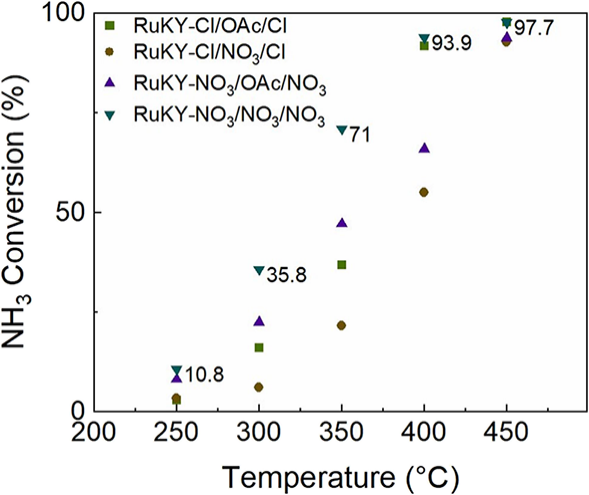
Performance of the RuKY catalysts when synthesized from different precursors at varying temperatures (conditions of 6000 mL·g_cat_^–1^·h^–1^ 10%NH_3_ in Ar).

The ammonia cracking efficacy of 3%Ru/12%K/1%Y/γ-Al_2_O_3_ catalysts was tested in a packed bed reactor, and the conversion as a function of the temperature is shown in Figure 4. Below 350 °C, RuKY-NO_3_/NO_3_/NO_3_ catalysts displayed the highest activity, followed by RuKY-NO_3_/OAc/NO_3_, RuKY-Cl/OAc/Cl, and RuKY-Cl/OAc/Cl, respectively. This activity trend is analogous to the H_2_ absorption trend discussed earlier. The RuKY-NO_3_/NO_3_/NO_3_ catalyst achieved a 71% conversion at 350 °C (6000 mL·h^–1^·g_cat_^–1^ 10% NH_3_ in Ar). However, above 400 °C, the difference in activity was less pronounced, and the activity of the RuKY-Cl/OAc/Cl catalyst was nearly equivalent to that of RuKY-NO_3_/OAc/NO_3_. Both catalysts achieved almost complete conversion at 450 °C, with 97.8 and 97.7% NH_3_ conversion achieved by RuKY-Cl/OAc/Cl and RuKY-NO_3_/NO_3_/NO_3_, respectively, under a 10%NH_3_ feedstock. Yet as the amount of ammonia in the feedstock gas was increased, the difference in reactivity between the two catalysts became pronounced (Figure S41). Under a pure ammonia stream, there was a 15% difference in reactivity between the RuKY-Cl/OAc/Cl and RuKY-NO_3_/NO_3_/NO_3_ catalysts at 450 °C. The catalytic activity differences were unexpected, as the sole difference between the catalysts was the precursor anions, which should be almost fully removed via calcination and reduction. While the RuKY-Cl/NO_3_/Cl catalyst is the worst performing catalyst, most likely due to the rapid exchange of the precursor anions in the synthesis of the catalyst, the RuKY-NO_3_/OAc/NO_3_ performs better than the RuKY-Cl/OAc/Cl below 400 °C and worse above 400 °C. This may be attributable to the difference in crystallite size between the catalysts.

Additionally, NH_3_ temperature programmed desorption (TPD) was performed to better understand the interaction between the catalyst and substrate (Figures S42–S45). The adsorption was performed at room temperature, and all the catalysts adsorbed ammonia, with the catalysts synthesized from chloride-free precursors adsorbing more ammonia than the catalysts synthesized from chloride precursors. The desorption of ammonia happened in two stages: an initial release of physisorbed ammonia and the release of chemisorbed ammonia at higher temperatures (between 400 and 450 °C). The RuKY-Cl/NO_3_/Cl and RuKY-Cl/OAc/Cl both had less chemisorbed ammonia than the RuKY-NO_3_/NO_3_/NO_3_ and RuKY-NO_3_/OAc/NO_3_ catalysts. This would mean that at lower temperatures, there is a difference in ammonia that can adsorb at the temperatures tested on each of the four catalysts tested depending on the precursors utilized. The difference in adsorption leads to less ammonia on the surface of the catalyst for the dehydrogenation reaction. Therefore, it is likely that the lower conversion rates (Figure 4) of the chloride-based catalysts are partly attributable to the difference in surface ammonia adsorption below 450 °C.

**Figure 5:**
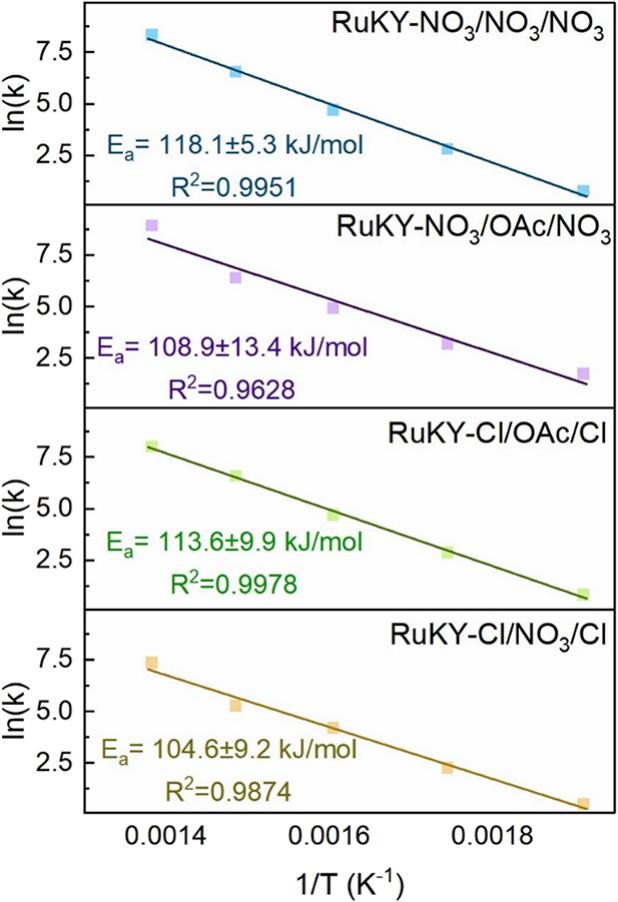
Arrhenius plots of the four catalysts tested ranging from 250 to 450 °C (conditions (100% NH_3_ flow varying flows from 2400 to 9600 mL·g_cat_^–1^·h^–1^).

The differences in catalytic activity can be further elucidated through an analysis of the reaction activation energies and TOFs. Arrhenius plots and the calculated reaction activation energy (assuming a second-order plug flow reactor) for low-temperature ammonia cracking are shown in Figure 5. The reaction being second order is indicative of the rate-limiting step being the recombination of the two nitrogen atoms (commonly termed the N–N recombination step). The four tested catalysts displayed similar activation energies (104.6–118.1 kJ/mol), despite the large differences in measured catalytic activity. The similar activation energies of the catalysts suggest that similar active sites are present in all four catalysts. However, in contrast to the activation energies, the TOF of the tested catalysts varied significantly at low temperatures (Table 2). Following the general trend in reactivity, the RuKY-NO_3_/NO_3_/NO_3_ catalyst exhibited the highest TOF (2.41 s^–1^) at 400 °C while the RuKY-NO_3_/Cl/NO_3_ catalyst exhibited the lowest TOF (1.41 s^–1^). This trend holds from 400 to 300 °C, indicating that the low-temperature reactivity is suppressed by the chloride precursor. This trend corresponds well with the TPR results discussed previously, where the catalysts synthesized without chloride were the more active catalysts at lower temperatures. We hypothesize that the chloride precursors during the process of calcination are interacting with the metals and the alumina, which is partially inhibiting the formation of the low-temperature active site that is present in the catalysts synthesized without chloride precursors. This allows for similar activation energies to be shared across the four catalysts but results in differing TOFs.

**Table 2:** Activation Energy and Turnover Frequency (TOF) of the Four Catalysts Tested

		TOF (s^–1^)		
catalyst	*E*_a_ (kJ/mol)	400 °C	350 °C	300 °C
RuKY-Cl/OAc/Cl	113.6 ± 9.9	2.35	0.95	0.41
RuKY-NO_3_/Cl/NO_3_	104.6 ± 9.2	1.41	0.55	0.16
RuKY-NO_3_/OAc/NO_3_	108.9 ± 13.4	1.69	1.21	0.58
RuKY-NO_3_/NO_3_/NO_3_	118.1 ± 5.3	2.41	1.82	0.92

The RuKY-NO_3_/NO_3_/NO_3_ showcases improvements from previously reported ammonia cracking catalysts in that it performs far better than other low-temperature catalysts. For example, a 1%Ru/3%Sr/12%K/Al2O3 catalyst was shown to have good reactivity at low temperatures and was able to achieve a TOF of 1.78 s^–1^ at 400 °C.^8^ This is lower than that of the RuKY-NO_3_/NO_3_/NO_3_ catalyst that achieved a TOF of 2.41 s^–1^ at the same temperature. Additionally, the activation energy was reported to be 156.4 ± 1.6 kJ/mol for the 1%Ru/3%Sr/12%K/Al2O3 catalysts, which is significantly higher than the 118.1 ± 5.3 kJ/mol that is reported in this report. Other catalysts, such as 7%Ru/CeO_2_ and 4.8%Ru/La_2_O_3_, were previously shown to have TOFs of 0.034 and 0.43 s^–1^ at 350 °C, respectively.^19^ Both of those TOFs are lower than that of the RuKY-NO_3_/NO_3_/NO_3_ catalyst at 350 °C, 1.82 s^–1^. However, for the above examples, the precursors utilized did not matter in terms of better performance. However, other high-performing, trimetallic, ammonia cracking catalysts, like 4.6%Ru/4.6%Ba/MgAl_2_O_4_, performed similarly to the RuKY-NO_3_/OAc/NO_3_ catalyst at 400 °C and achieved a TOF of 1.64 s^–1^ at 400 °C compared to 1.69 s^–1^ of the RuKY-NO_3_/OAc/NO_3_.^20^ However, the RuKY-NO_3_/NO_3_/NO_3_ catalyst has a higher TOF at 400 °C (2.41 s^–1^) and indicates the need for fine-tuning of the precursors. Overall, the work presented for the improvements to ruthenium-based catalysts showcases significant improvement in low-temperature conversion, a necessity for industrial viability. This is due to NOx being generated if ammonia is not fully cracked and has been shown that ammonia conversion rates below 99.6% lead to equal or greater greenhouse gas emissions to fossil fuels.^21^ The work presented here allows for greater advancements and feasibility to reach that threshold but would require permeation membrane reactors and other engineering considerations to reach that high of conversion.

## Conclusions

The efficacy of trimetallic ammonia cracking catalysts is dependent on the metal precursors used in the synthesis of the catalysts. Chloride precursors produced less efficient catalysts than nitrate precursors, which may be due to the interaction of chloride anions with the support and metals during calcination. This is further supported by the TPR results, where the catalyst synthesized without chloride precursors had a much greater adsorption of H_2_ at lower temperatures. However, the activation energies of the catalysts are roughly equivalent, indicating that the active sites are not dependent on the precursor anion. As ammonia cracking reactions are not typically performed at lower temperatures, these differences are usually negligible. For future utility in the hydrogen economy, these differences in low-temperature reactions will play a greater role. The methodology to enhance the efficacy of ammonia-cracking catalysts will allow for further optimization of catalysts beyond that of the halogen content of the precursor.

## Experimental Section

### Catalyst Synthesis

Ruthenium(III) chloride (RuCl_3_, Sigma-Aldrich #208523, lot# MKCM4092), ruthenium(III) nitrosyl nitrate (Ru(NO_3_)_3_NO, ThermoFisher Scientific no. 012175.06, lot no. Z20J027), yttrium(III) chloride (YCl_3_, Sigma-Aldrich, 99.99%), yttrium(III) nitrate hexahydrate (Y(NO_3_)_3_·6H_2_O, ThermoFisher Scientific, 99.9%), potassium acetate (KOAc, Sigma-Aldrich, 99%), and potassium nitrate (KNO_3_, ThermoFisher Scientific, 99%) were utilized as precursors for the catalyst. Four catalysts were synthesized utilizing different combinations of precursors, and the final weight percentages were calculated to be 3%Ru/1%Y/12%K/Al_2_O_3_. The metal precursors were then dissolved in 4 mL of deionized (DI) H_2_O and deposited dropwise on gamma-alumina pellets (γ-Al_2_O_3_, Sasol, lot no. TK2347, pellet size between 1 and 2 mm) with constant stirring. After 4 mL of metal precursor solution was added, an additional 1 mL of DI H_2_O was used to ensure maximum transfer. The pellets were then dried overnight in an oven at 80 °C. Then the catalysts were calcined at 550 °C for 2 h in air with a ramp rate of 20 °C/min and stored for further use. The catalyst synthesis was done on a scale of five grams.

### Ammonia Decomposition Reaction

0.5 g of catalyst was measured and placed into a stainless-steel reactor, where it was heated to 450 °C under Ar (50 mL/min). The catalyst was then reduced with 10 mL/min of H_2_ and 40 mL/min of Ar. The reduction conditions were maintained for 3 h. After reduction, H_2_ was purged with Ar. NH_3_ (Sigma-Aldrich) was then introduced into the system with 50 mL/min Ar. Flow rates were varied at each temperature tested (250, 300, 350, 400, and 450 °C), and the system was purged with Ar in between each temperature tested. The conversion results were calculated via mass spectroscopy (MKS Cirrus 2 Mass Spectrometer).

### Conversion Calculation

**Scheme 1:**
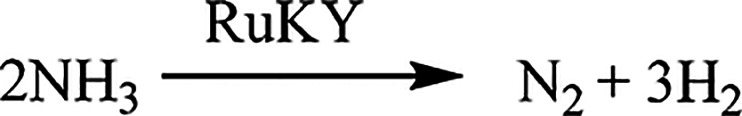
Ammonia Cracking Reaction

Scheme 1 shows the conversion of ammonia into nitrogen and hydrogen, which is utilized for the conversion calculations and for the rate equations.
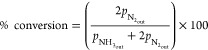
1

Eq 1, conversion rate calculation.

The conversion rate was calculated from the pressure registered from the RGA signals (a representative example is shown in Figure S46). Then the pressure of nitrogen was divided by the summation of ammonia and nitrogen and multiplied by 100 for the conversion rate. The pressures were adjusted for the stoichiometric conversion of ammonia, and thus, the nitrogen signal was multiplied by 2. The TOF was calculated based on the total metal deposited on the catalyst.

### Calculation of Activation Energy

The activation energies were calculated based on a plug flow model. The equation shows the *y*-term of the second-order reaction plots, and the x-term was τC_Ao_ in mol·s·L^–1^.
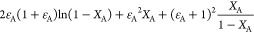
2where ε_A_ = change in moles of gas; *X*_A_ = conversion rate

Eq 2, second order *y*-term for second order reaction plots.

From Scheme 1, the change in moles in gas over the course of the reaction is 2 and is utilized for ε_A_. The conversion rates were used for the *X*_A_ values, and the *k* values were calculated from the slope of the second-order plug flow plot.
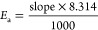
3

Eq 3, activation energy calculated from the slope of the Arrhenius plot in kJ/mol.

From the rate constants that were calculated, the Arrhenius plots were constructed utilizing ln(*k*) as the *y*-term and temperature as the *x*-term (1/*T* = K^–1^). The slope of the Arrhenius plot was then utilized to calculate the activation energy, as shown in eq 3. An example of calculating the activation energy is shown in Figure S47.

### SEM and EDX Spectroscopy

The SEM and EDX were obtained on a Hitachi SU8200. The voltage was set to 20 kV, and the spot size was set to 10 nm.

### TPR

The catalysts were tested under a hydrogen flow from 50 to 600 °C. One g portion of each sample was measured into a sample tube. The samples were heated to 600 °C at a rate of 10 °C/min, and the effluent was analyzed by an MKS Cirrus 3 mass spectrometer.

### NH_3_ TPD

The catalysts were first activated through a TPR (same conditions as above) to ensure that the catalyst was in an active form. Then a 5%NH_3_ in argon was introduced to the catalyst at 30 mL/min. The adsorption step was held for 30 min to ensure full ammonia adsorption. Then the catalyst, under an argon atmosphere, was heated from 35 to 450 °C at a rate of 10 °C/min. 0.4 g of each sample was measured into a sample tube. The effluent was analyzed with an MKS Cirrus 3 mass spectrometer.

## Supporting Information Available

Images of crushed catalysts; table of solubility of the precursors utilized; WAXS and XRD; SEM and EDX; additional data on higher flow rates; ammonia temperature programmed desorption; example mass spectrometry spectra; and more detailed explanation of the calculation of the activation energy (PDF)

## References

[ref1] Armor J. N. Catalysis and the hydrogen economy. Catal. Lett. 2005, 101 (3), 131–135. 10.1007/s10562-005-4877-3.

[ref2] McDowall W.; Eames M. Forecasts, scenarios, visions, backcasts and roadmaps to the hydrogen economy: A review of the hydrogen futures literature. Energy Policy 2006, 34 (11), 1236–1250. 10.1016/j.enpol.2005.12.006.

[ref3] Rao P. C.; Yoon M. Potential Liquid-Organic Hydrogen Carrier (LOHC) Systems A Review on Recent Progress. Energies 2020, 13 (22), 6040 10.3390/en13226040.

[ref4] He T.; Pachfule P.; Wu H.; Xu Q.; Chen P. Hydrogen carriers. Nat. Rev. Mater. 2016, 1 (12), 16059 10.1038/natrevmats.2016.59.

[ref5] Kothandaraman J.; Kar S.; Sen R.; Goeppert A.; Olah G. A.; Prakash G. K. S. Efficient Reversible Hydrogen Carrier System Based on Amine Reforming of Methanol. J. Am. Chem. Soc. 2017, 139 (7), 2549–2552. 10.1021/jacs.6b11637. 28151661

[ref6] Klerke A.; Christensen C. H.; Nørskov J. K.; Vegge T. Ammonia for hydrogen storage challenges and opportunities. J Mater Chem 2008, 18 (20), 2304–2310. 10.1039/B720020J.

[ref7] Jayarathna R.; Onsree T.; Naglic J.; Lauterbach J. Experimental discovery of novel ammonia synthesis catalysts via active learning. J. Mater. Chem. A 2024, 12 (5), 3046–3060. 10.1039/d3ta05939a.

[ref8] McCullough K.; Chiang P. H.; Jimenez J. D.; Lauterbach J. A. Material Discovery and High Throughput Exploration of Ru Based Catalysts for Low Temperature Ammonia Decomposition. Materials 2020, 13 (8), 1869 10.3390/ma13081869. 32316302 PMC7215519

[ref9] Jiang J.; Dong Q.; McCullough K.; Lauterbach J.; Li S.; Yu M. Novel hollow fiber membrane reactor for high purity H2 generation from thermal catalytic NH3 decomposition. J. Membr. Sci. 2021, 629, 119281 10.1016/j.memsci.2021.119281.

[ref10] Uemiya S.; Matsuda T.; Kikuchi E. Hydrogen permeable palladium-silver alloy membrane supported on porous ceramics. J. Membr. Sci. 1991, 56 (3), 315–325. 10.1016/S0376-7388(00)83041-0.

[ref11] Allison E. G.; Bond G. C. The Structure and Catalytic Properties of Palladium-Silver and Palladium-Gold Alloys. Catal. Rev. 1972, 7 (2), 233–289. 10.1080/01614947208062259.

[ref12] Okazaki J.; Ikeda T.; Tanaka D. A. P.; Sato K.; Suzuki T. M.; Mizukami F. An investigation of thermal stability of thin palladium–silver alloy membranes for high temperature hydrogen separation. J. Membr. Sci. 2011, 366 (1), 212–219. 10.1016/j.memsci.2010.10.011.

[ref13] Lucentini I.; Garcia X.; Vendrell X.; Llorca J. Review of the Decomposition of Ammonia to Generate Hydrogen. Ind. Eng. Chem. Res. 2021, 60 (51), 18560–18611. 10.1021/acs.iecr.1c00843.

[ref14] Huang Y.; Ren H.; Fang H.; Ouyang D.; Chen C.; Luo Y.; Lin L.; Wang D.; Jiang L. Ru nanoparticles embedded in Ru/SiO2@N-CS for boosting hydrogen production via ammonia decomposition with robust lifespan. Appl. Surf. Sci. 2024, 669, 160517 10.1016/j.apsusc.2024.160517.

[ref15] Pyrz W.; Vijay R.; Binz J.; Lauterbach J.; Buttrey D. J. Characterization of K-Promoted Ru Catalysts for Ammonia Decomposition Discovered Using High-Throughput Experimentation. Top. Catal. 2008, 50 (1), 180–191. 10.1007/s11244-008-9095-y.

[ref16] McCullough K.; Williams T.; Mingle K.; Jamshidi P.; Lauterbach J. High-throughput experimentation meets artificial intelligence: A new pathway to catalyst discovery. Phys. Chem. Che. Phys. 2020, 22 (20), 11174–11196. 10.1039/D0CP00972E. 32393932

[ref17] Zhang Z.; Liguori S.; Fuerst T. F.; Way J. D.; Wolden C. A. Efficient Ammonia Decomposition in a Catalytic Membrane Reactor To Enable Hydrogen Storage and Utilization. ACS Sustain. Chem. Eng. 2019, 7 (6), 5975–5985. 10.1021/acssuschemeng.8b06065.

[ref18] Li D.; Ichikuni N.; Shimazu S.; Uematsu T. Catalytic properties of sprayed Ru/Al2O3 and promoter effects of alkali metals in CO_2_ hydrogenation. Appl. Catl. A. Gen. 1998, 172 (2), 351–358. 10.1016/S0926-860X(98)00139-2.

[ref19] Huang C.; Yu Y.; Yang J.; Yan Y.; Wang D.; Hu F.; Wang X.; Zhang R.; Feng G. Ru/La_2_O_3_ catalyst for ammonia decomposition to hydrogen. Appl. Surf. Sci. 2019, 476, 928–936. 10.1016/j.apsusc.2019.01.112.

[ref20] Szmigiel D.; Raróg-Pilecka W.; Miśkiewicz E.; Kaszkur Z.; Kowalczyk Z. Ammonia decomposition over the ruthenium catalysts deposited on magnesium–aluminum spinel. Appl. Catl. A. Gen. 2004, 264 (1), 59–63. 10.1016/j.apcata.2003.12.038.

[ref21] Bertagni M. B.; Socolow R. H.; Martirez J. M. P.; Carter E. A.; Greig C.; Ju Y.; Lieuwen T.; Mueller M. E.; Sundaresan S.; Wang R.; et al. Minimizing the impacts of the ammonia economy on the nitrogen cycle and climate. Proc. Natl. Acad. Sci. U. S. A. 2023, 120 (46), e2311728120 10.1073/pnas.2311728120. 37931102 PMC10655559

