# Supplementary material for: Effect of Precursors on Trimetallic Ruthenium-Based Catalysts Supported on γ-Al2O3 Pellets for Low-Temperature Ammonia Decomposition
Source: ACS Omega. 2025 Apr 10;10(15):15243–9. doi: 10.1021/acsomega.4c09968 (PMC12019494; doi:10.1021/acsomega.4c09968)
Supplement: Supplementary file 1 — ao4c09968_si_001.pdf [file ao4c09968_si_001.pdf]

Effect of precursors on trimetallic ruthenium-based catalysts supported on γ-Al_2O_3 pellets for low-temperature ammonia decomposition

Christopher J. Koch[†], Jennifer Naglic[†], Logan Kearney[ψ], Daniel Clairmonte[†], Binod Rai[†], Jochen Lauterbach[+], Lucas M. Angelette[†], Tyler Guin[†,*]

[†] Savannah River National Laboratory, Hydrogen Isotope Processing Science, Aiken, South Carolina 29803

[+] Department of Chemical Engineering, University of South Carolina, 541 Main St., Columbia, SC 29208, USA

[ψ] Chemical Sciences Division, Oak Ridge National Laboratory, 1 Bethel Valley Road, Oak Ridge, 37831, TN, United States

[*]Corresponding author: tylercguin@gmail.com

Contents

Images of crushed Pellets 2
Table of Solubility 4
WAX and XRD 5
Scanning Electron Microscopy (SEM) and Electron Dispersion Spectroscopy (EDX) 13
Additional Data 47
Ammonia Temperature Programmed Desorption (TPD)..48
Mass Spectrometry..52
Activation Energy 53

Images of crushed pellets

The pellets were crushed for WAXS and XRD measurements and were photographed. These crushed pellets show a varied appearance based on the precursors utilized. Figure S1 shows the crushed pellets after calcination but before reaction. **Error! Reference source not found.** shows the crushed pellets after reaction.

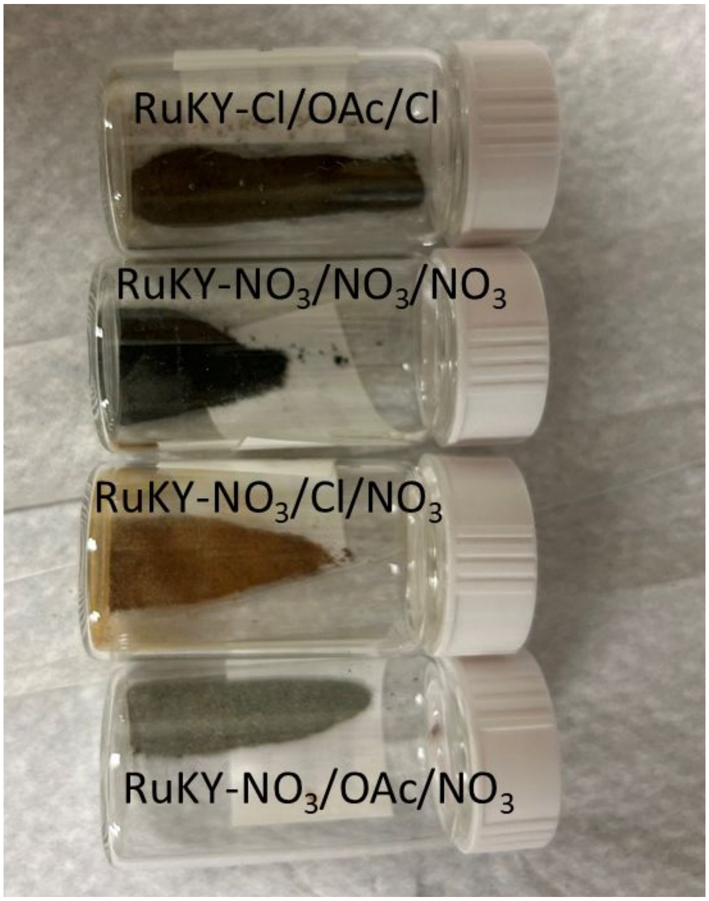

Figure S1. Calcined Pellets crushed into powders for analysis.

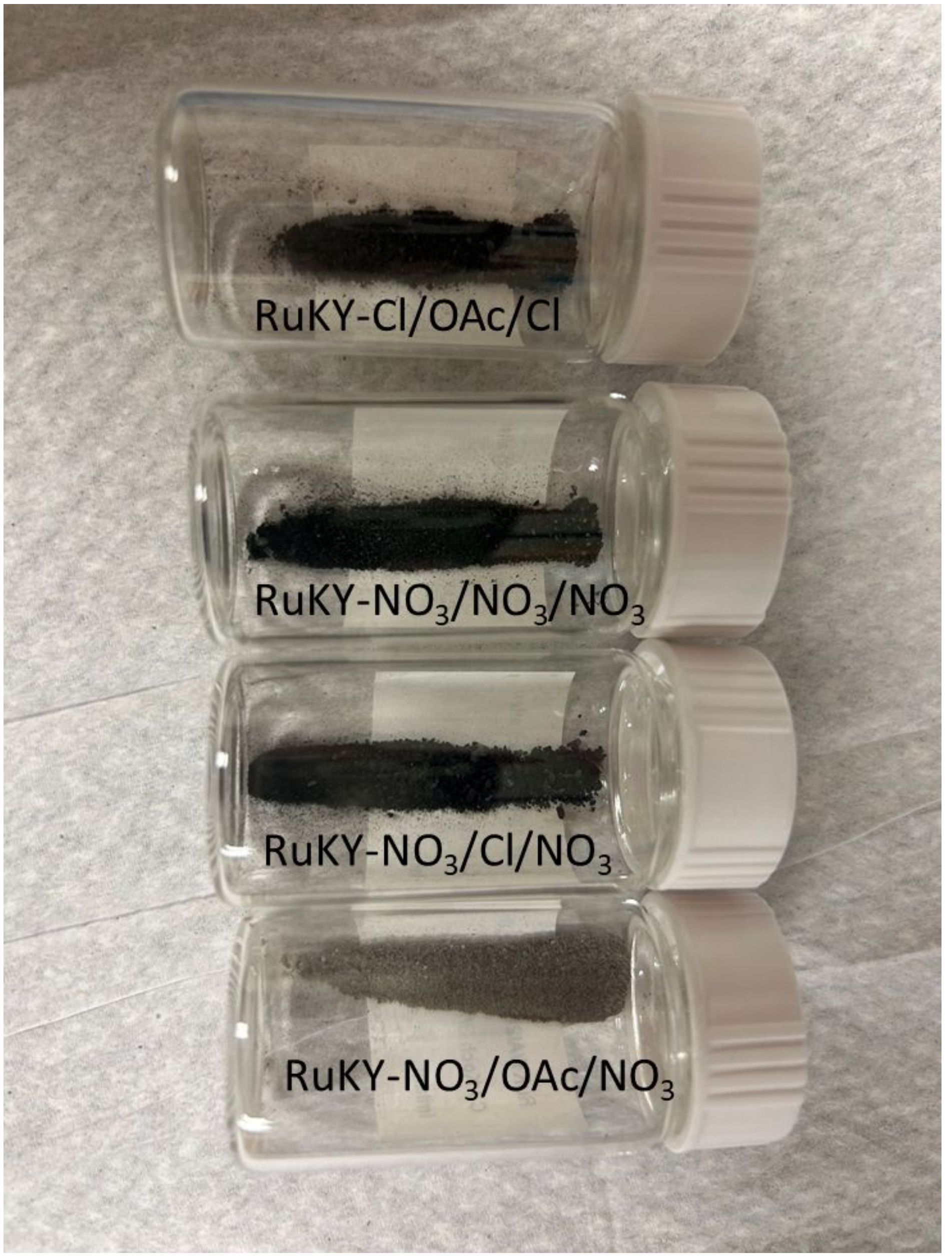

Figure S2. Pellets after reaction crushed into powders for analysis.

Table of Solubility

Table S1 showcases the solubility of the salts in water utilized in the synthesis of the catalyst. The differences in solubility help to explain some of the discrepancies of reactivity in the catalysts as KCl is the most soluble salt and would thus be formed in situ. Thus, utilizing other salts that preclude chloride from being in the catalyst synthesis would be beneficial.

Table S1. Solubility of different metal salts.

Compound	Solubility (g/mL)
$RuCl_3$	3.1
$Ru(NO_3)_3NO$	1.1
YCl_3	0.8
$Y(NO_3)_3$	2.3
KNO_3	3.1
KCl	3.4
KOAc	2.6

WAX and XRD

The following spectra were collected on a Wide-Angle X-Ray Scattering (WAXS) Spectrometer and the values were converted to 2Θ following Bragg's equation.

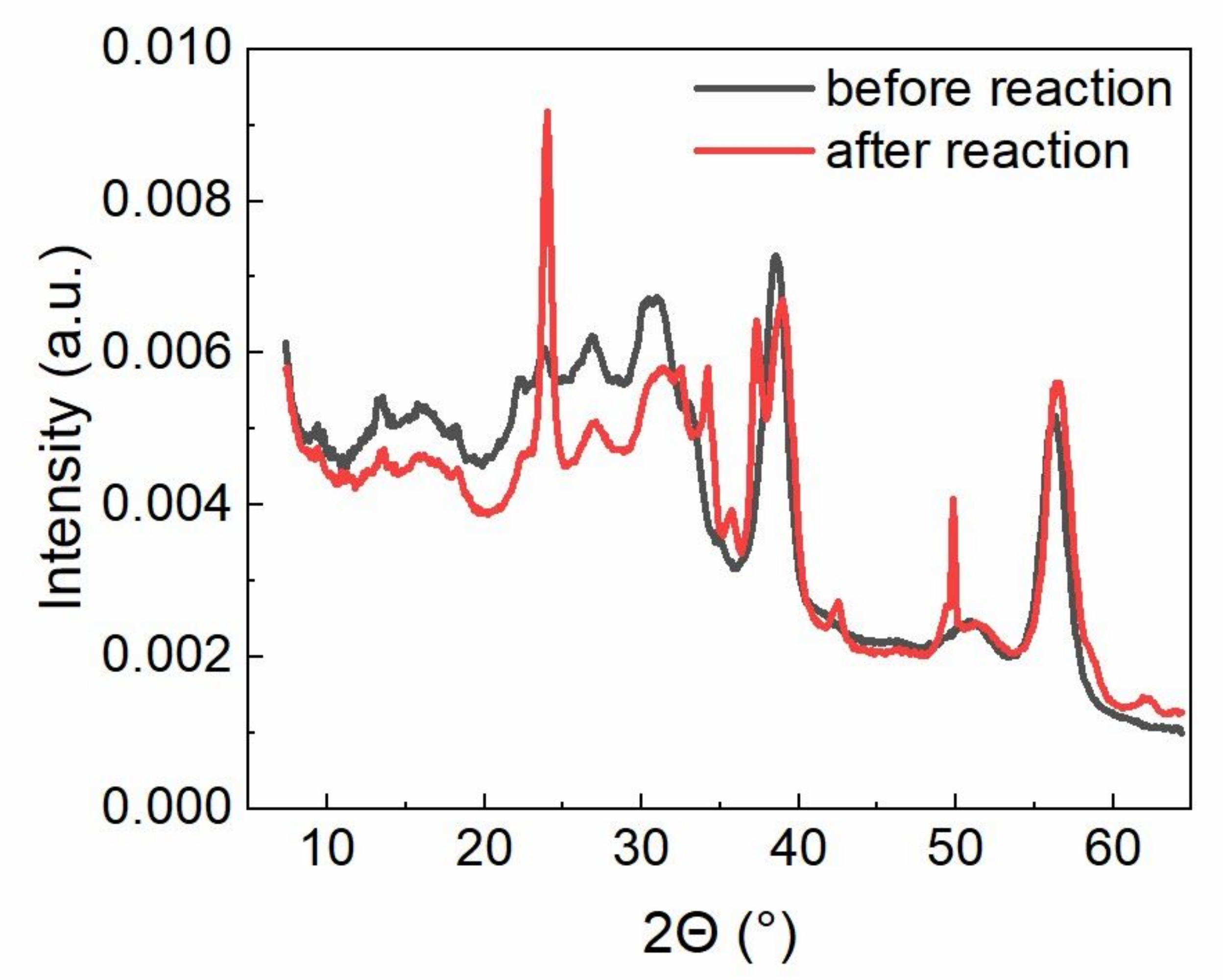

Figure S3. The WAXS spectra of the RuKY-NO_3/Cl/NO_3 catalyst before (shown in gray) and after (shown in red) the reaction.

Table S2. Analysis of the WAX pattern of the RuKY-Cl/NO_3/Cl catalyst before reaction.

**2Θ (°)**	**D spacing (Å)**	**Phase (hkl)**
23.8	3.73	K_2RuO_4 (2,0,0)
26.8	3.32	Y_2O_3 (2,0,2)
30.9	2.89	K_2O (1,1,0)
33.2	2.70	Y_2O_3 (-1,1,2), γ-Al_2O_3 (3,1,1)
38.6	2.33	K_2RuO_4 (2,2,1), γ-Al_2O_3 (2,2,2)
50.8	1.80	K_2RuO_4 (4,0,2)

56.3	1.63	Y_2O_3 (6,0,3), γ-Al_2O_3 (4,2,2)

Table S3. Analysis of the WAX pattern of the RuKY-Cl/NO_3/Cl catalyst after reaction.

2Θ (°)	d-spacing (Å)	Phase (hkl)
27.0	3.30	K_3AlO_3 (-2,0,1)
30.9	2.89	K_3AlO_3 (2,2,0)
34.2	2.62	Y_2O_3 (4,0,0)
35.7	2.51	Y_2O_3 (4,1,1)
37.3	2.41	γ-Al_2O_3 (3,1,1)
39.0	2.31	Ru (1,0,0)
42.5	2.13	Ru (0,0,2)
49.8	1.83	K_3AlO_3 (0,6,0)
51.3	1.78	K_3AlO_3 (4,2,0)
56.5	1.63	γ-Al_2O_3 (4,2,2)

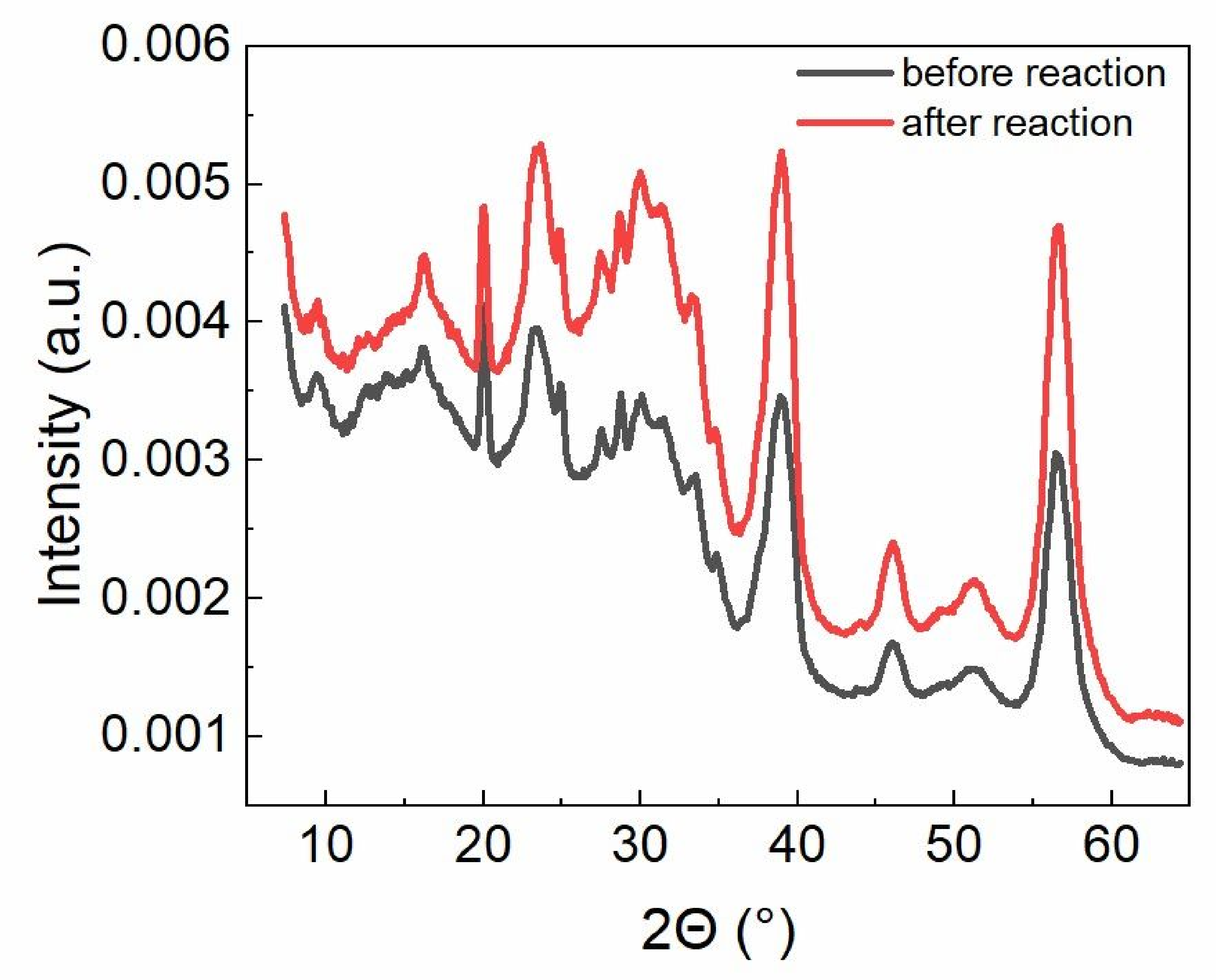

Figure S4. The WAXS spectra of the RuKY-NO_3/NO_3/NO_3 catalyst before (shown in gray) and after (shown in red) the reaction.

Table S4. Analysis of the WAX pattern of the RuKY-NO_3/NO_3/NO_3 catalyst before reaction.

2Θ (°)	d-spacing (Å)	Phase (hkl)
16.1	5.50	K_3AlO_3 (0,2,0)
20.1	4.42	γ-Al_2O_3 (1,1,1)
23.4	3.80	K_3AlO_3 (-1,1,1)
24.9	3.58	γ-Al_2O_3 (0,1,2)
27.6	3.23	RuO_2 (1,1,0), K_3AlO_3 (1,3,0)
28.7	3.11	K_3AlO_3 (0,0,2)
30.1	2.97	K_3AlO_3 (-1,1,2)
31.5	2.84	γ-Al_2O_3 (2,2,0)
33.4	2.68	K_3AlO_3 (1,0,7)
34.8	2.57	RuO_2 (1,0,1)
38.9	2.31	K_3AlO_3 (-3,1,1)
46.1	1.97	γ-Al_2O_3 (4,0,0)
51.3	1.78	Y_2O_3 (4,4,0)
56.5	1.63	γ-Al_2O_3 (4,2,2)

Table S5. Analysis of the WAX pattern of the RuKY-NO_3/NO_3/NO_3 catalyst after reaction.

2Θ (°)	d-spacing (Å)	Phase (hkl)
16.3	5.44	K_3AlO_3 (0,2,0)
20.0	4.43	γ-Al_2O_3 (1,1,1)
23.6	3.76	K_3AlO_3 (-1,1,1)
24.8	3.59	γ-Al_2O_3 (0,1,2)
27.5	3.24	K_3AlO_3 (1,3,0)
28.7	3.11	K_3AlO_3 (0,0,2)
29.9	2.99	K_3AlO_3 (-1,1,2)
31.3	2.85	γ-Al_2O_3 (2,2,0)
33.4	2.85	K_3AlO_3 (1,0,7)
34.9	2.57	YRu_2 (1,1,0)
39.0	2.31	K_3AlO_3 (-3,1,1), YRu_2 (2,0,0)
46.1	1.97	γ-Al_2O_3 (4,0,0)
51.3	1.78	YRu_2 (2,0,3)
56.6	1.62	γ-Al_2O_3 (4,2,2)

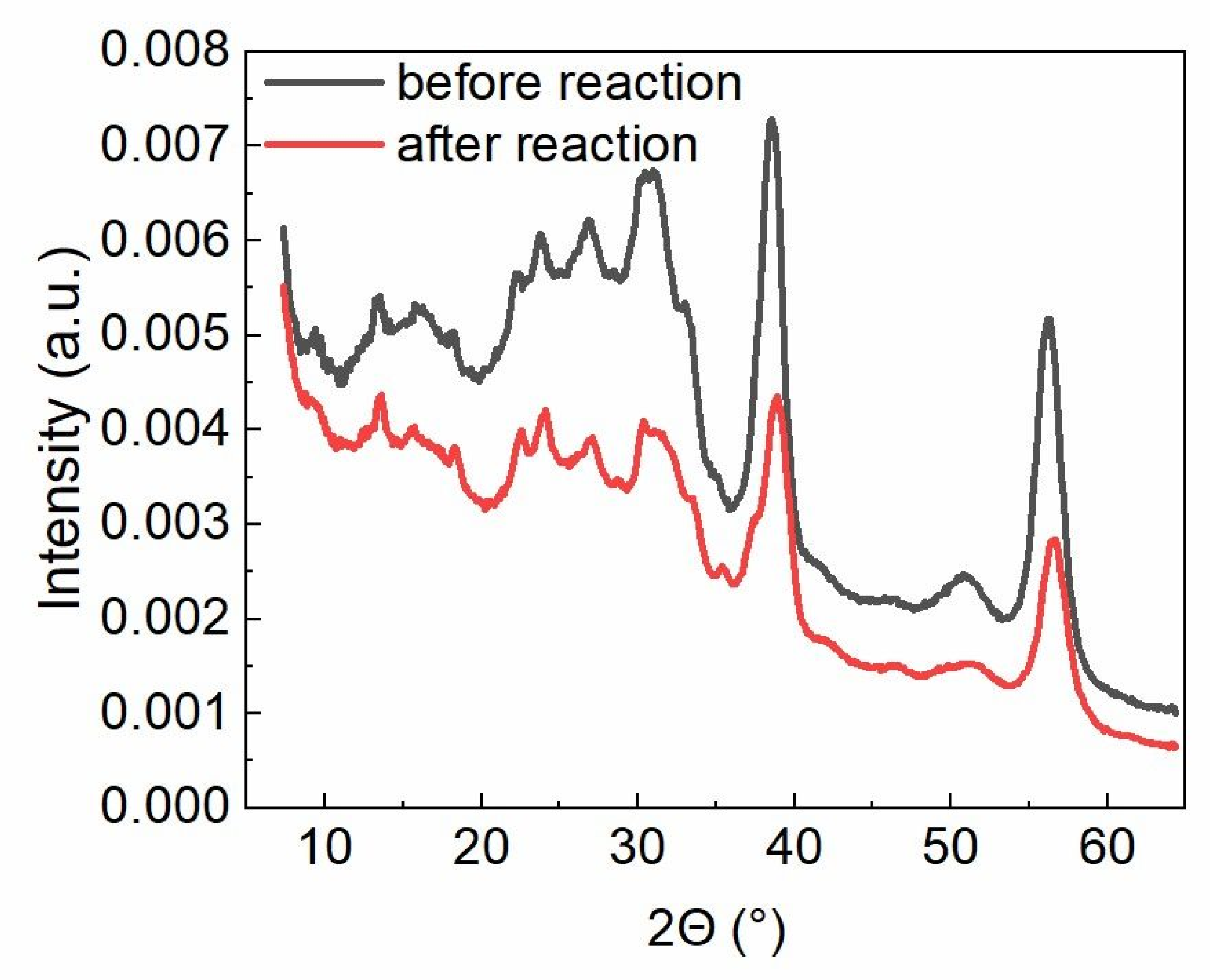

Figure S5. The WAXS spectra of the RuKY-Cl/OAc/Cl catalyst before (shown in gray) and after (shown in red) the reaction.

Table S6. Analysis of the WAX pattern of the RuKY- Cl/OAc/Cl catalyst before reaction.

**2Θ (°)**	**d-spacing (Å)**	**Phase (hkl)**
18.2	4.88	KRu_4O_8 (2,0,0), Y_2O_3 (2,0,1)
22.2	4.00	K_3AlO_3 (1,1,1)
23.8	3.73	K_3AlO_3 (-1,1,1)
26.8	3.32	K_3AlO_3 (-2,0,1), Y_2O_3 (1,1,0)
30.9	2.89	K_3AlO_3 (2,2,0)
33.1	2.71	Y_2O_3 (-1,1,2), γ-Al_2O_3 (3,1,1)
38.6	2.33	KRu_4O_8 (3,3,0)
50.8	1.80	K_3AlO_3 (-3,2,2)
56.3	1.63	γ-Al_2O_3 (4,2,2)

Table S7. Analysis of the WAX pattern of the RuKY-Cl/OAc/Cl catalyst after reaction.

**2Θ (°)**	**d-spacing (Å)**	**Phase (hkl)**
22.5	3.95	K_3AlO_3 (1,1,1), Y_2O_3 (-2,0,2)
24.0	3.70	K_3AlO_3 (-1,1,1)
27.0	3.29	K_3AlO_3 (-2,0,1)
30.3	2.94	RuO_2 (1,1,0)
31.6	2.83	Y2O3 (0,0,3), K_3AlO_3 (-2,2,1)
35.4	2.53	γ-Al_2O_3 (0,4,1)
38.9	2.31	Ru (1,0,0), γ-Al_2O_3 (-3,1,1)
42.1	2.14	K_3AlO_3 (2,4,0)
51.3	1.78	K_3AlO_3 (0,6,1)
56.6	1.62	γ-Al_2O_3 (4,2,2)

The RuKY-NO_3/OAc/NO_3 catalyst was collected via a single crystal diffractometer with a molybdenum source.

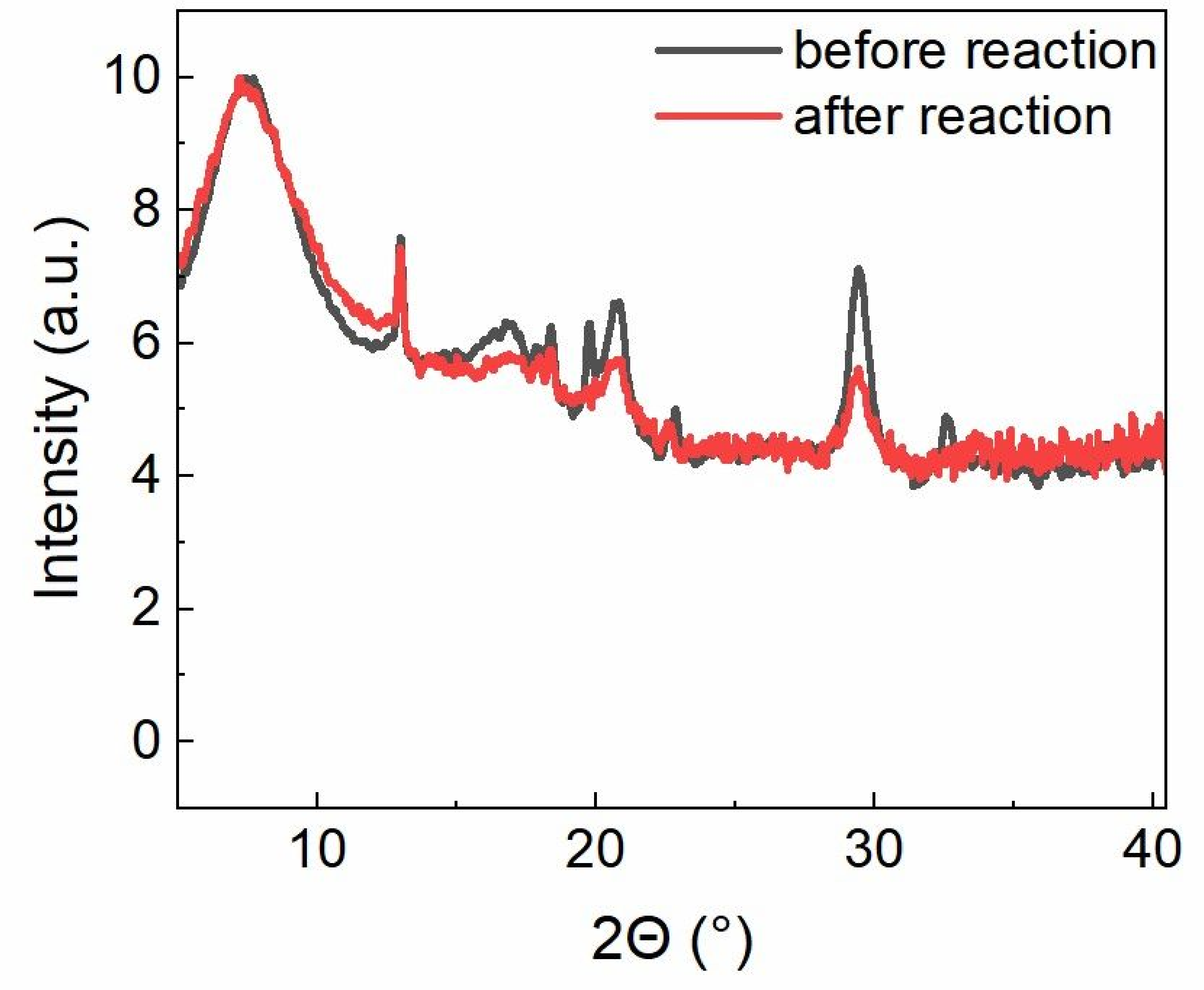

Figure S6. The XRD spectra of the RuKY-NO_3/OAc/NO_3 catalyst before (shown in gray) and after (shown in red) the reaction.

Table S8. Analysis of the WAX pattern of the RuKY-NO_3/OAc/NO_3 catalyst before reaction.

2Θ (°)	d-spacing (Å)	Phase (h,k,l)
7.6	5.32	γ-Al_2O_3 (1,1,1)
13.0	3.14	RuO_2 (1,1,0)
16.8	2.42	K_3AlO_3 (2,0,1)
17.9	2.28	γ-Al_2O_3 (2,2,2), Y_2O_3 (3,3,2)
18.4	2.22	RuO_2 (2,0,0), K_3AlO_3 (1,1,6)
19.0	2.15	K_3AlO_3 (2,0,5)
19.8	2.07	K_3AlO_3 (1,0,10), Y_2O_3 (4,3,1)
20.8	1.96	γ-Al_2O_3 (4,0,0)
22.9	1.79	γ-Al_2O_3 (3,3,1)
29.4	1.40	RuO_2 (1,1,2), γ-Al_2O_3 (4,4,0), K_3AlO_3 (1,1,14)
32.6	1.27	K_3AlO_3 (0,0,18)
38.4	1.08	Y_2O_3 (9,3,2)

Table S9. Analysis of the WAX pattern of the RuKY- NO_3/OAc/NO_3 catalyst after reaction.

2Θ (°)	d-spacing (Å)	Phase (hkl)
7.6	5.32	γ-Al_2O_3 (1,1,1)
13.0	3.14	RuO_2 (1,1,0), Y_2O_3 (1,1,1)
18.0	2.27	γ-Al_2O_3 (2,2,2)
18.4	2.22	RuO_2 (2,0,0), Y_2O_3 (-2,0,4)
20.7	1.97	γ-Al_2O_3 (4,0,0)
22.6	1.81	γ-Al_2O_3 (3,1,1), Y_2O_3 (1,1,4)
29.4	1.40	γ-Al_2O_3 (4,4,0), RuO_2 (1,1,2)

Scanning Electron Microscopy (SEM) and Electron Dispersion Spectroscopy (EDX)

The SEM and subsequent EDX data are shown for all catalysts both before and after reaction and for the cross section and outer shell of the catalyst. Agglomeration was not noticed in any of the catalysts over the course of the reaction.

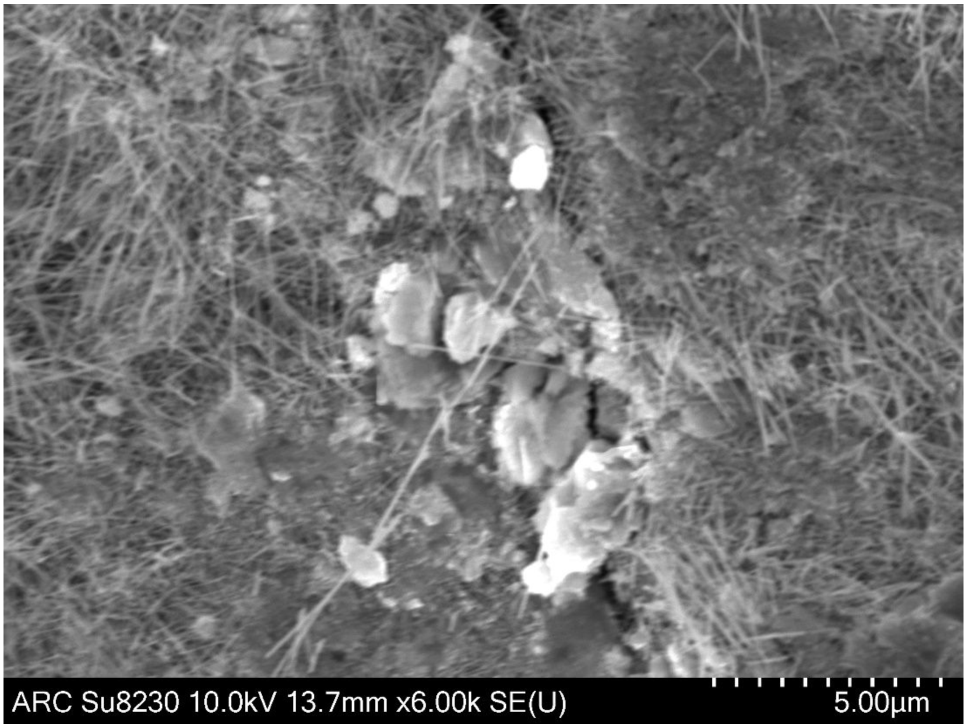

Figure S7. SEM of the outer shell of the RuKY-Cl/OAc/Cl after calcination.

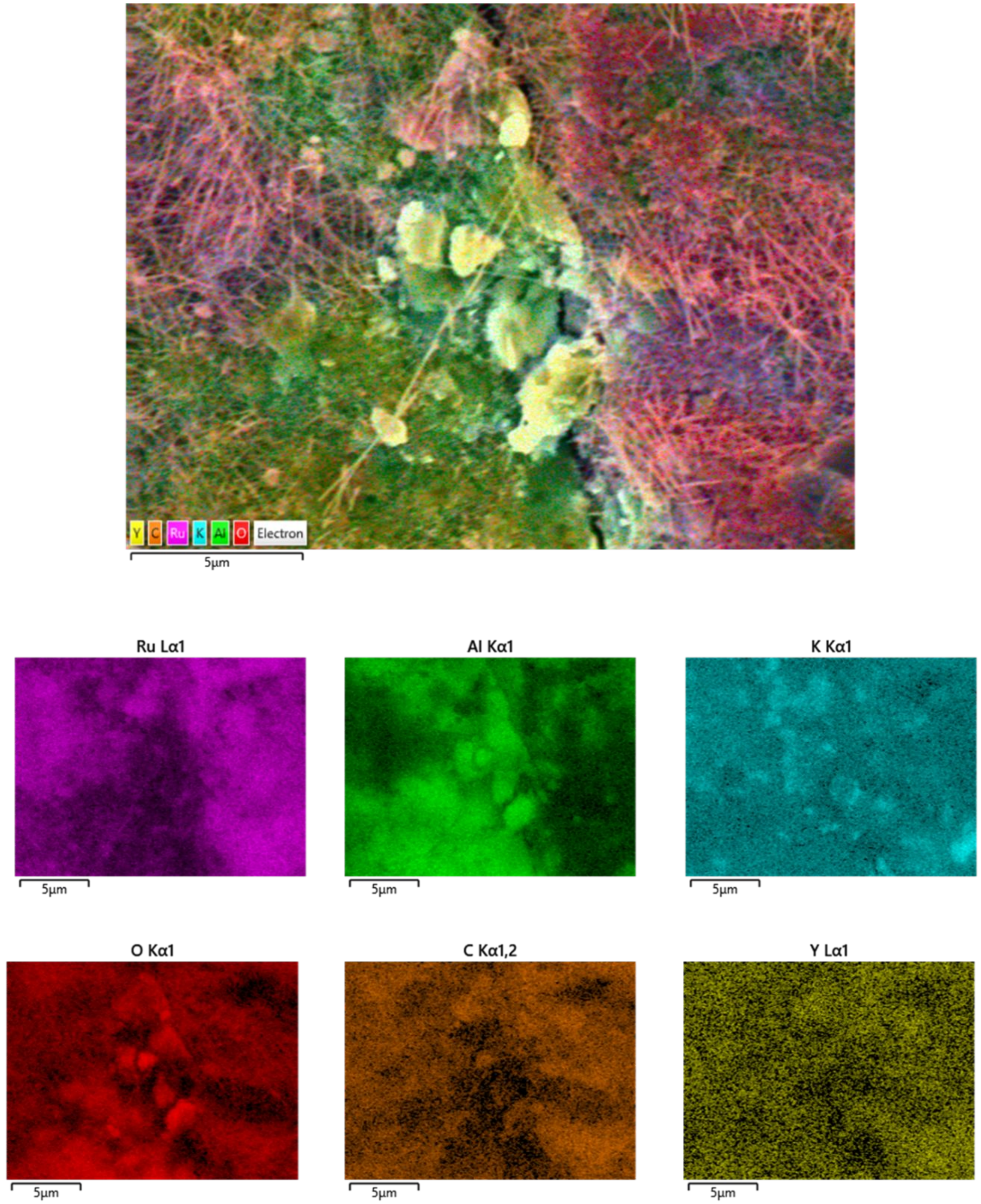

Figure S8. SEM with EDX overlayed of the outer portion of the RuKY- Cl/OAc/Cl pellet catalyst after calcination.

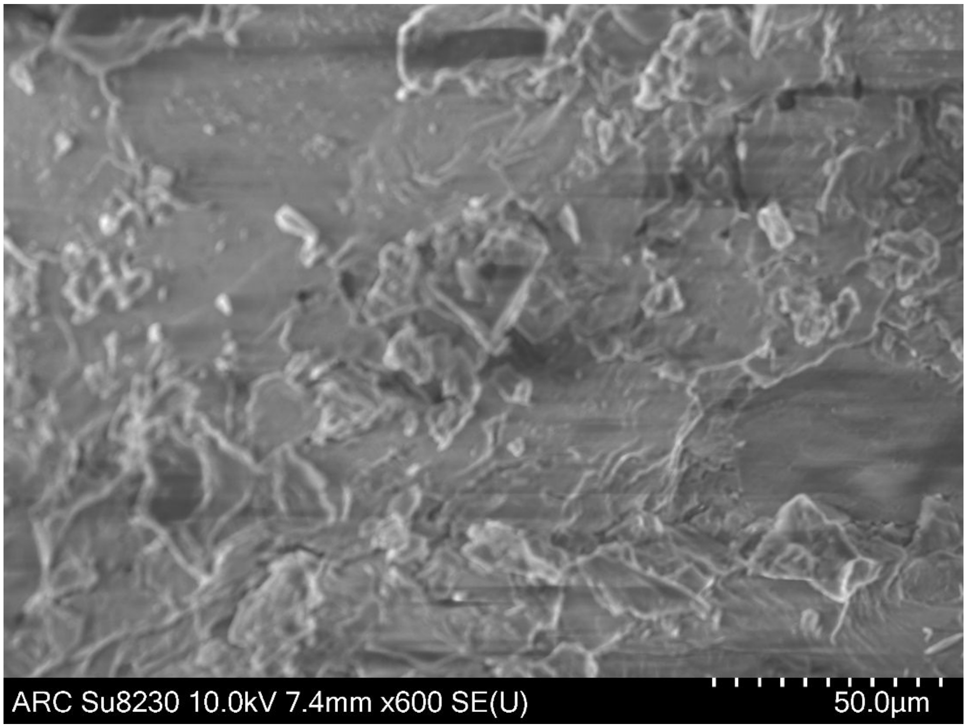

Figure S9. SEM of the intersection of the RuKY-Cl/OAc/Cl after calcination.

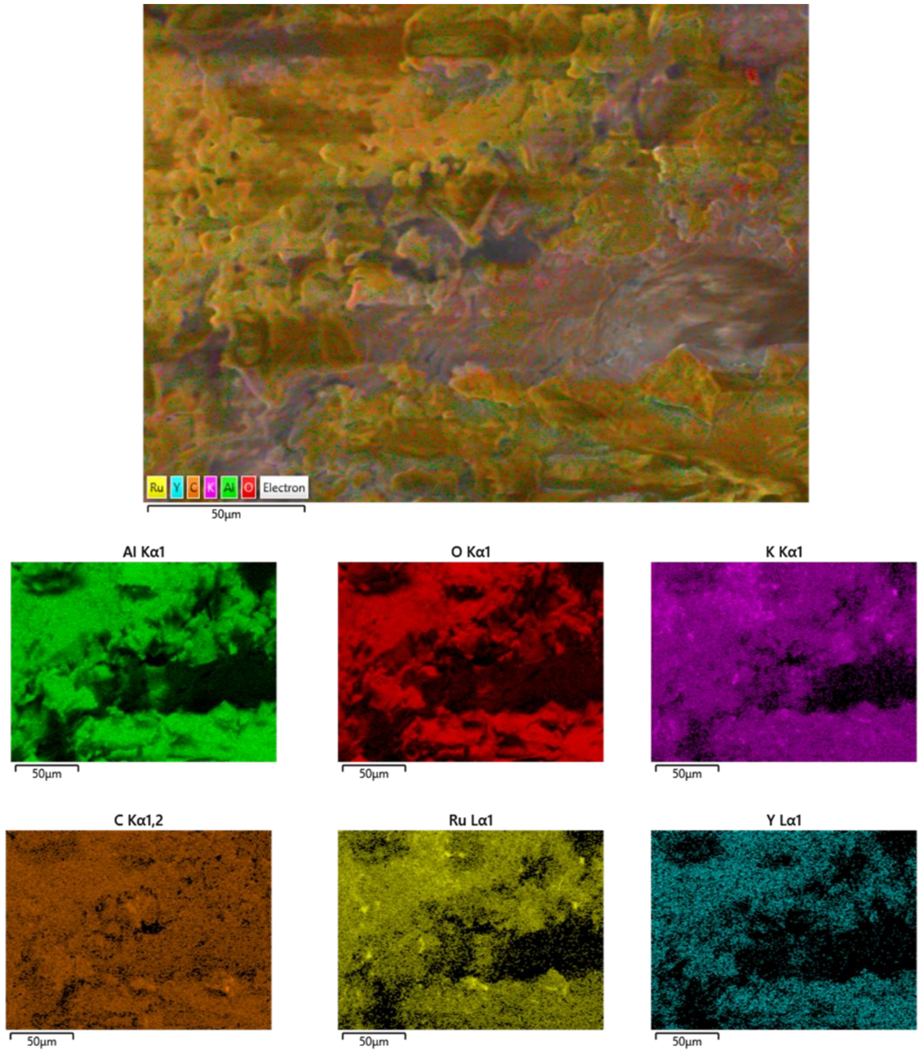

Figure S10. SEM with EDX overlayed of the cross section of the RuKY-Cl/OAc/Cl pellet catalyst after calcination.

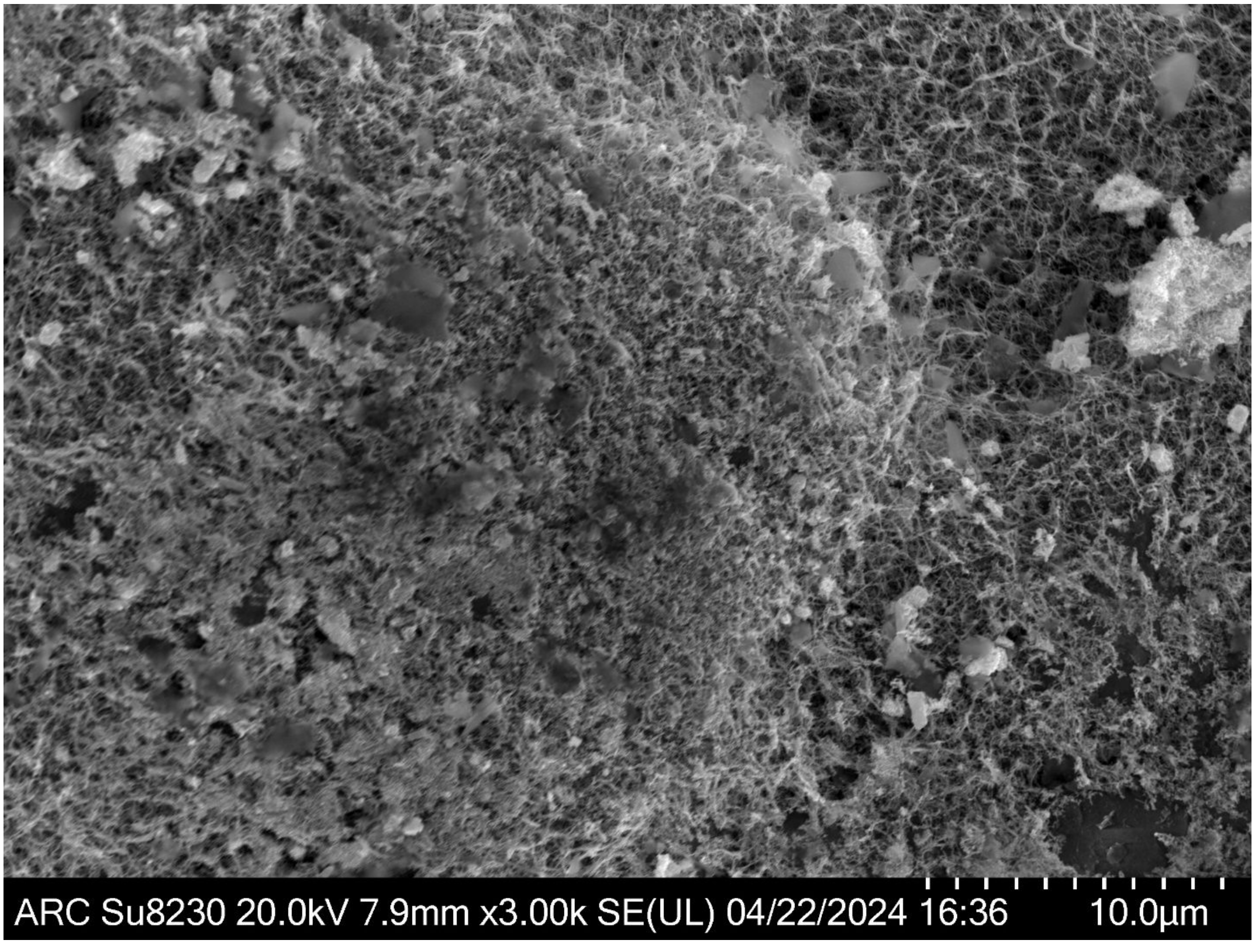

Figure S11. SEM of the outer shell of the RuKY-Cl/OAc/Cl after reaction.

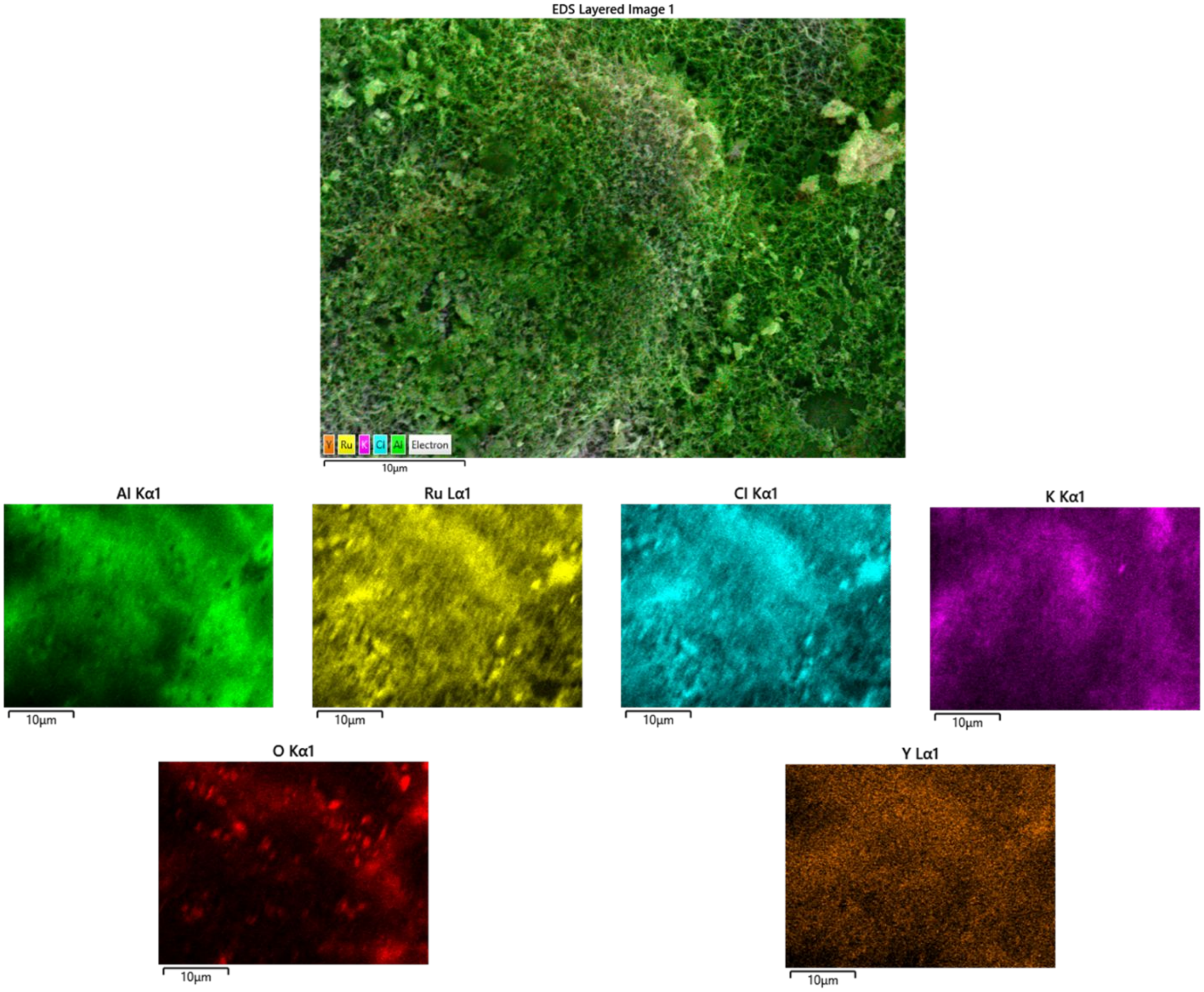

Figure S12. EDX overlay of the SEM shown in Figure S11.

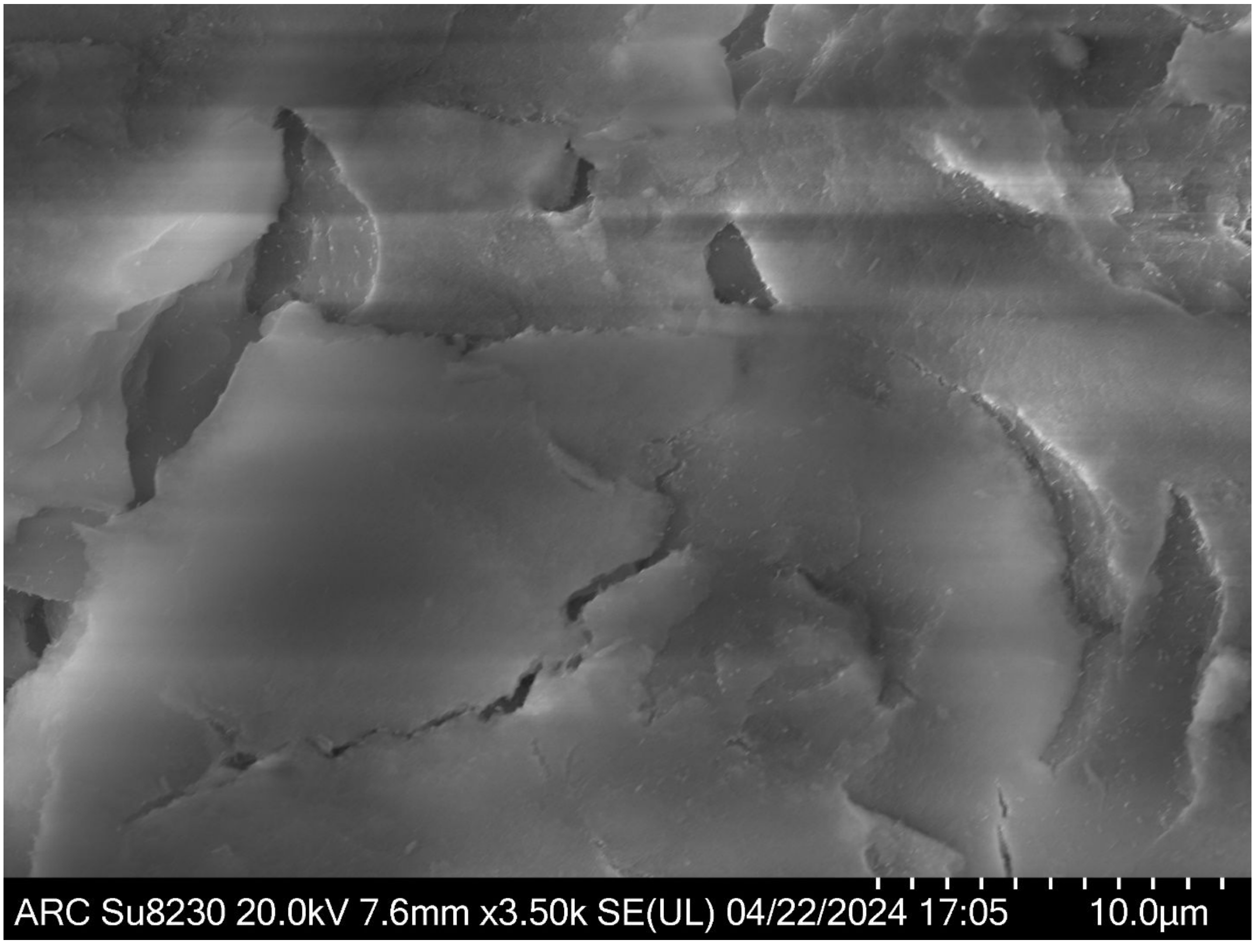

Figure S13. SEM of the intersection of the RuKY-Cl/OAc/Cl after reaction.

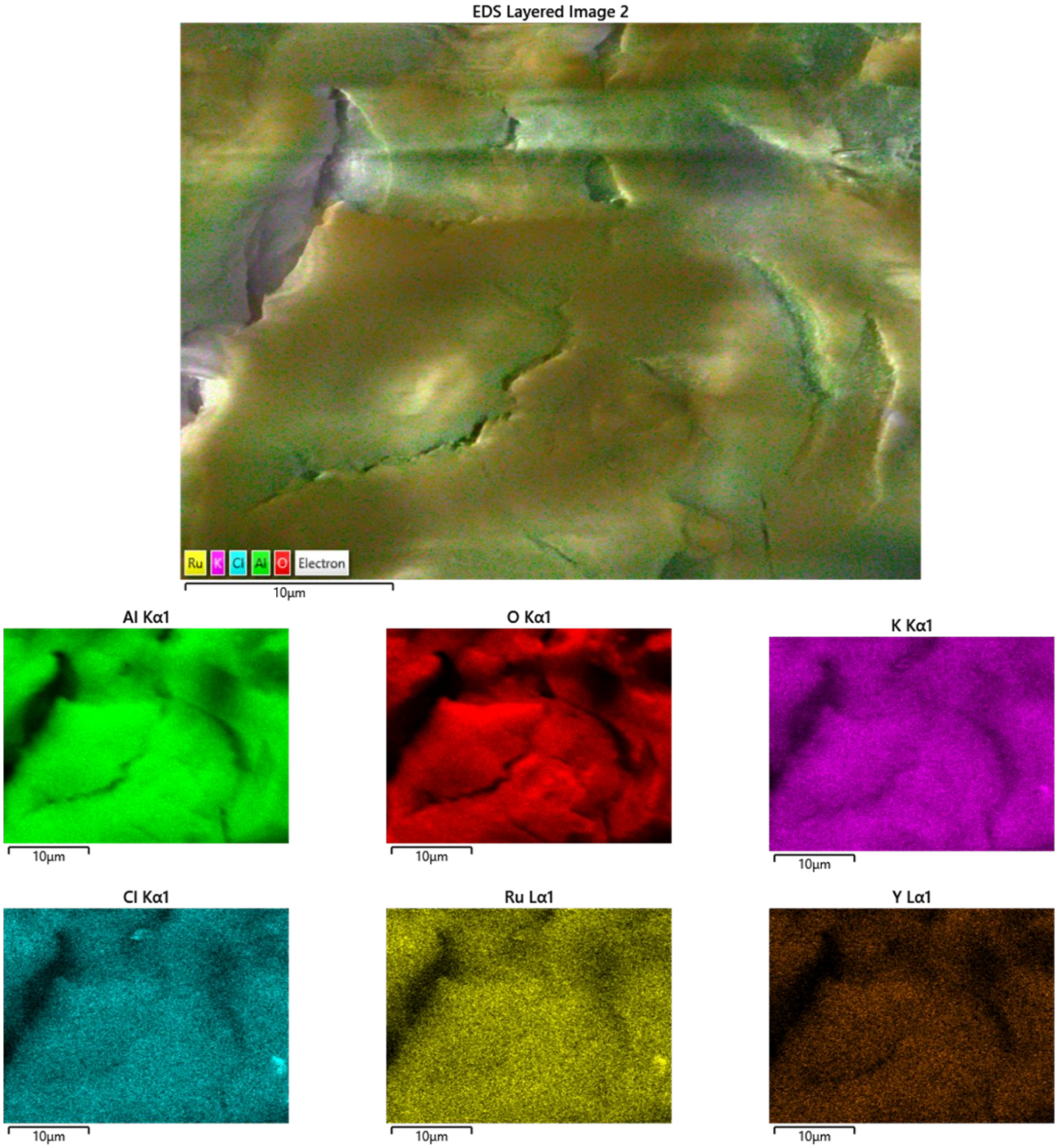

Figure S14. EDX overlayed maps of the SEM shown in Figure S13.

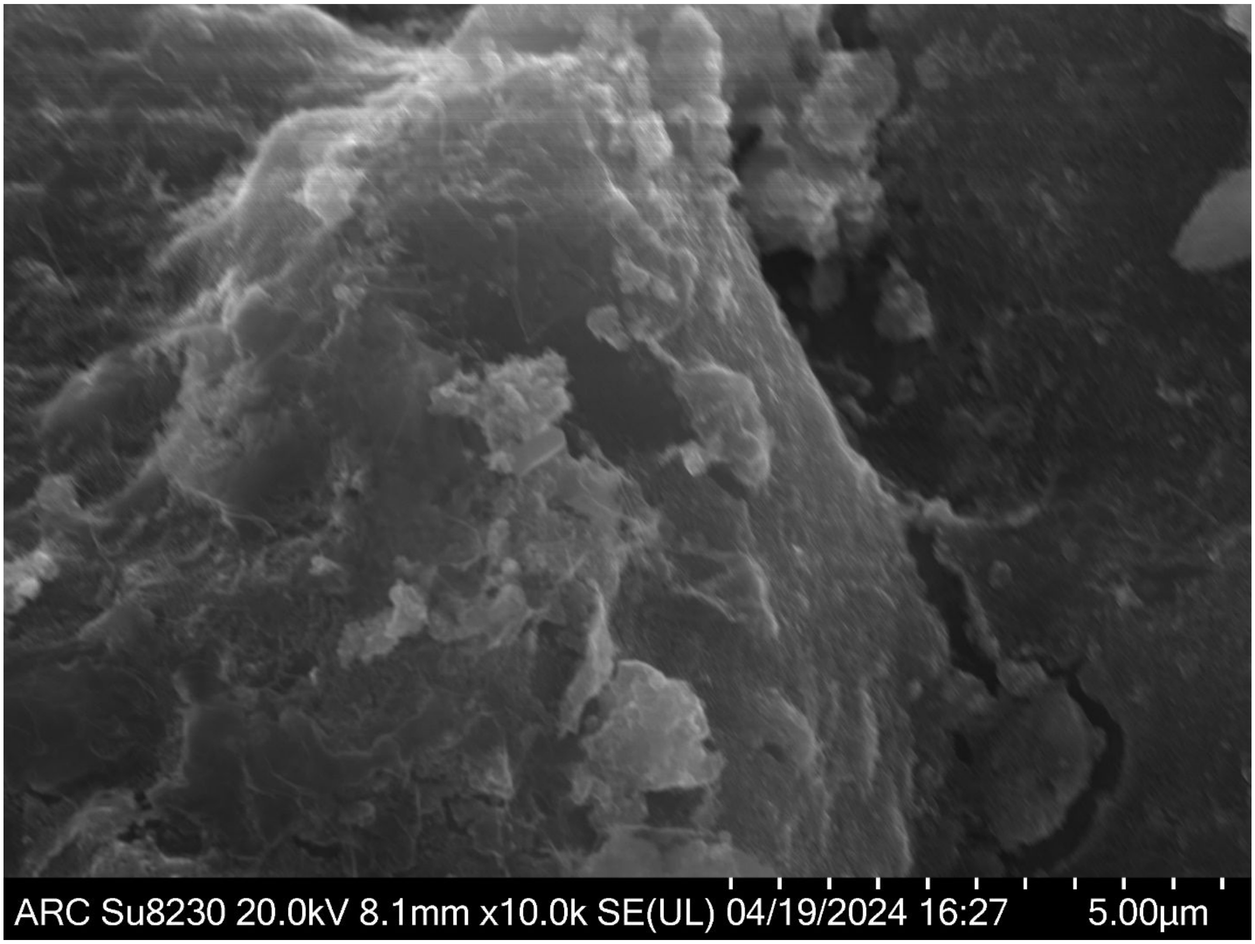

Figure S15. SEM of RuKY-NO_3/NO_3/NO_3 after calcination.

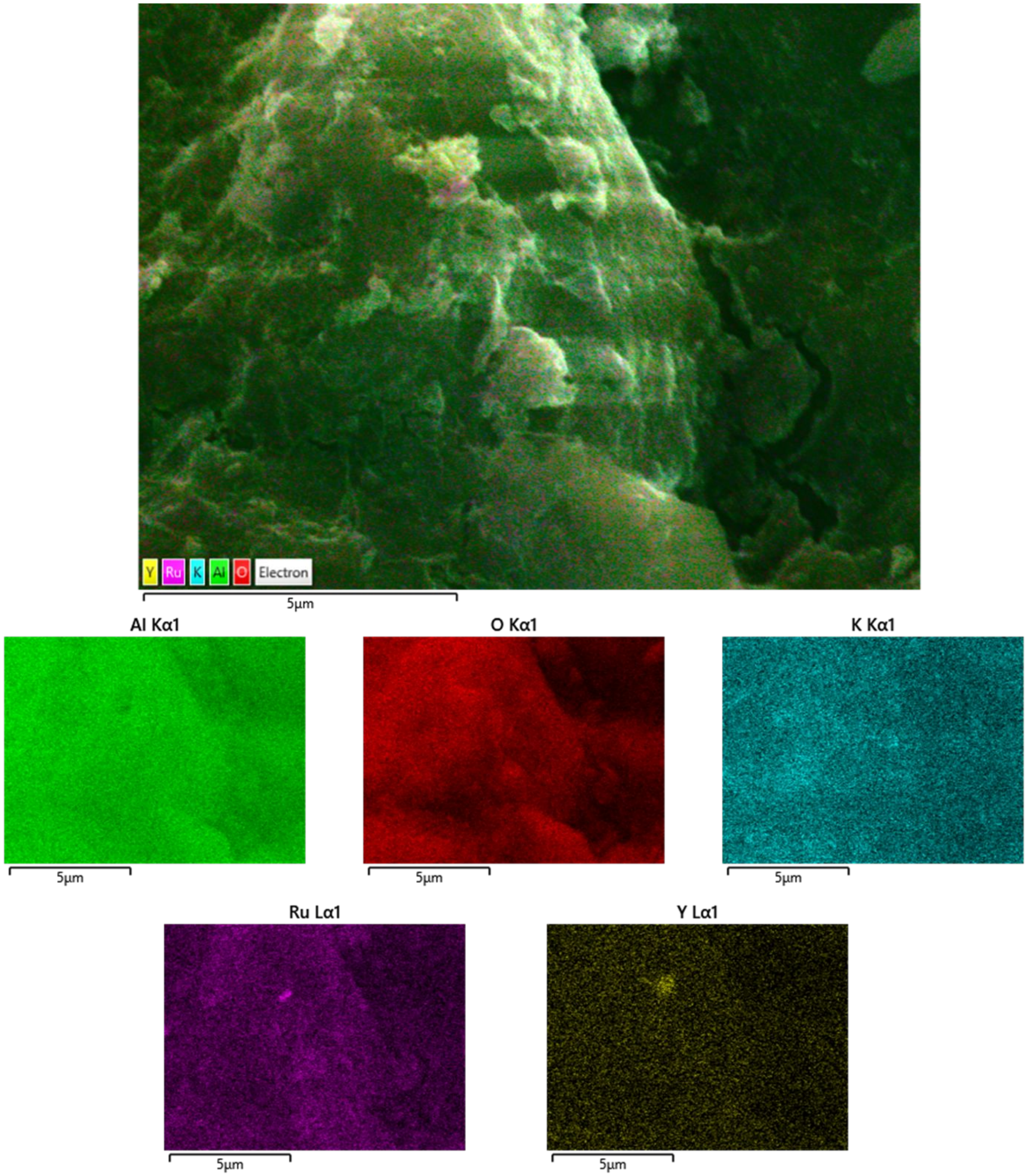

Figure S16. EDX overlay of the SEM shown in Figure S15.

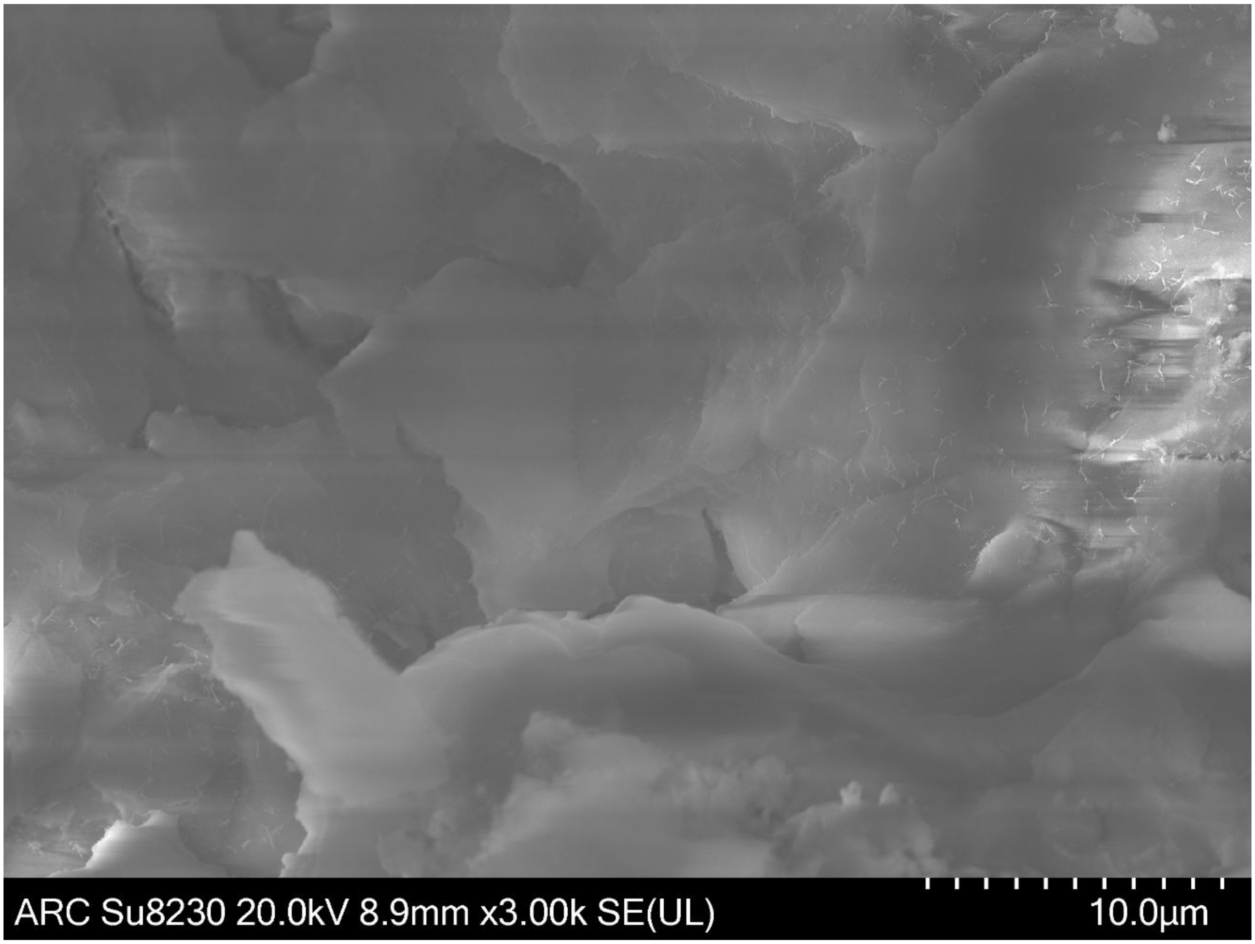

Figure S17. SEM of the cross section of the RuKY-NO_3/NO_3/NO_3 after calcination.

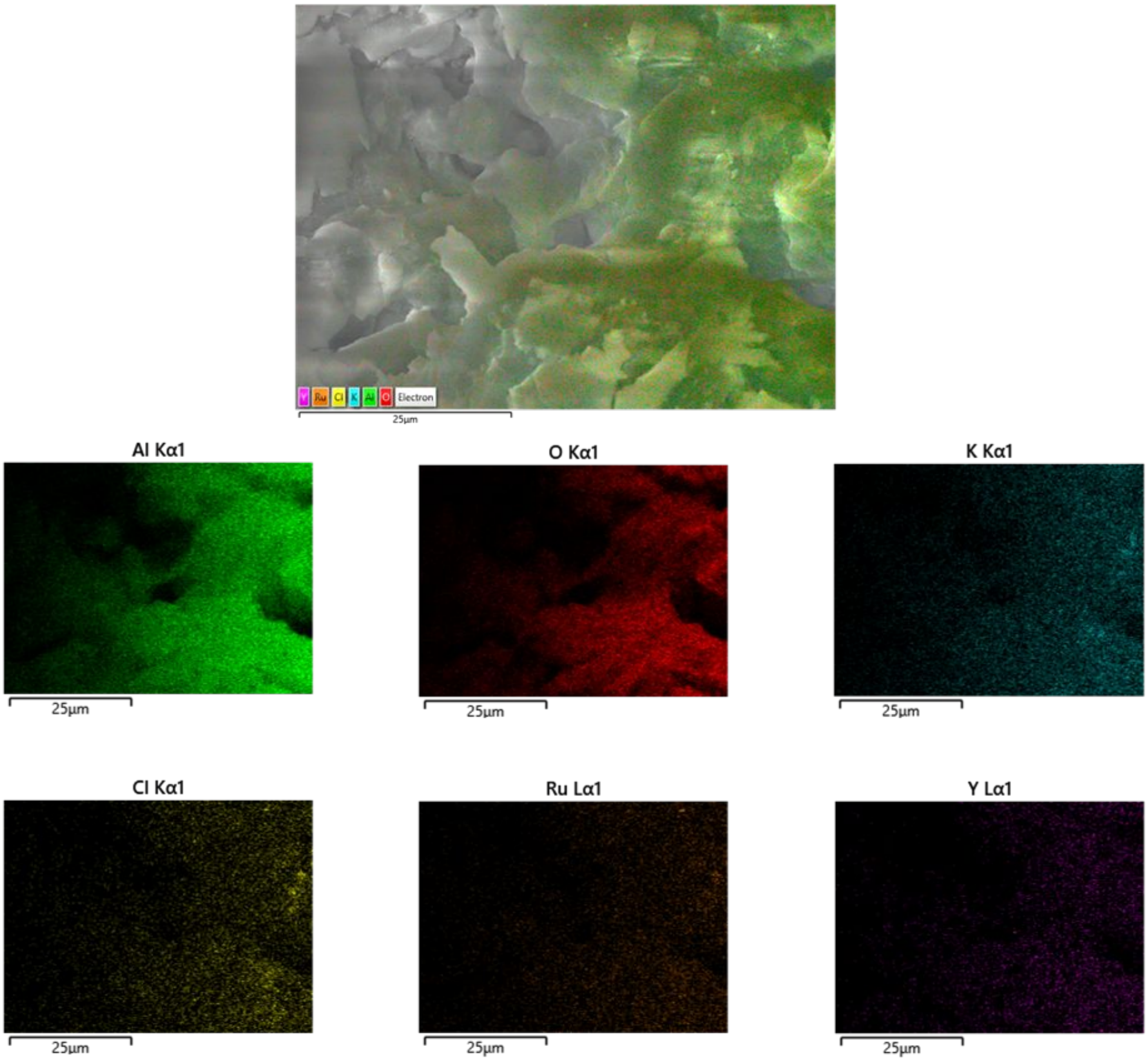

Figure S18. EDX overlay of the SEM shown in Figure S17.

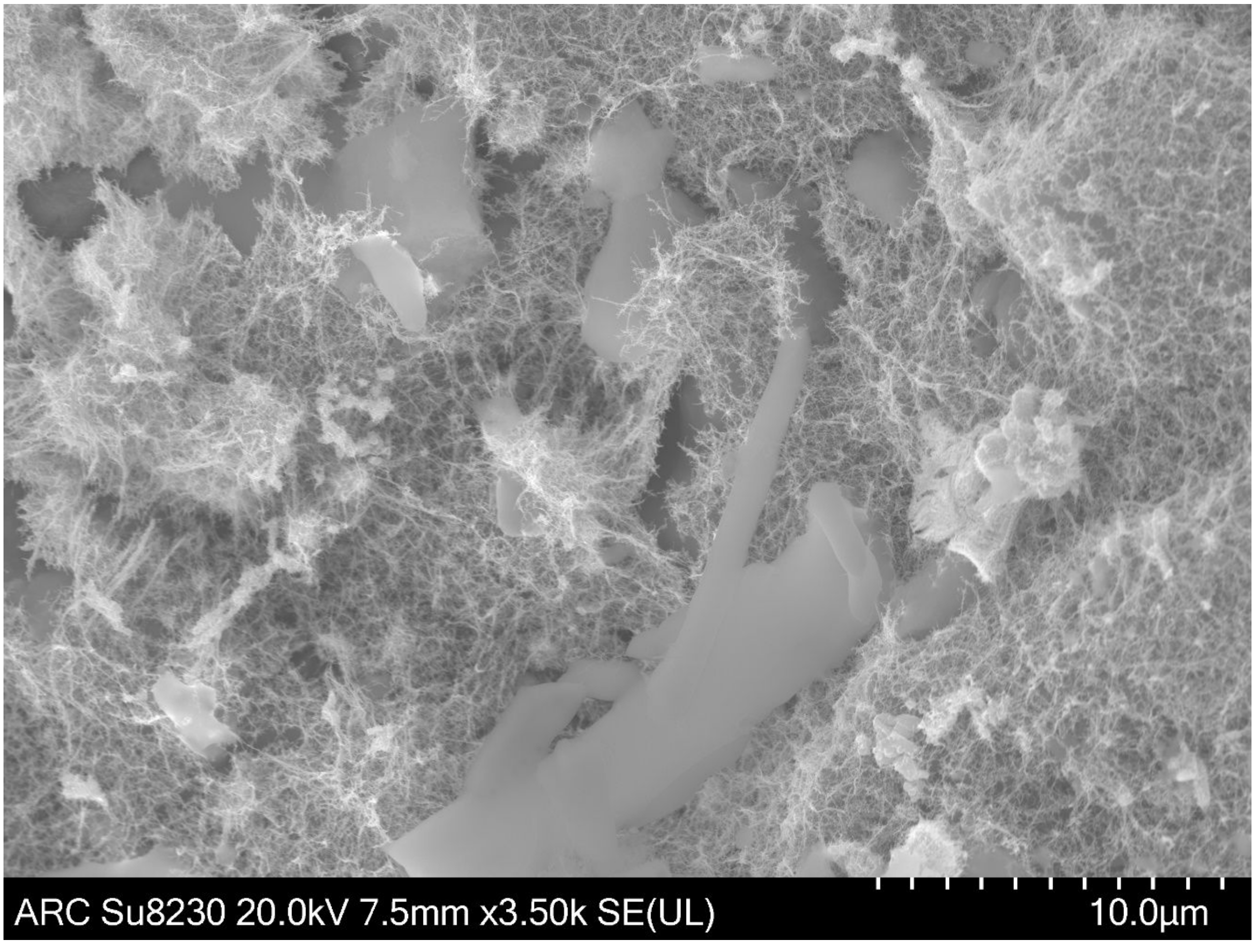

Figure S19. SEM of the outer shell of RuKY-NO_3/NO_3/NO_3 after calcination.

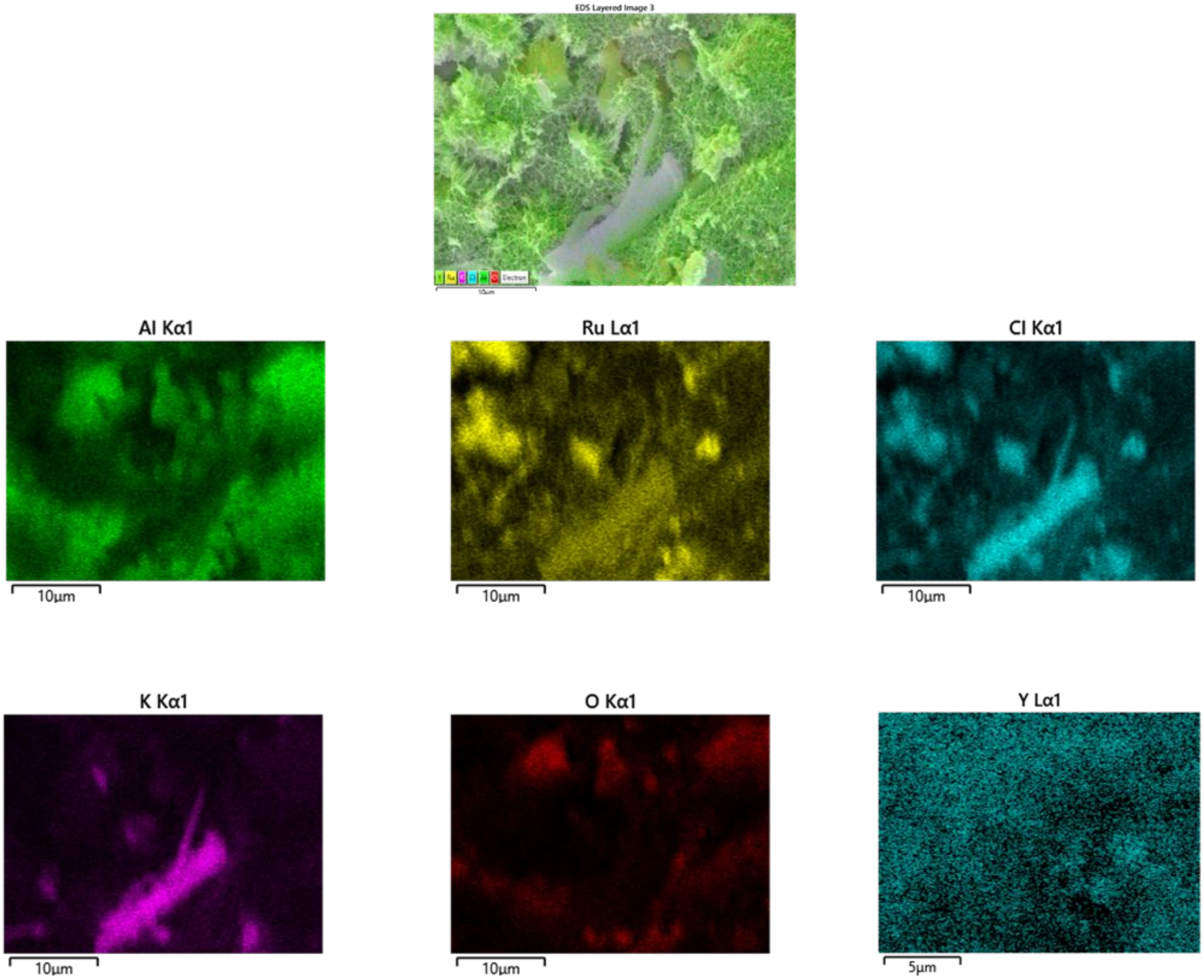

Figure S20. EDX-overlayed of the SEM shown in Figure S19.

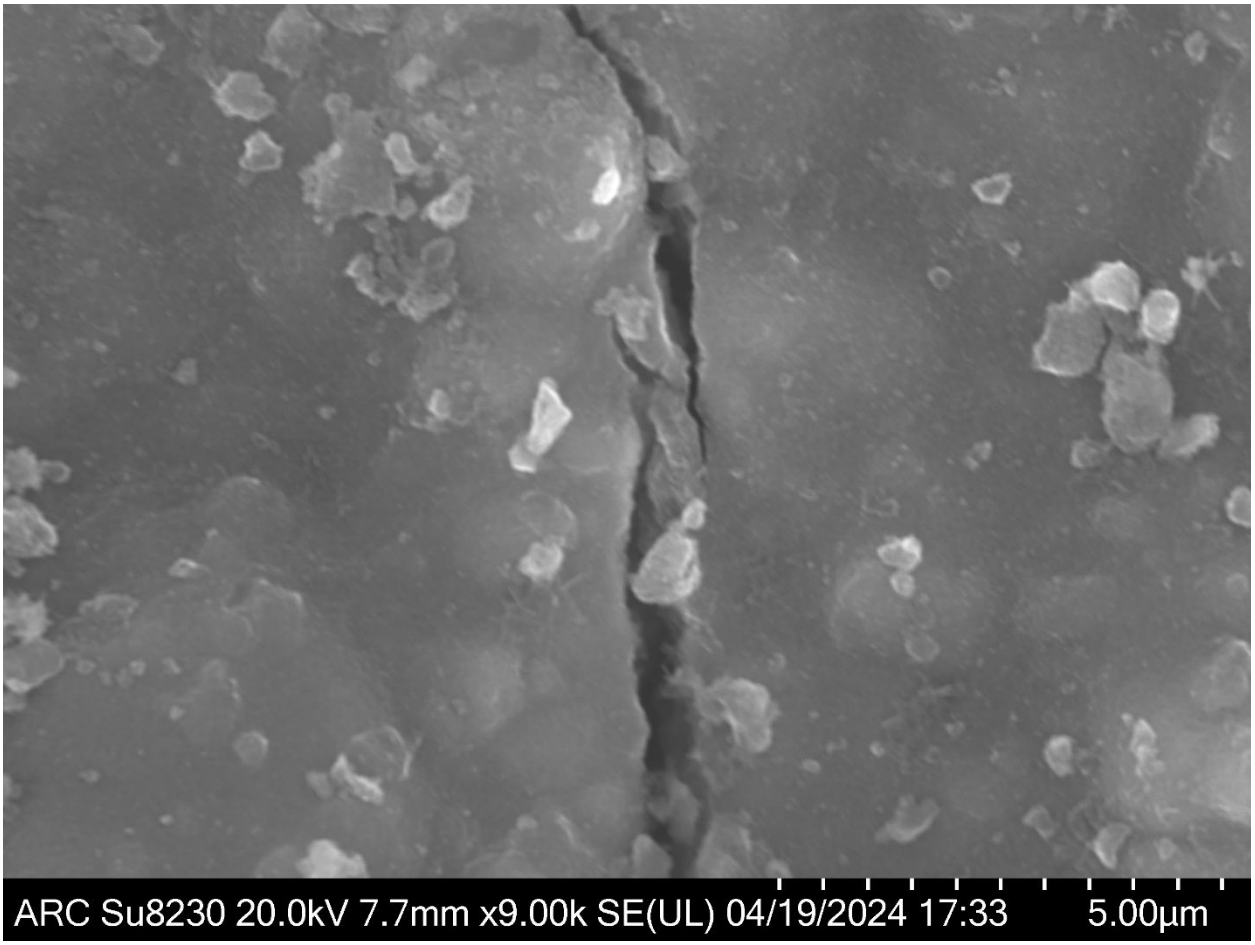

Figure S21. SEM of outer shell RuKY-NO_3/NO_3/NO_3 after reaction.

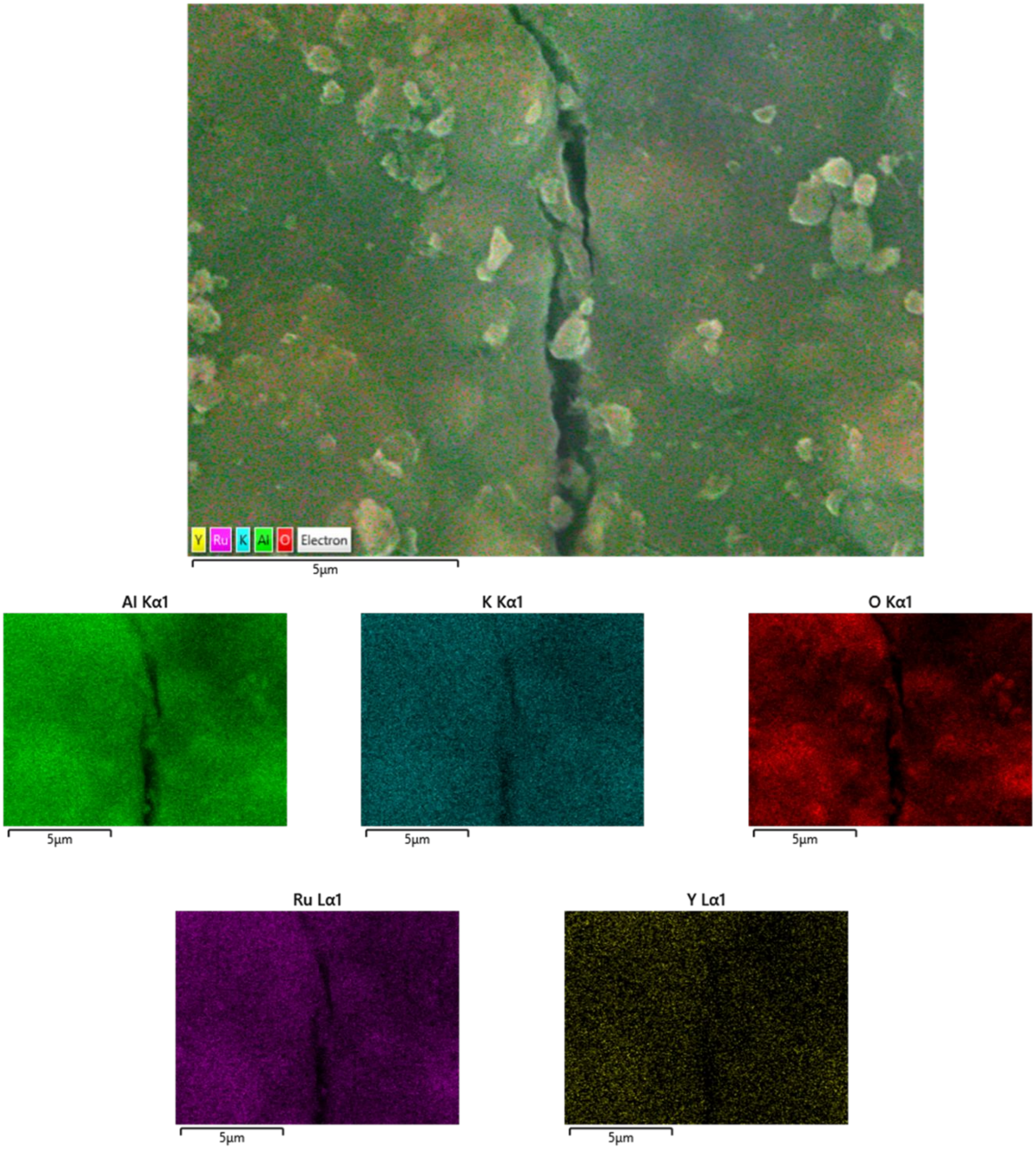

Figure S22. EDX-overlay of the SEM shown in Figure S21.

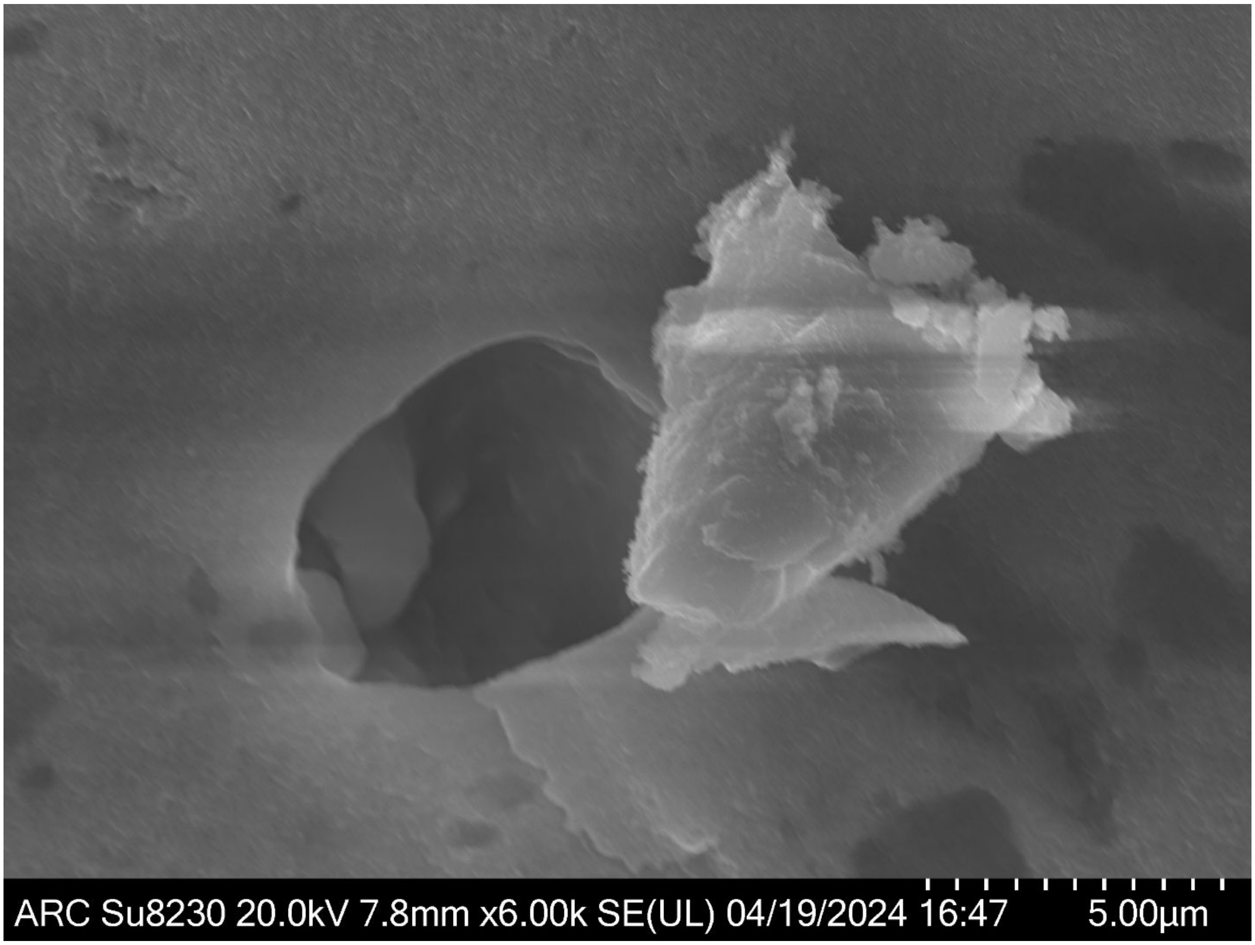

Figure S23. SEM of inner cross section RuKY-NO_3/NO_3/NO_3 after reaction.

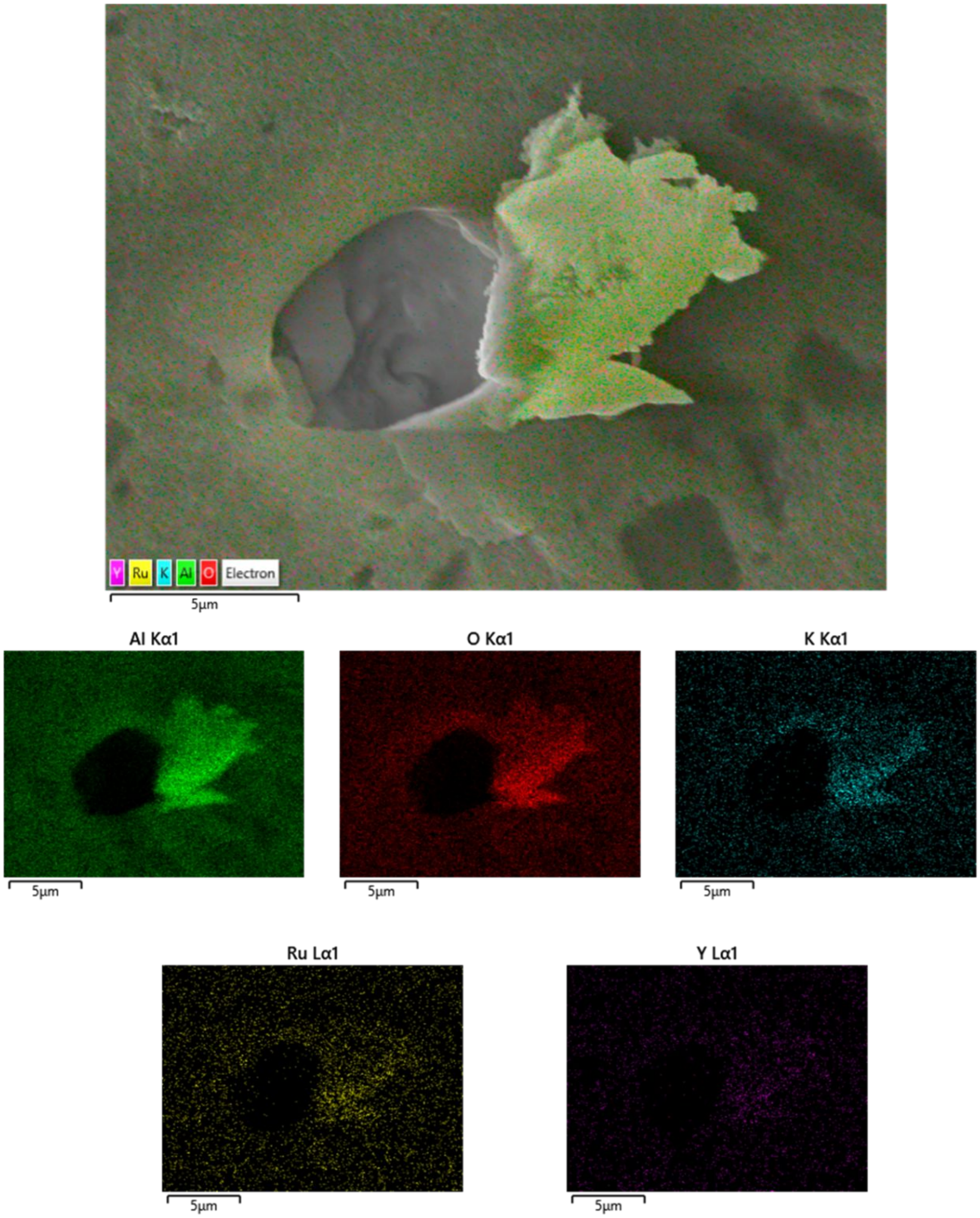

Figure S24. EDX-overlayed on SEM shown in Figure S23.

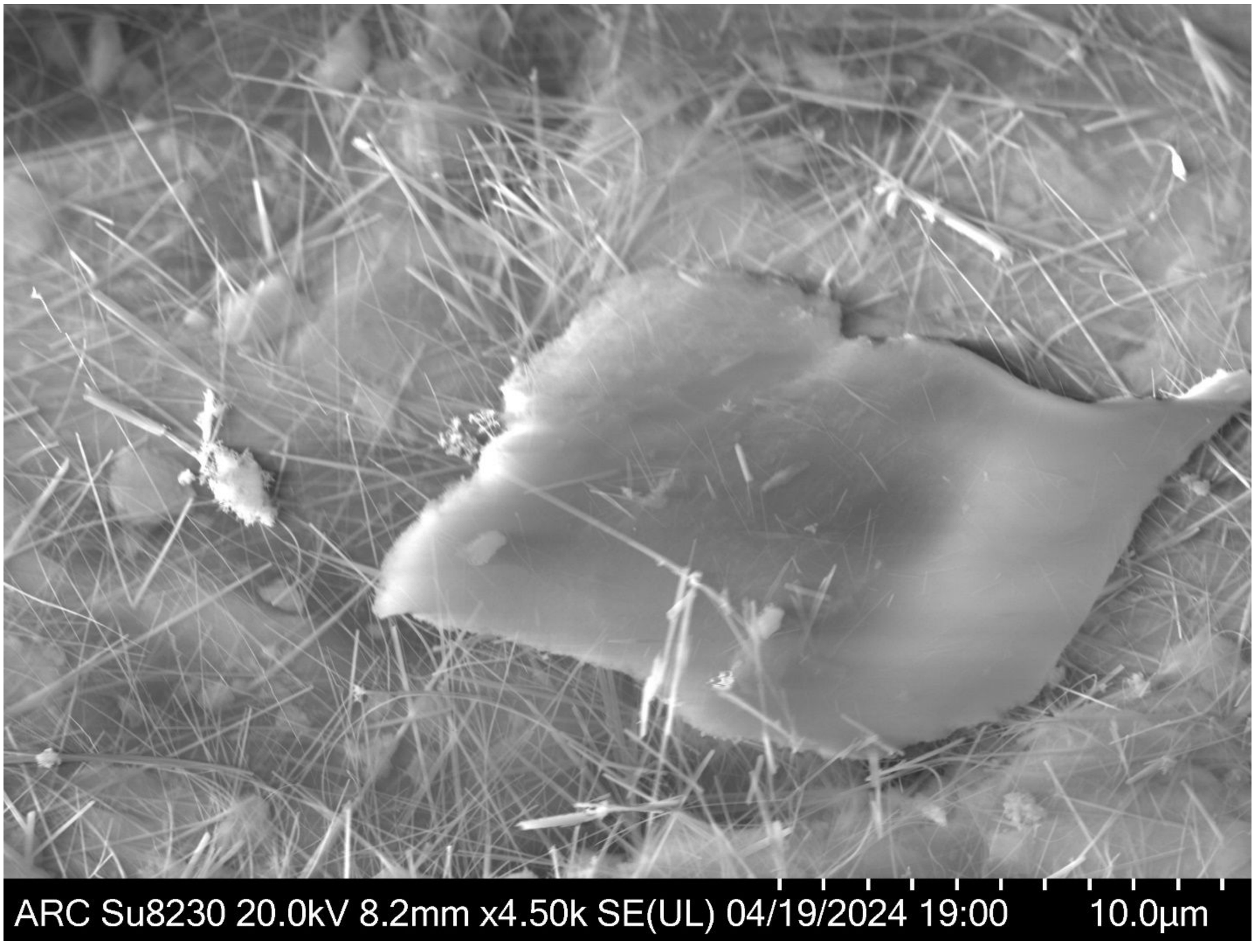

Figure S25. RuKY-Cl/NO_3/Cl outer section after calcination.

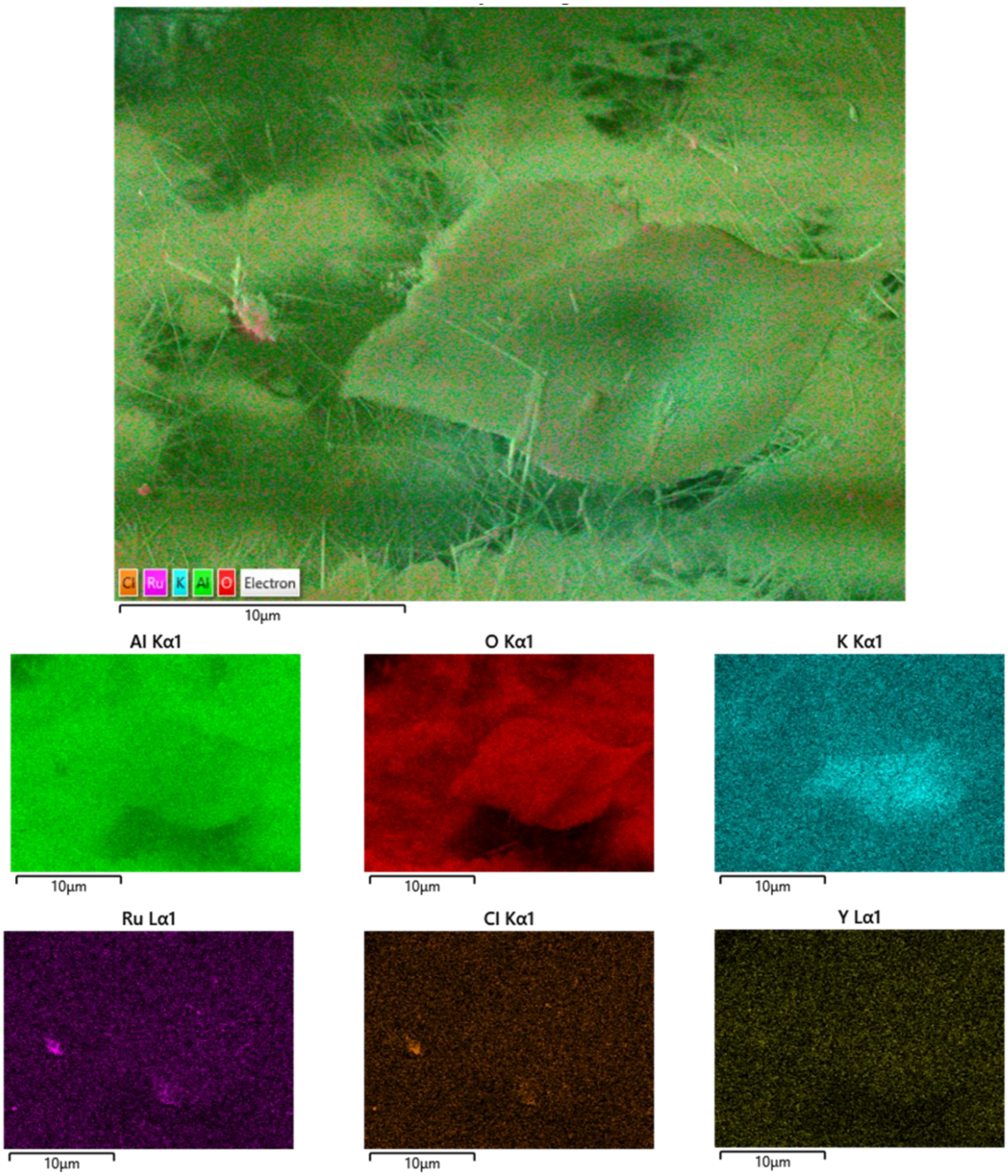

Figure S26. EDX overlay of the SEM shown in Figure S25.

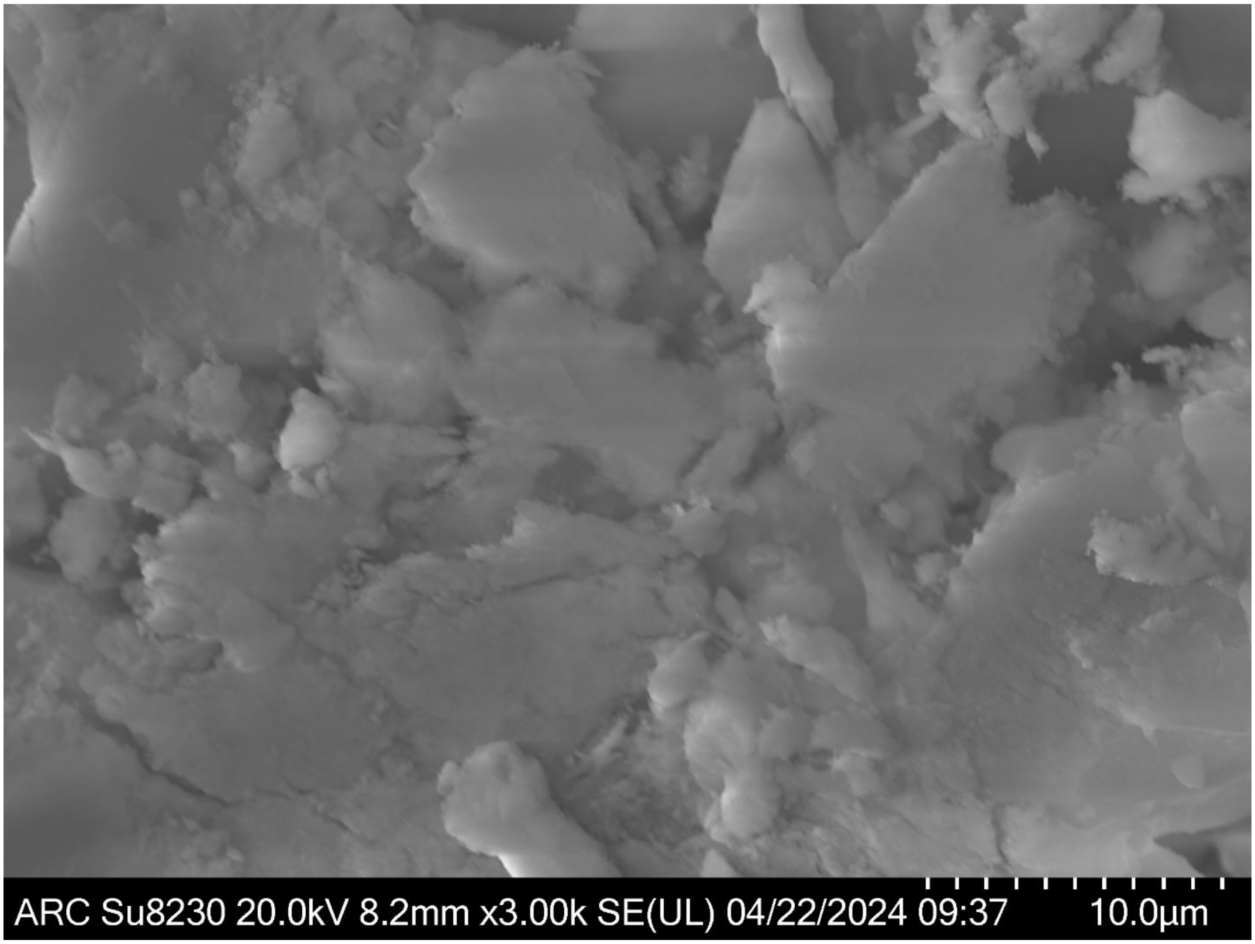

Figure S27. SEM of the cross section of the RuKY-Cl/NO_3/Cl catalyst after calcination.

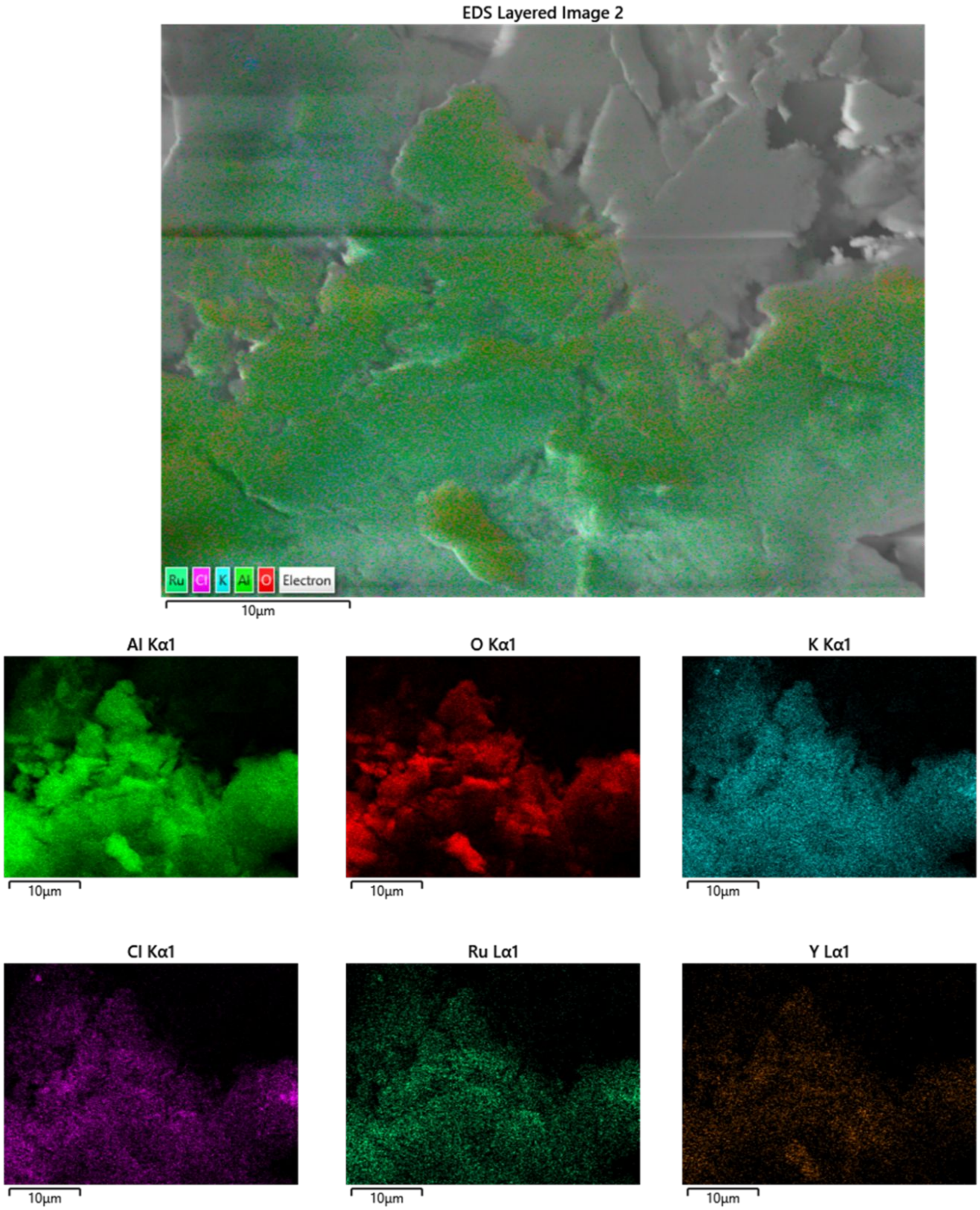

Figure S28. EDX overlay of the SEM shown in Figure S27.

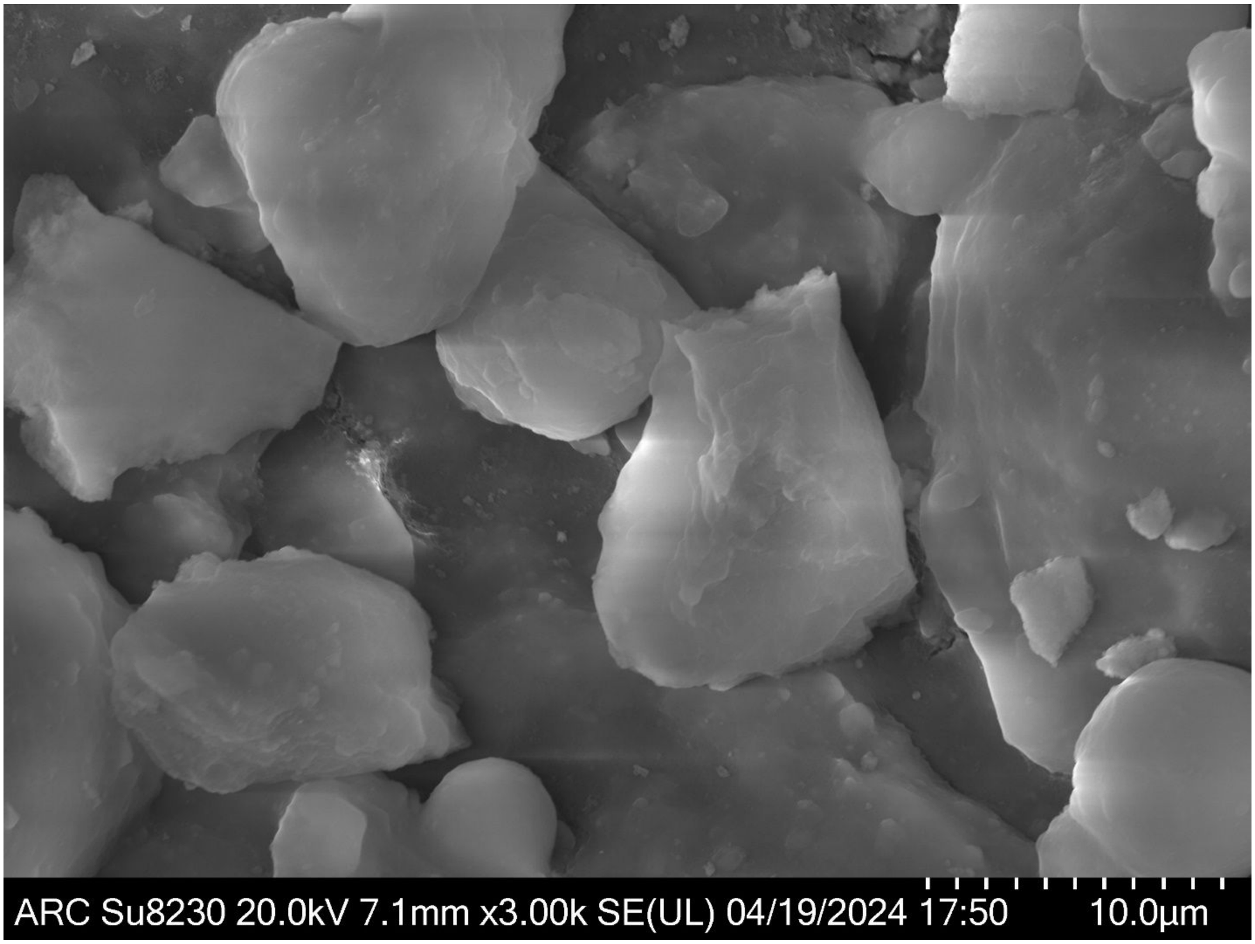

Figure S29. RuKY-Cl/NO_3/Cl outer section after reaction.

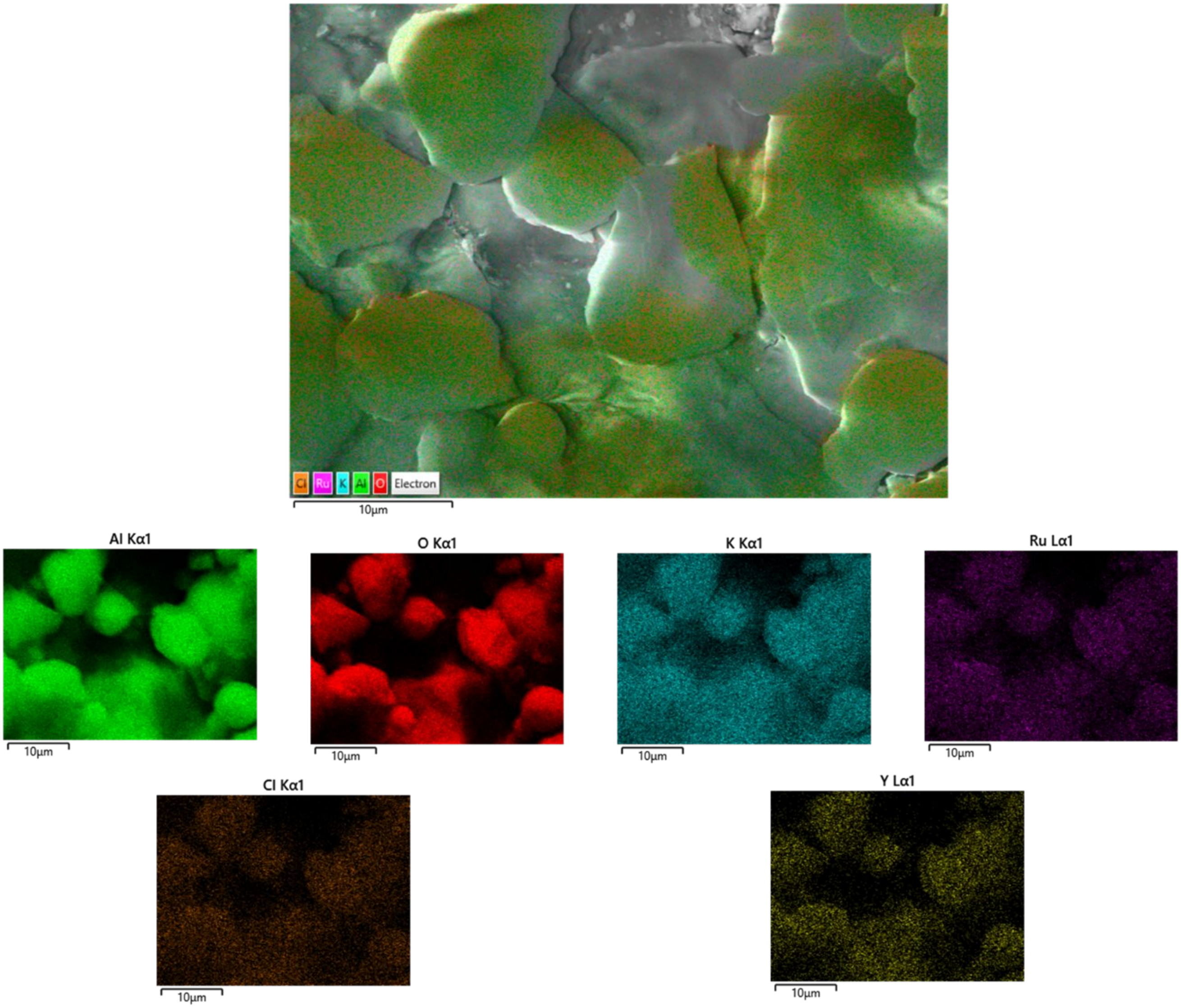

Figure S30. SEM-EDX of the RuKY-Cl/NO_3/Cl catalyst after calcination of the outer shell.

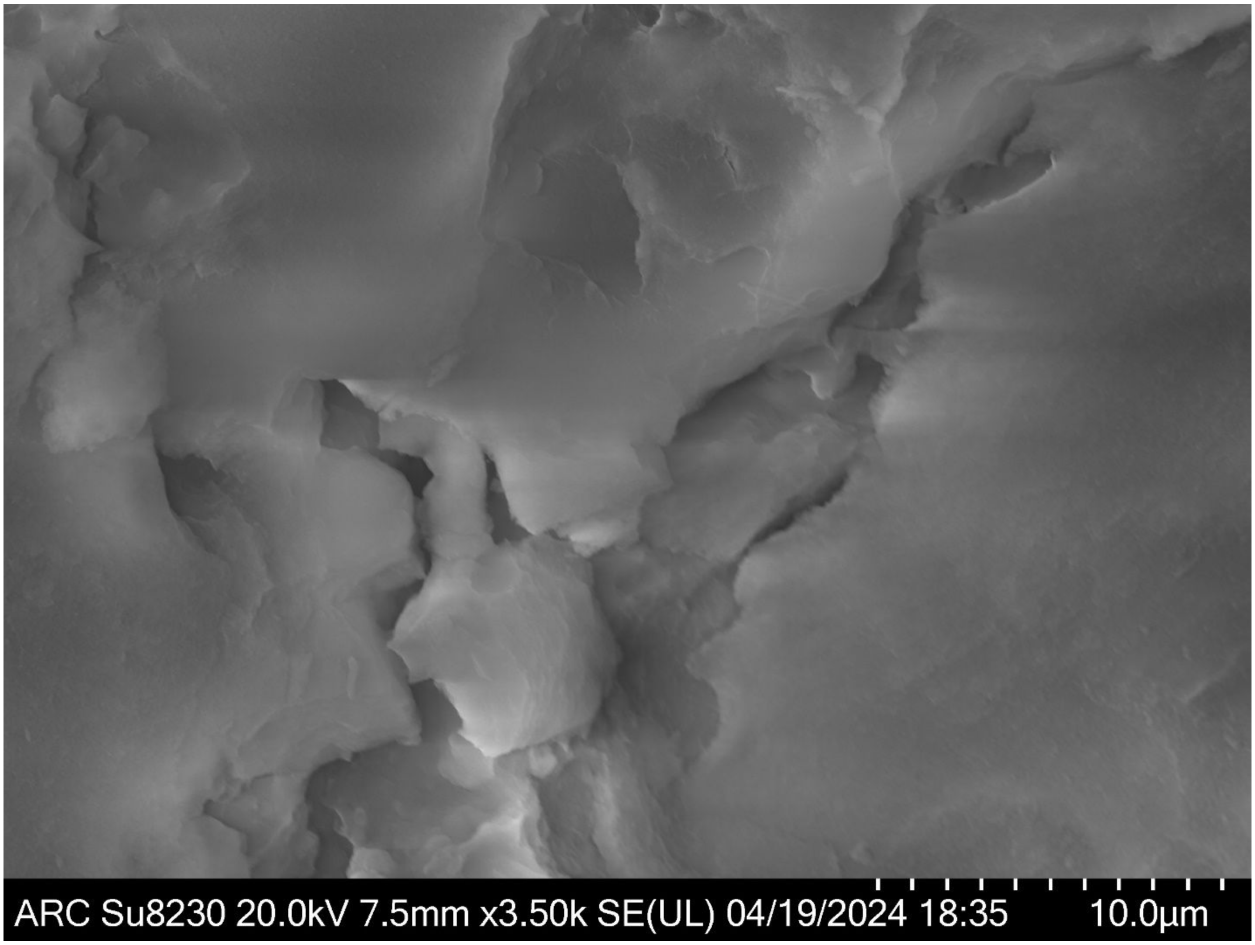

Figure S31. RuKY-Cl/NO_3/Cl cross section after reaction.

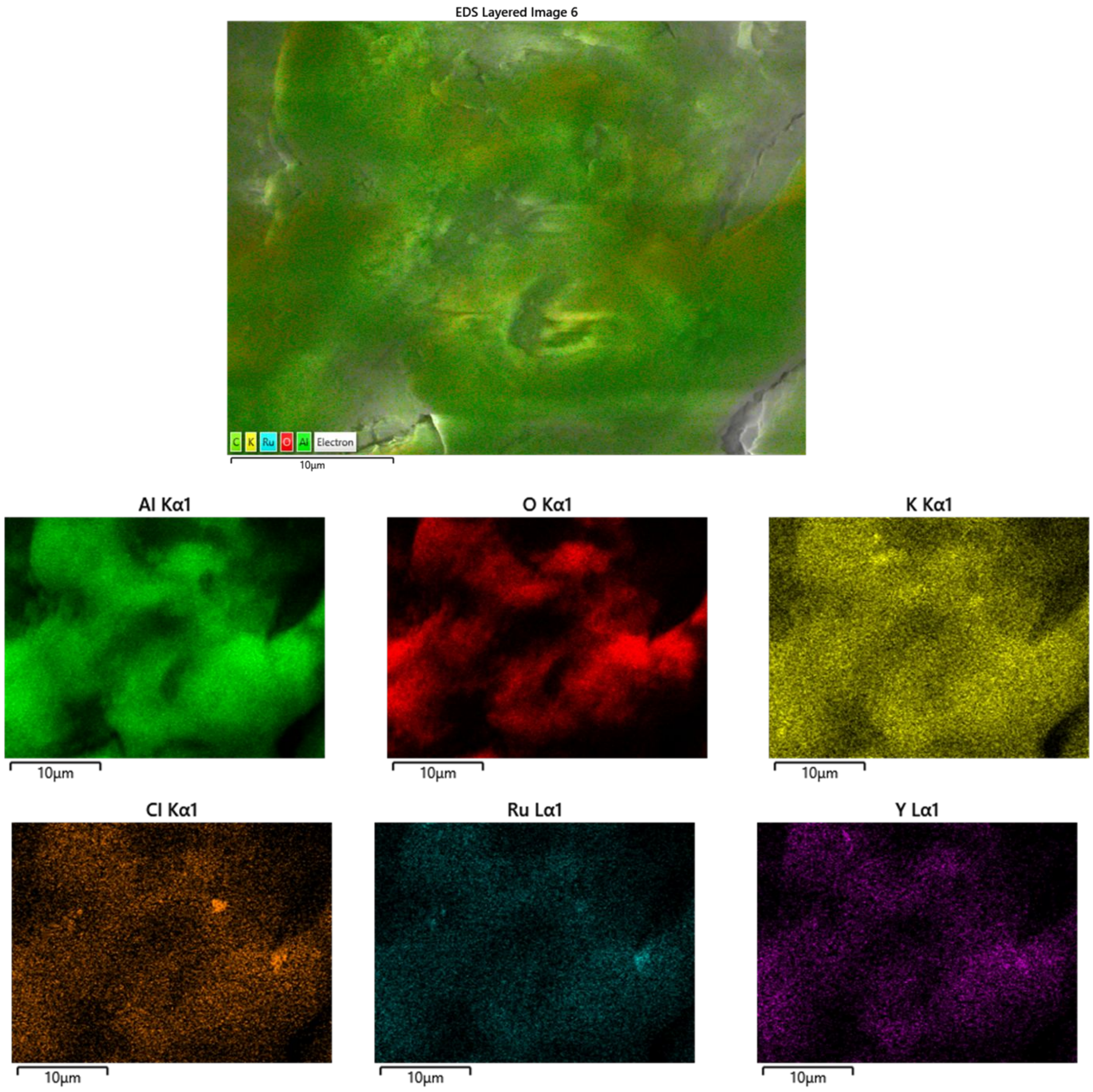

Figure S32. SEM-EDX of the RuKY-Cl/NO_3/Cl catalyst after calcination of the inner cross section.

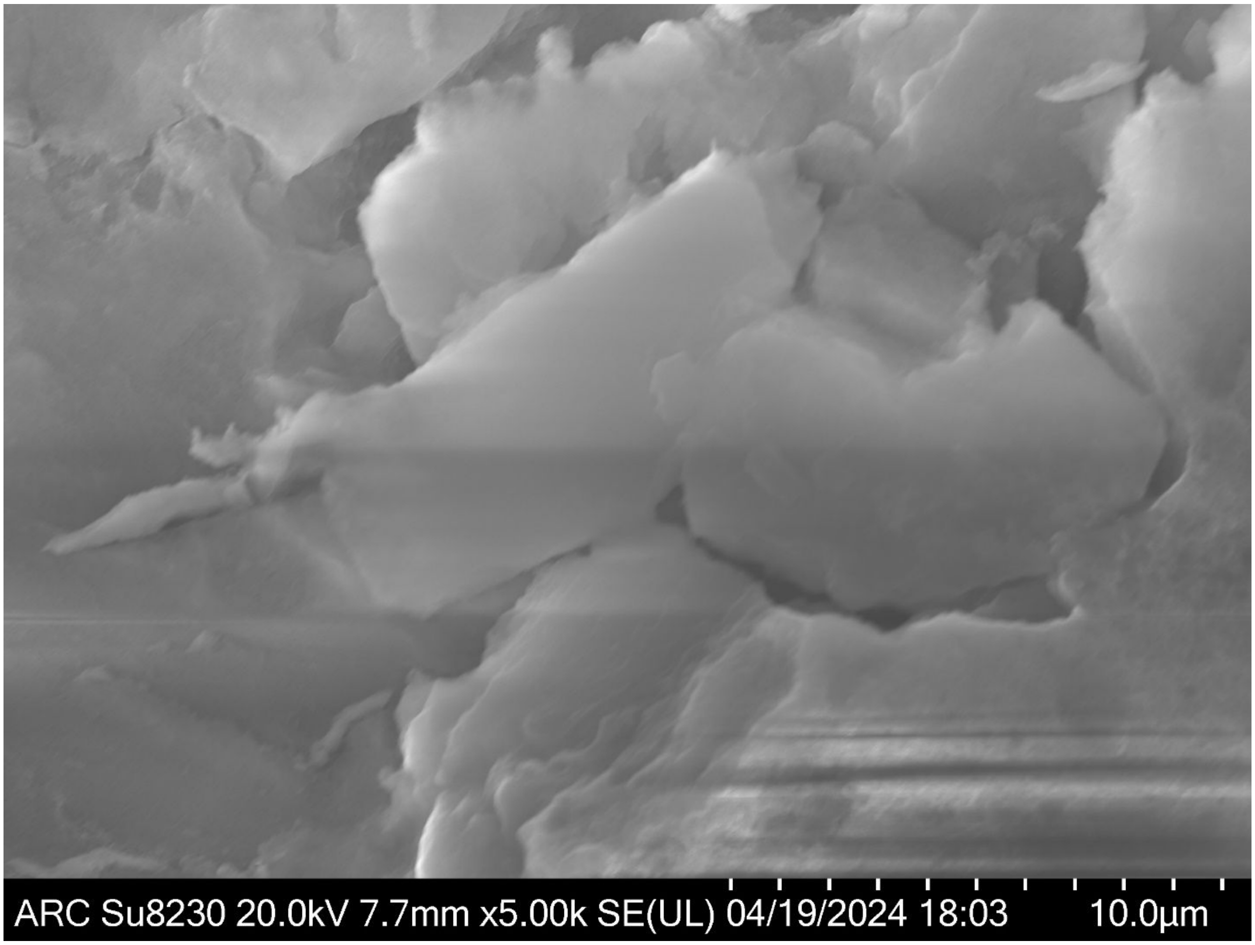

Figure S33. SEM of the RuKY-NO_3/OAc/NO_3 outer shell after calcination.

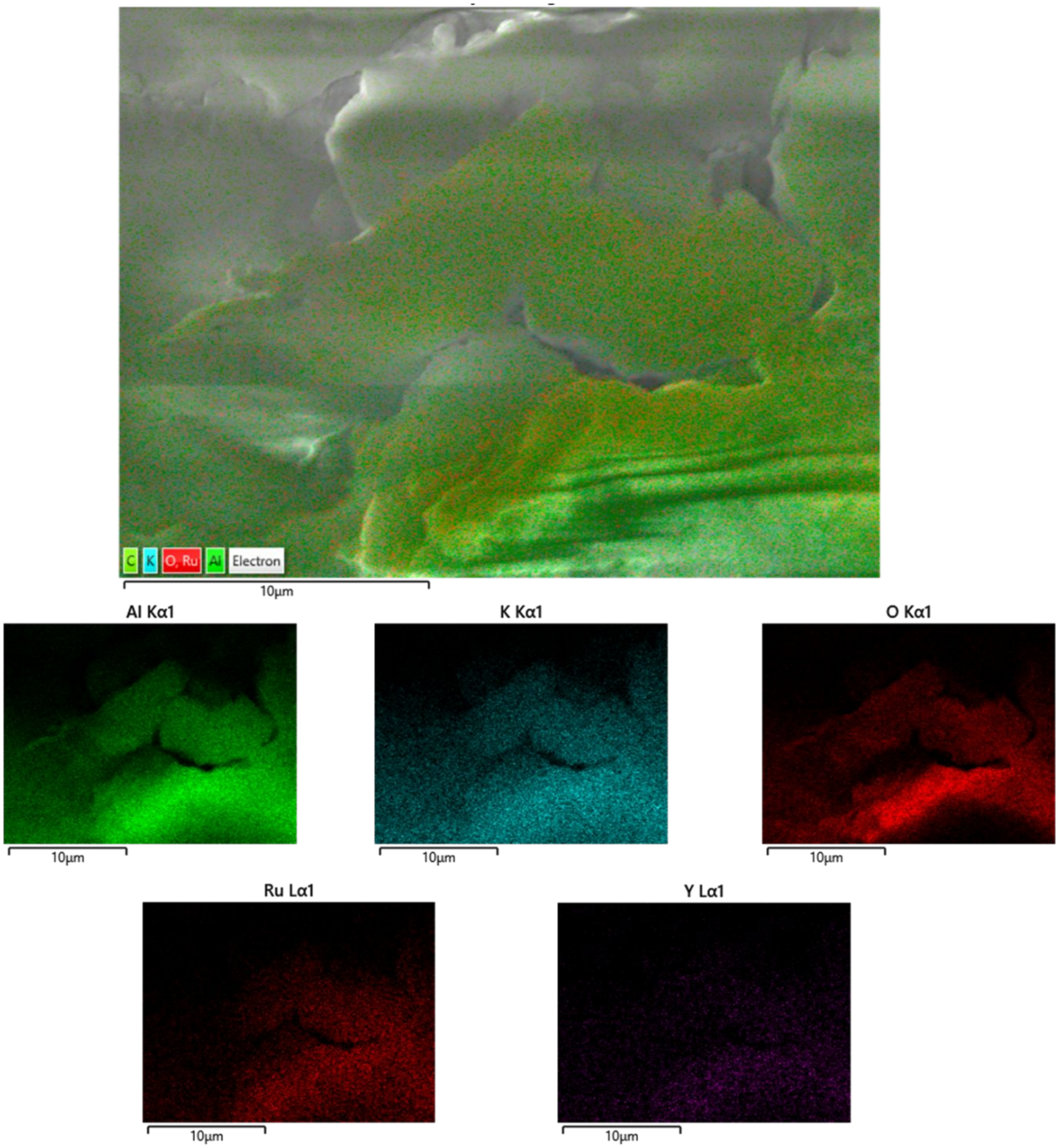

Figure S34. EDX overlay of the SEM shown in Figure S33.

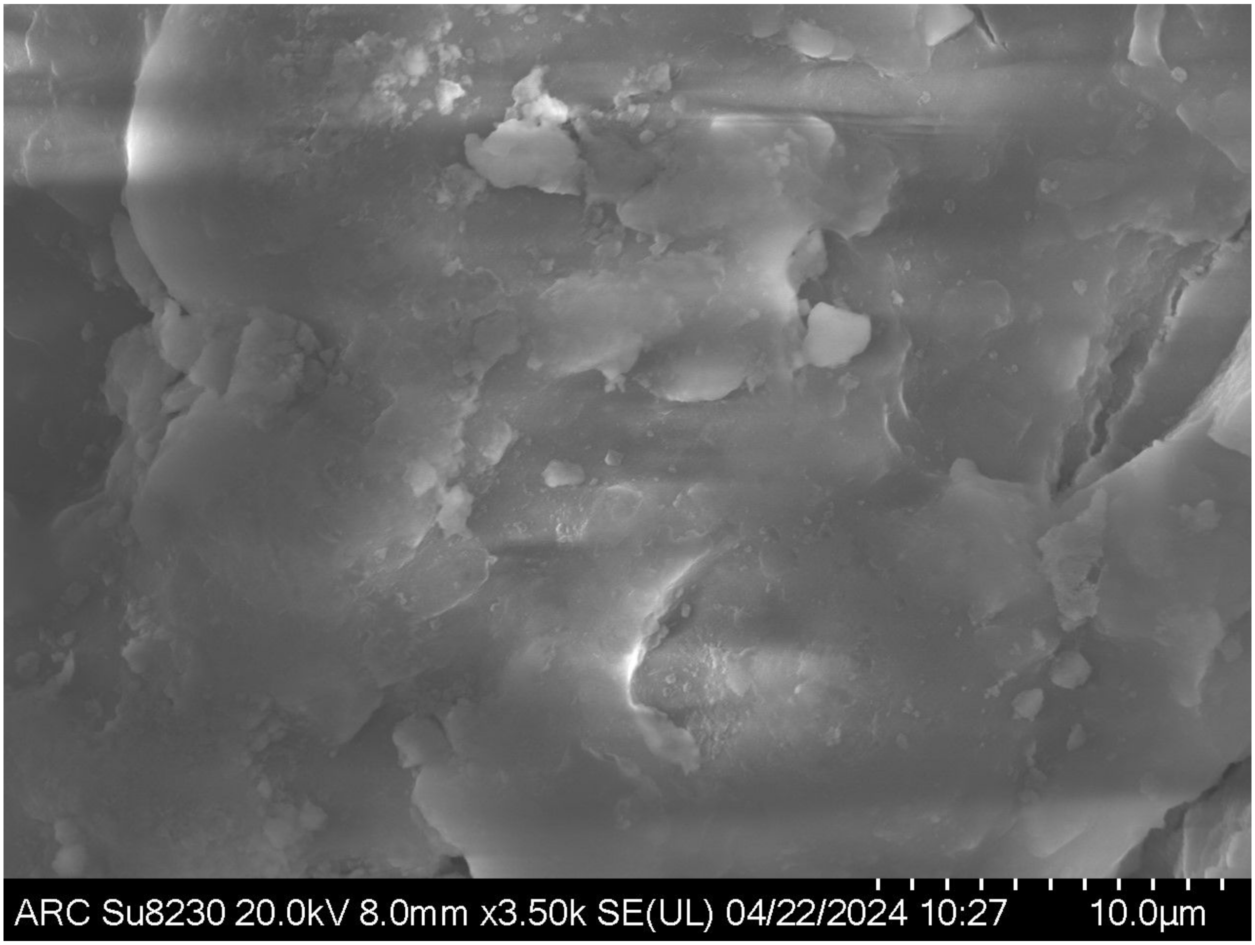

Figure S35. SEM of the RuKY-NO_3/OAc/NO_3 of the outer shell after reaction.

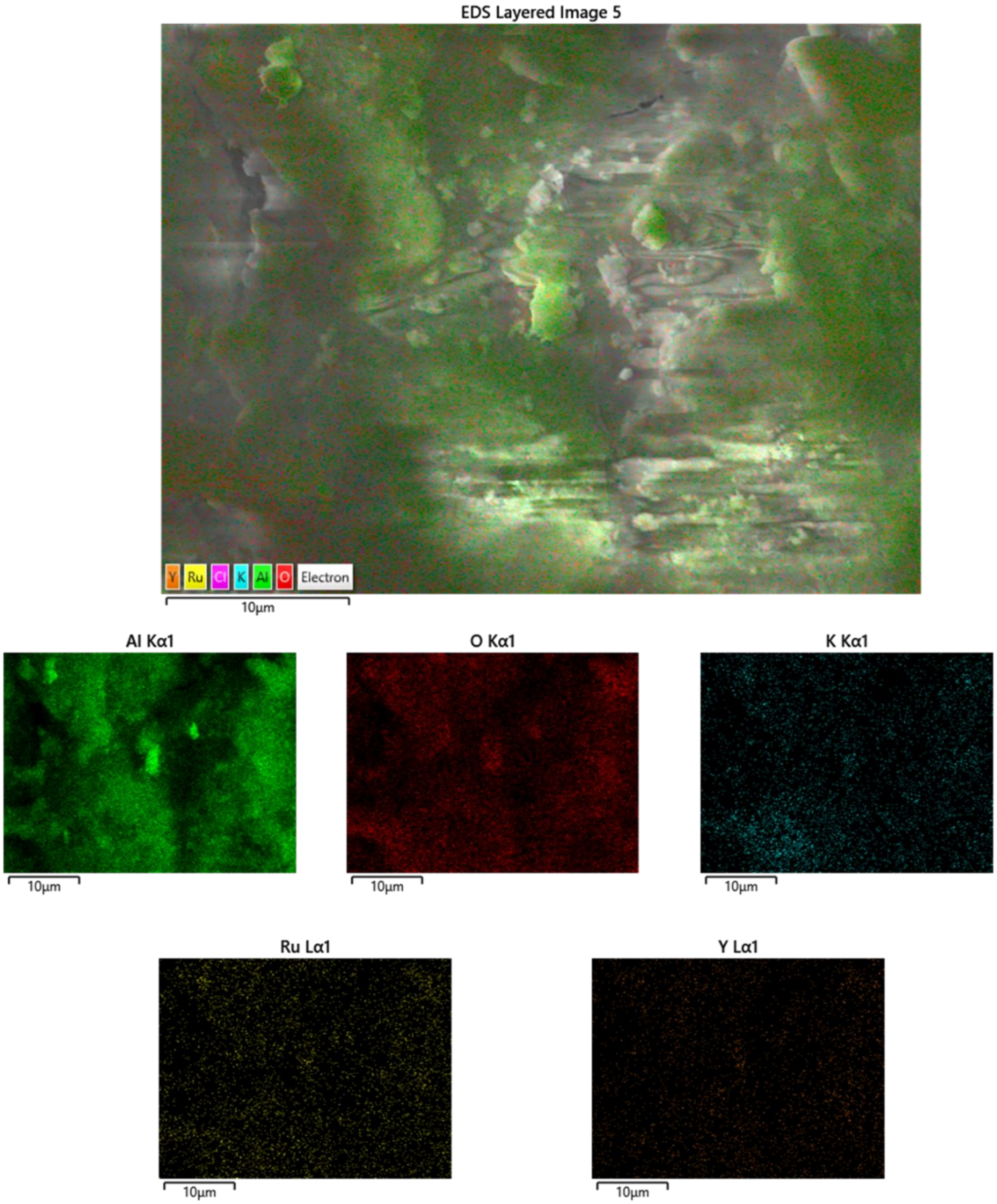

Figure S36. EDX overlay of the SEM shown in Figure S35.

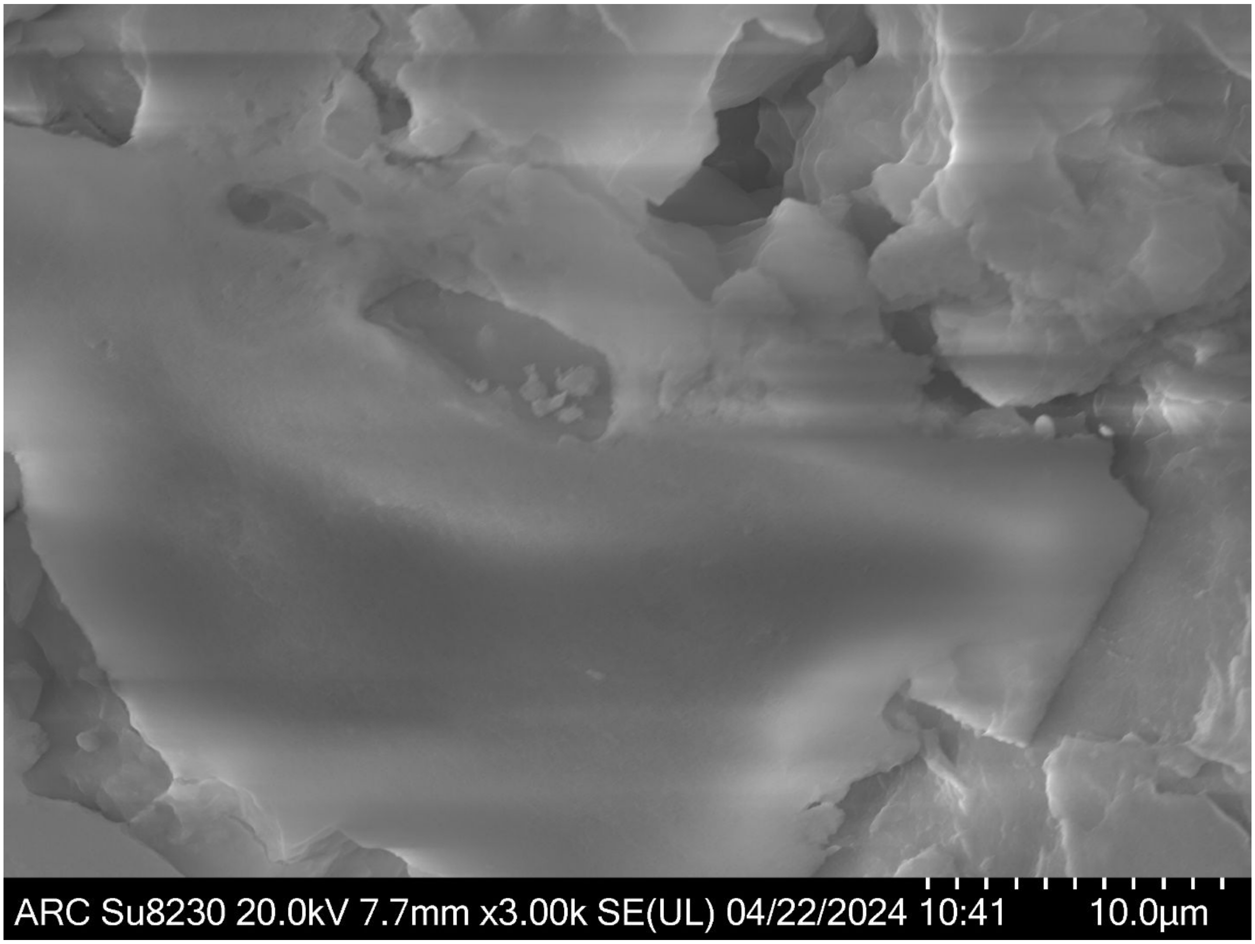

Figure S37. SEM of the cross section of the RuKY-NO_3/OAc/NO_3 after calcination

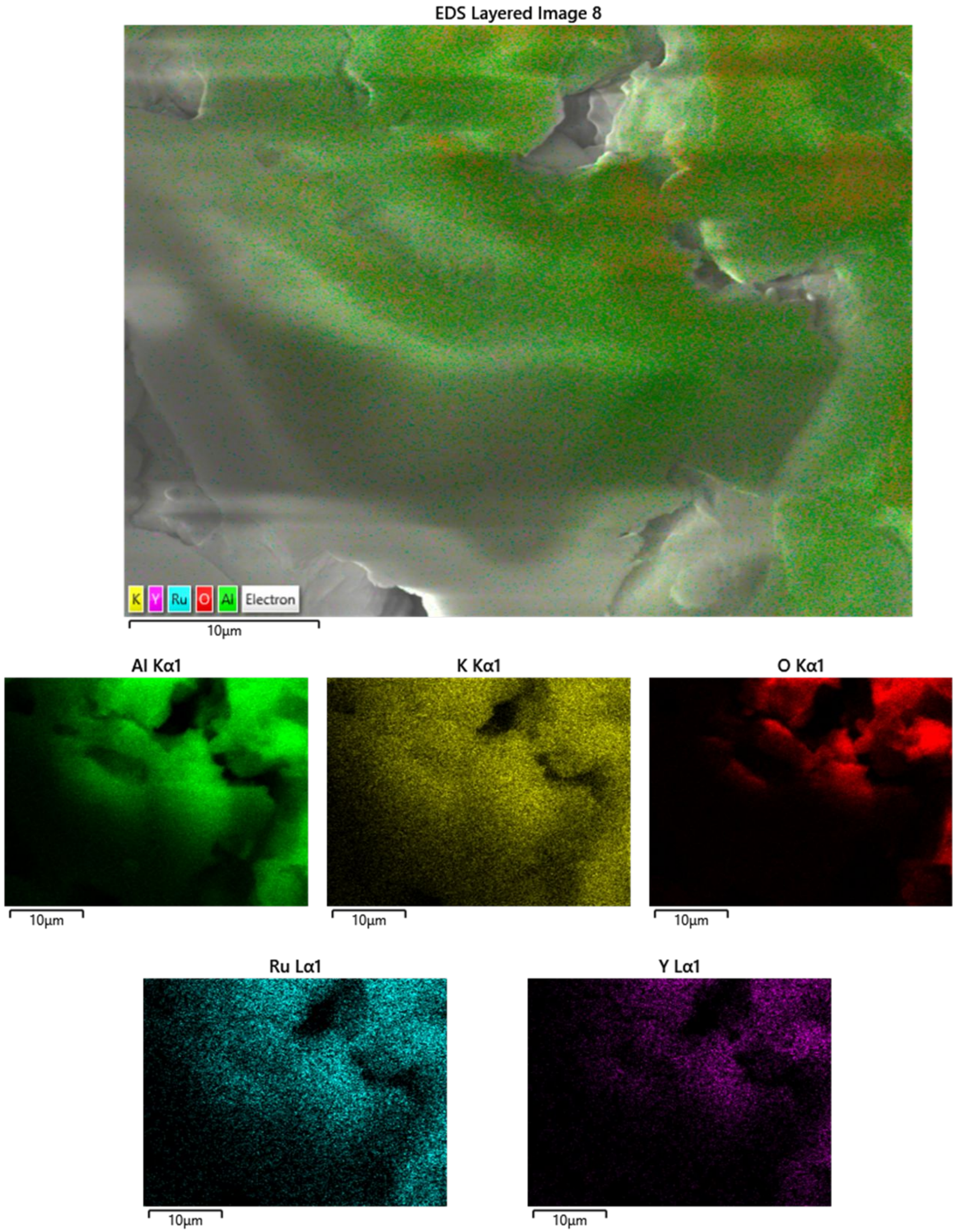

Figure S38. EDX overlay of the SEM shown in Figure S37.

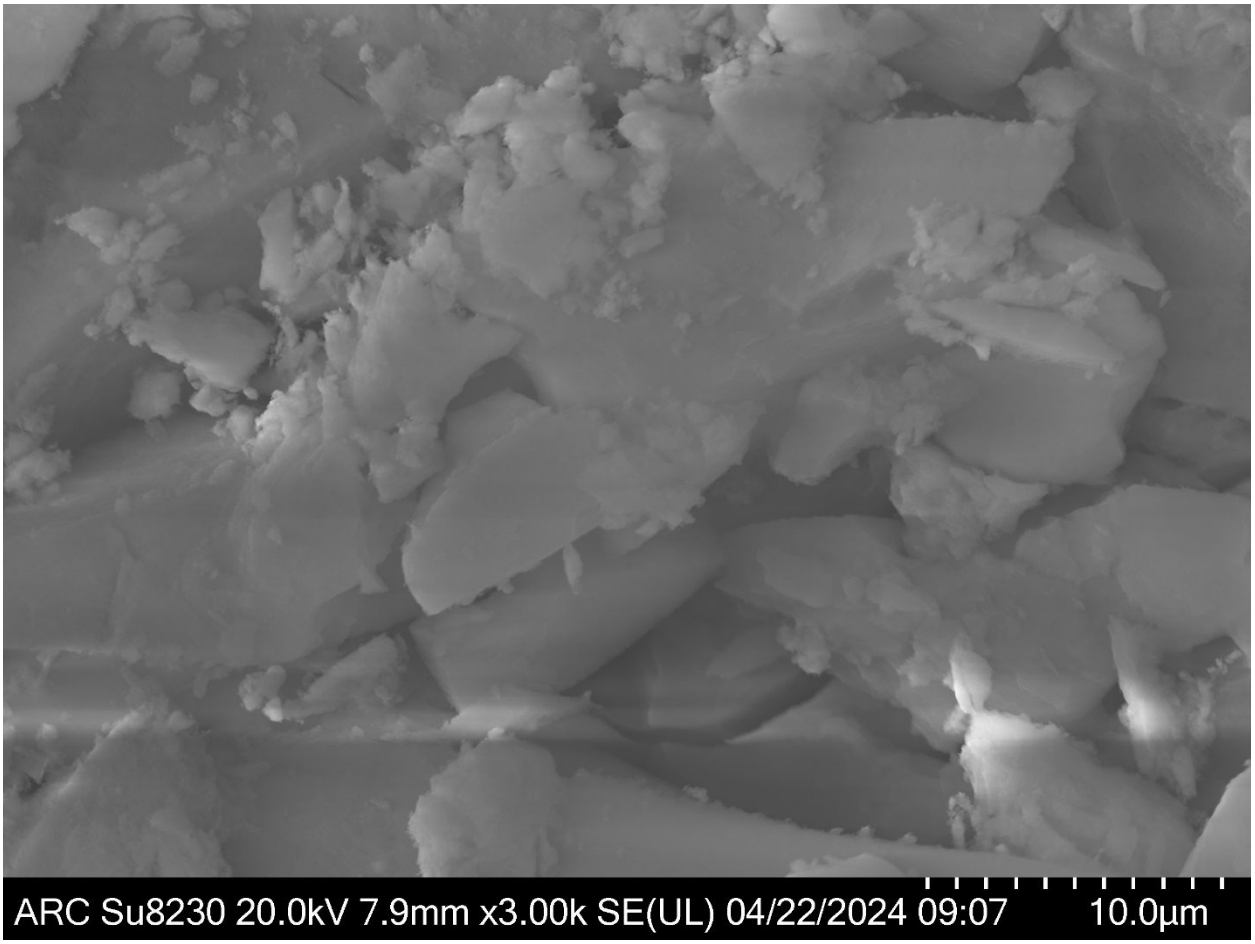

Figure S39. SEM of the cross section of the RuKY-NO_3/OAc/NO_3 after reaction.

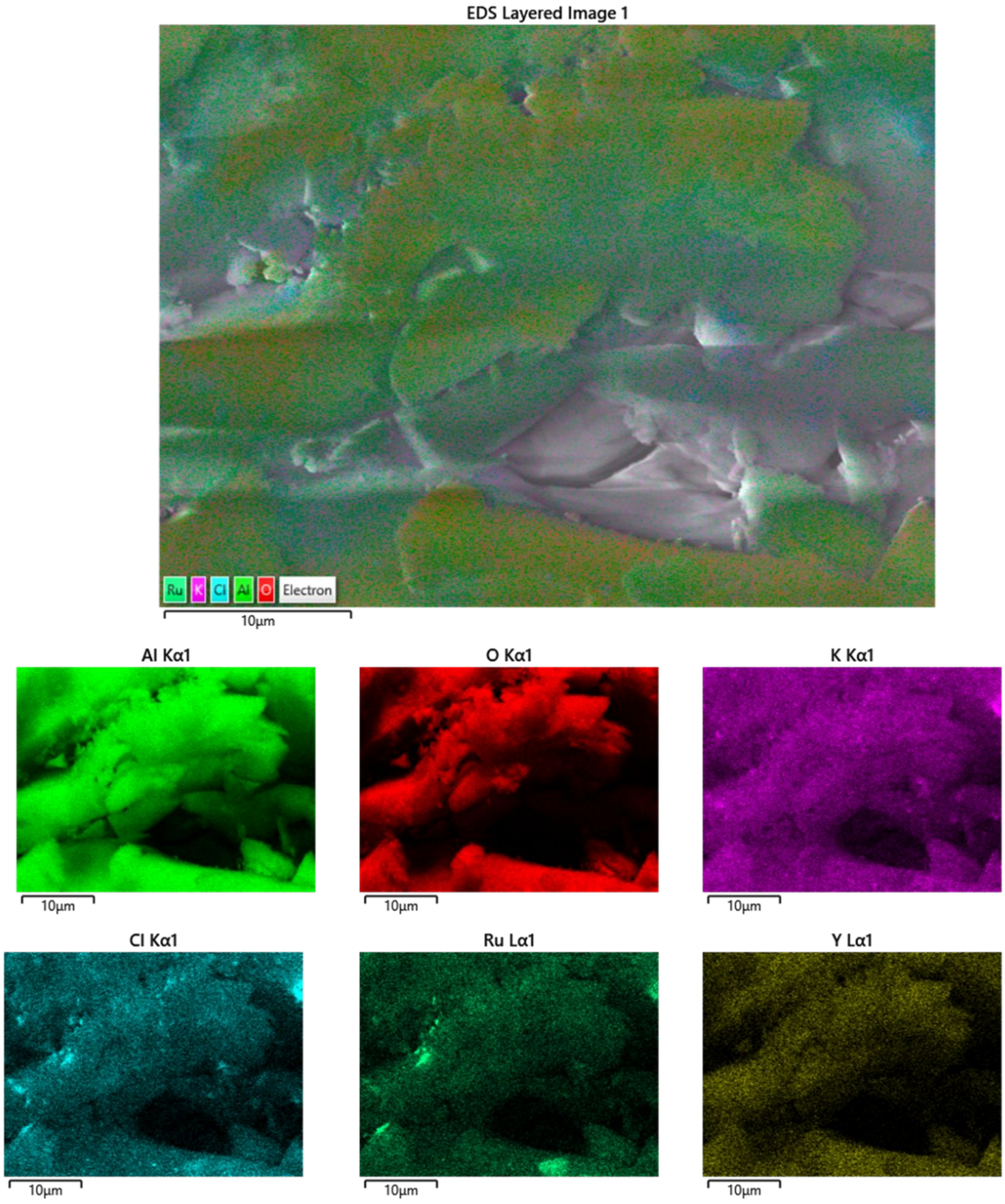

Figure S40. EDX overlay of the SEM shown in Figure S39.

Additional Data

Figure S41 shows the comparison of RuKY-NO_3/NO_3/NO_3 and RuKY-Cl/OAc/Cl as both are better performing catalysts of chloride free catalyst for the former and chloride-precursor catalyst for the latter. The reaction took place at 450°C , where both catalysts perform similarly to one another. However, upon increasing the amount of ammonia in the reagent feed, a difference becomes pronounced between the two catalysts. Specifically, after the catalyst becomes primarily NH_3, >80% NH_3, the conversion rates for the RuKY-NO_3/NO_3/NO_3 catalyst is higher than that of the RuKY-Cl/OAc/Cl catalyst. This shows that the chloride-free catalyst is able to better perform the catalysis than the chloride-precursor catalyst at 450°C with varying ammonia feed.

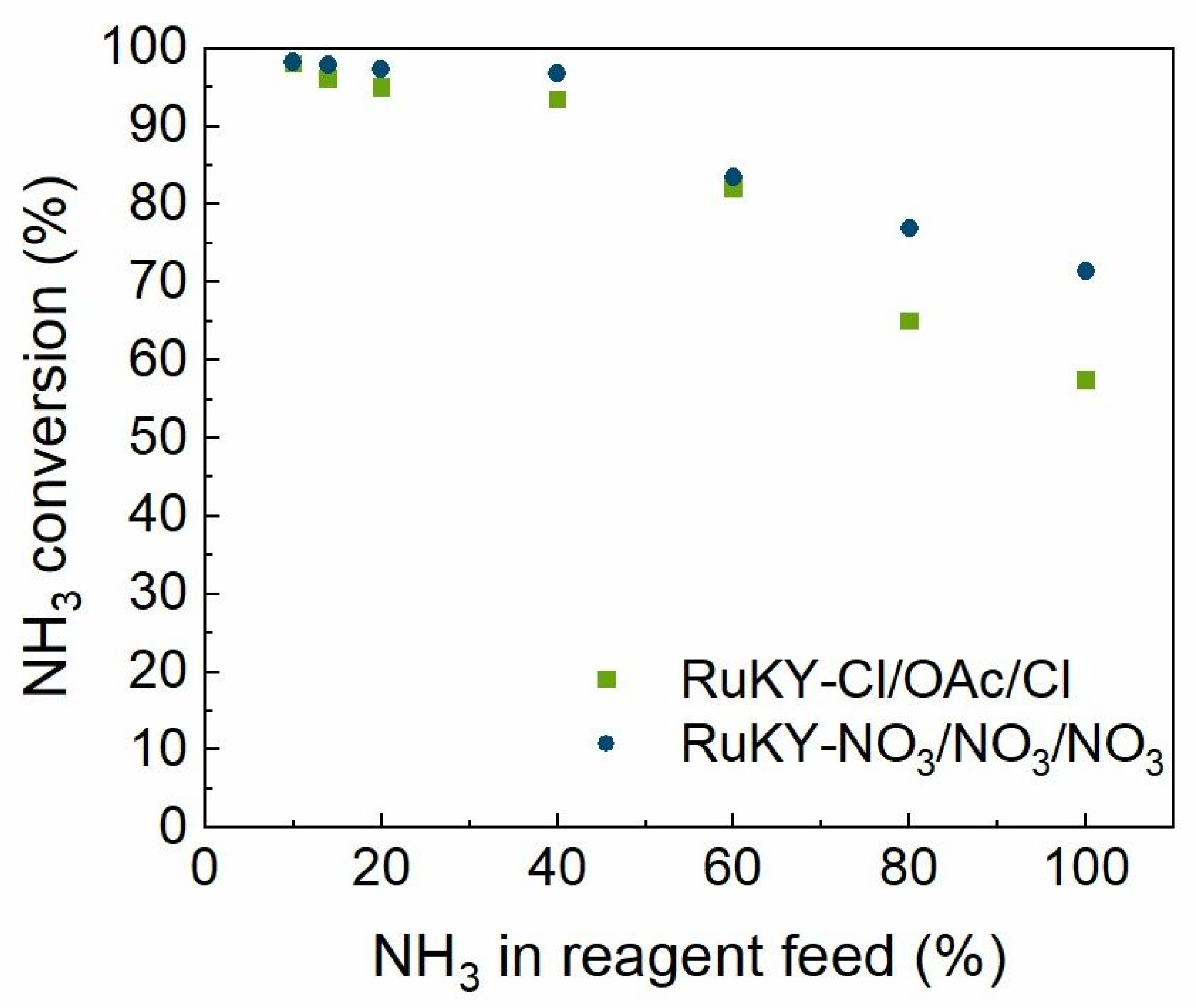

Figure S41. NH_3 conversion rate in comparison to the relative amount of ammonia in the system (conditions: 450°C, 50ml/min gas flow total with the remaining balance being Argon, 6000 ml·hr^{-1}·g_{cat}^{-1} - example if 50% NH_3 flow then it is 25mL/min NH_3 and 25mL/min Ar).

Ammonia Temperature Programmed Desorption (TPD)

Table S10. Breakthrough Ammonia Capacity for each catalyst.

**Catalyst**	**Breakthrough Ammonia Capacity (μmol NH_3/ g_{cat})**
RuKY-Cl/OAc/Cl	851
RuKY-Cl/NO_3/Cl	889
RuKY-NO_3/OAc/NO_3	896
RuKY-NO_3/NO_3/NO_3	1045

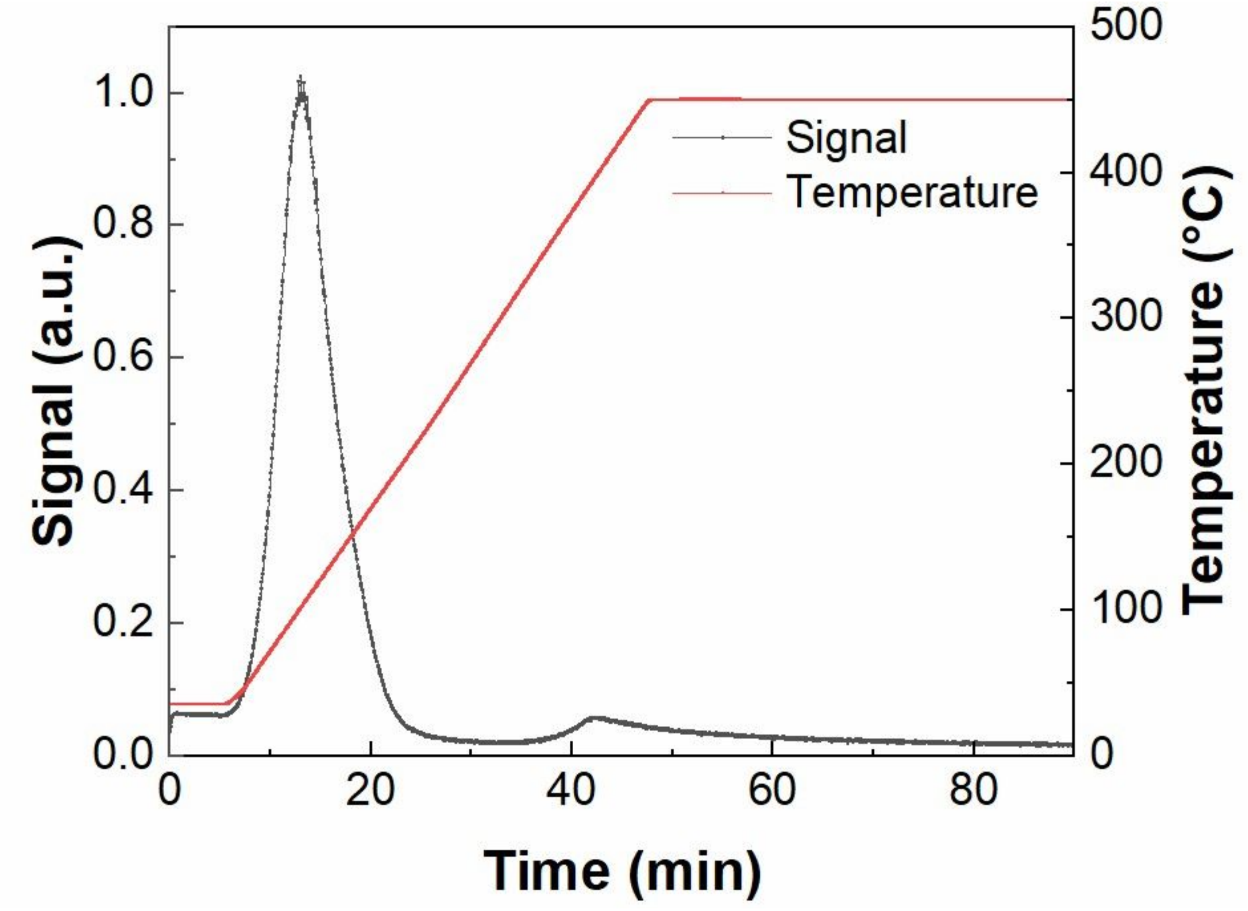

Figure S42. Ammonia Temperature Programmed Desorption of NH_3 on the RuKY-Cl/OAc/Cl catalyst.

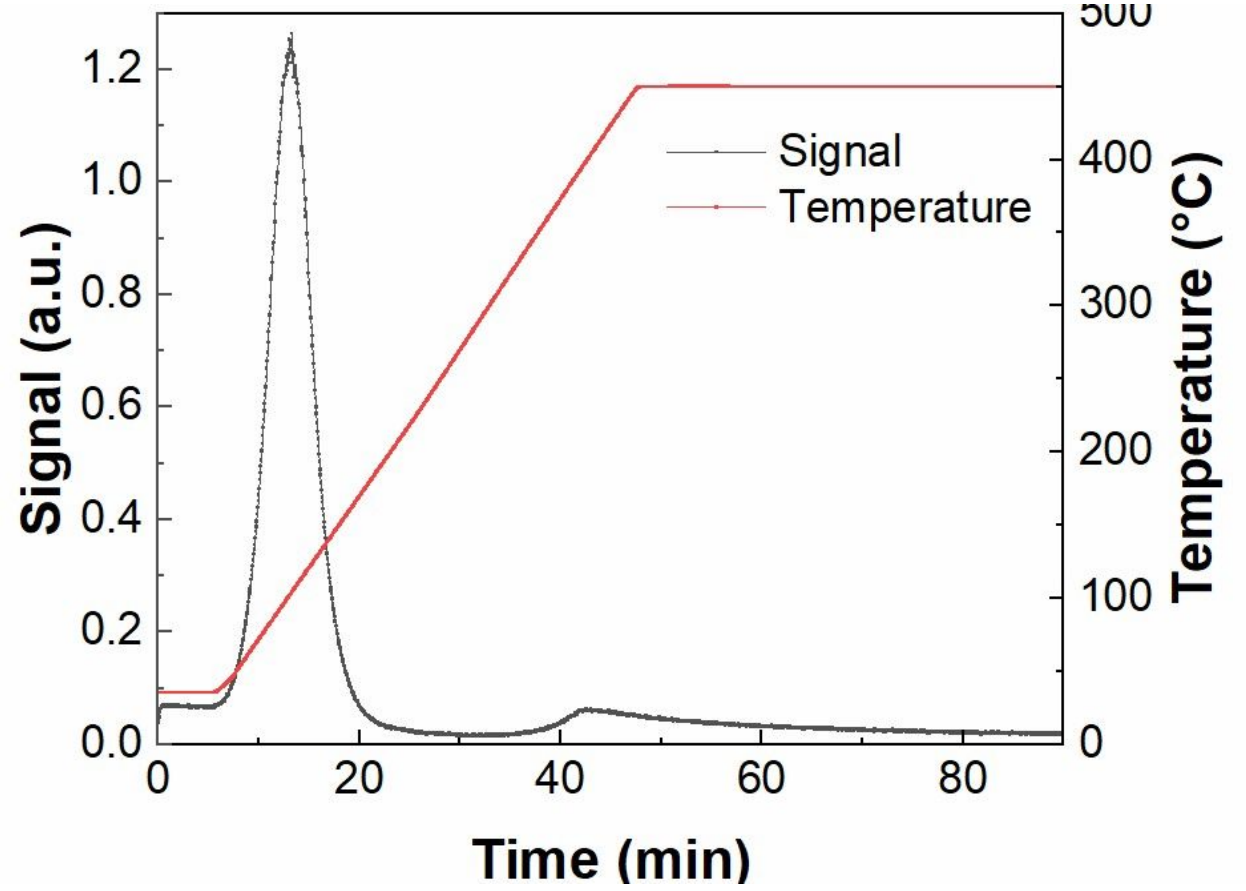

Figure S43. Ammonia Temperature Programmed Desorption of NH_3 on the RuKY-Cl/NO_3/Cl catalyst.

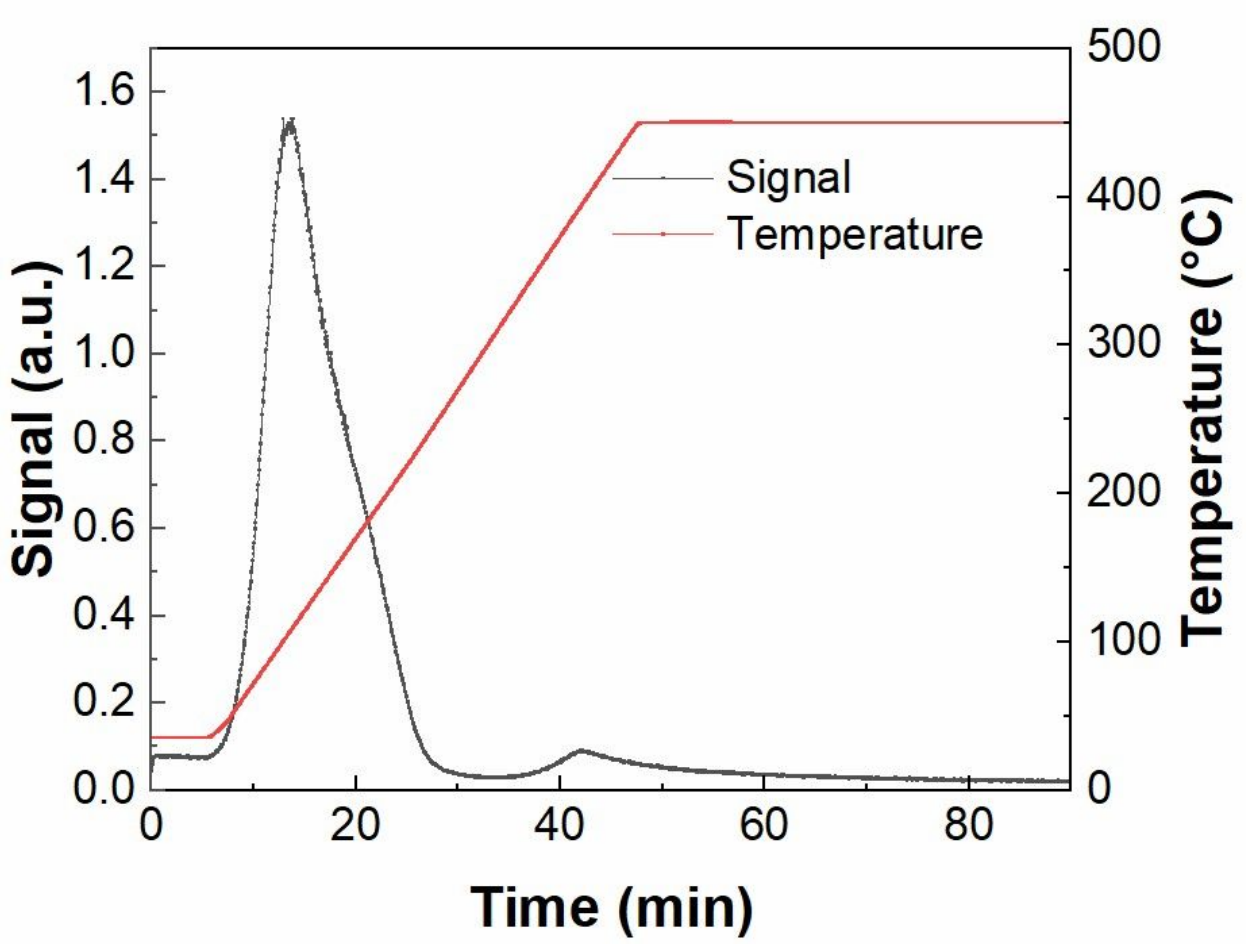

Figure S44. Ammonia Temperature Programmed Desorption of NH_3 on the RuKY-NO_3/OAc/NO_3 catalyst.

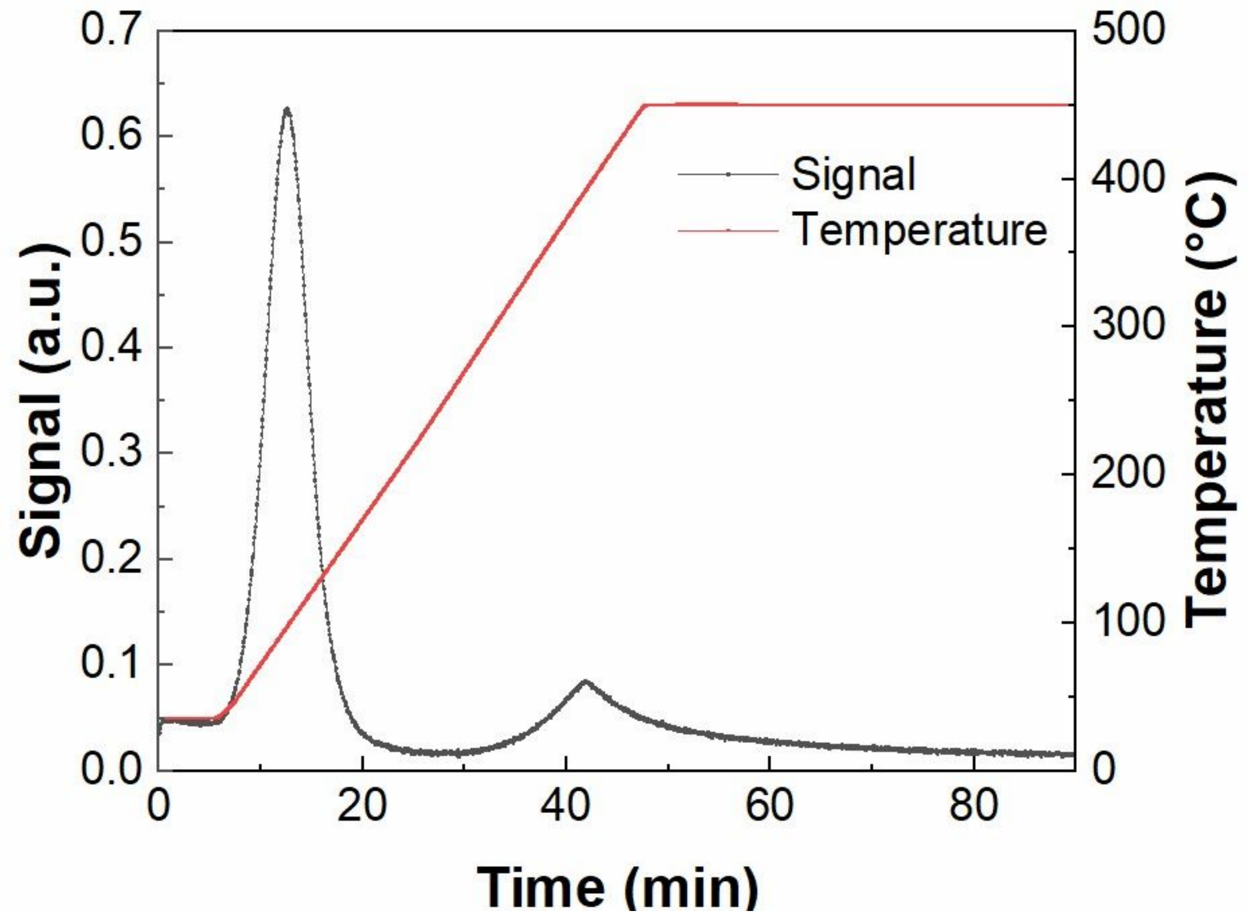

Figure S45. Ammonia Temperature Programmed Desorption of NH_3 on the RuKY-NO_3/NO_3/NO_3 catalyst.

Mass Spectrometry

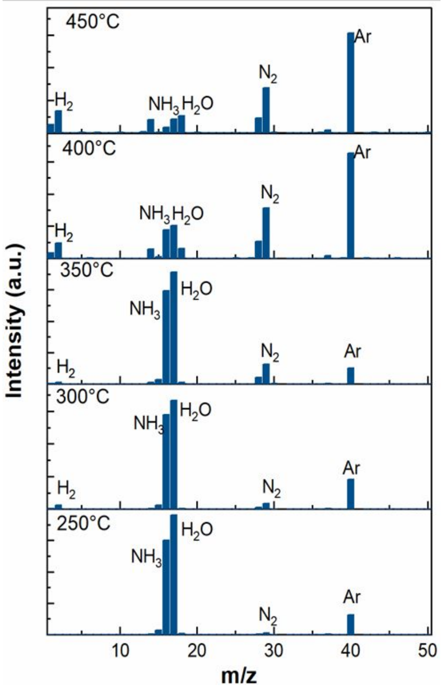

Figure S46. A representative example of a mass spectrometry spectra that was used to calculate yields (conditions: 500 mg RuKY, 30ml/min NH_3).

Activation Energy

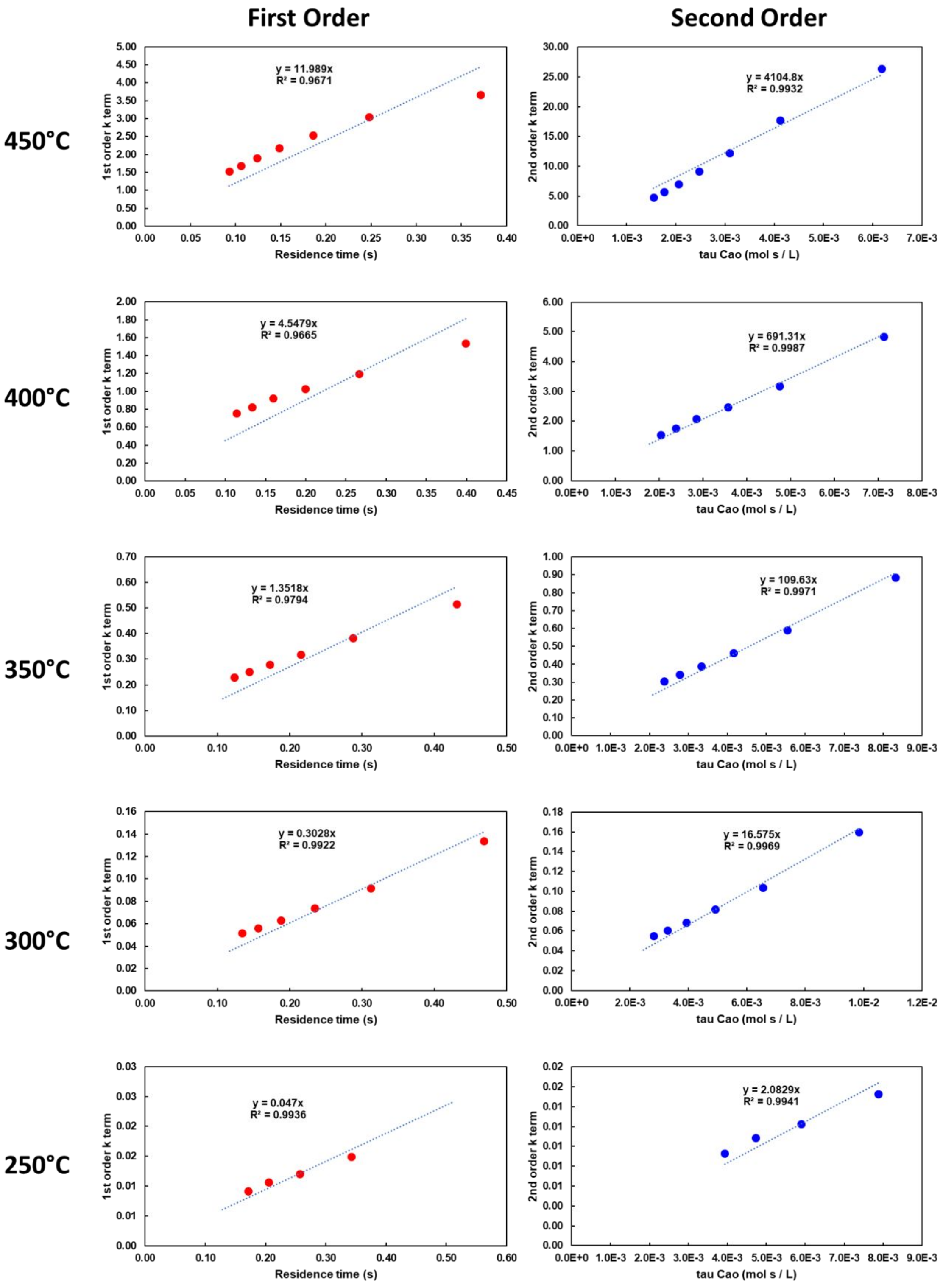

Figure S47. Rate plots for the ammonia cracking catalyst, RuKY-NO_3/NO_3/NO_3.

Figure S47 shows the reaction rate plots for the RuKY-NO_3/NO_3/NO_3 catalyst at the five temperatures tested. The graphs for both first and second order are shown to demonstrate that the reaction order is second as the data was fit well in the second order reaction graphs. The experiments were run under pure ammonia streams to calculate conversion rates. This was done to ensure that the conversions were not done at equilibrium, which would decrease the accuracy of the results. The conversion rates were then used to calculate the y-terms of these plots with the equation for the second order reaction y-term being shown in Equation 2. The slope was then taken as the rate constant and utilized for the Arrhenius plot that is shown in figure 5. The other catalysts were performed in a similar way.
